# Harnessing Toluene Solvent as a Reactant: Regioselective Benzyl Radical Addition to Multi‐π 1,5‐Enynes in a Copper‐Catalyzed Cascade to Indenes

**DOI:** 10.1002/chem.202503120

**Published:** 2026-01-28

**Authors:** Saideh Rajai‐Daryasarei, Fatemeh Chahardehi, Mohammad Mahdi Sharifani, Morteza Jamshidi, Robert Stranger, Frank Rominger, Alireza Ariafard, Saeed Balalaie

**Affiliations:** ^1^ School of Chemistry College of Science University of Tehran Tehran Iran; ^2^ Peptide Chemistry Research Institute K. N. Toosi University of Technology Tehran Iran; ^3^ Research School of Chemistry Australian National University Canberra ACT Australia; ^4^ Organisch‐Chemisches Institut der Universität Heidelberg Heidelberg Germany

## Abstract

C─H activation of toluene generates a benzyl radical that undergoes C─C coupling, converting an abundant solvent into carbon building blocks. A major challenge, however, is to direct the highly reactive benzyl radical to add regioselectively to a multi‐π system in a way that initiates a cascade leading to complex products. Here, we achieved this goal for the first time, to our knowledge, and demonstrated that polysubstituted indene derivatives can be efficiently obtained through a three‐component cascade radical cyclization of 1,5‐enynes with toluene solvent and benzoic acids in the presence of a copper catalyst and TBHP oxidant. Notably, the same indene products were also obtained when aldehydes were used in place of benzoic acids. Our combined experimental and computational investigations fully elucidated the reaction mechanism, showing that it proceeds through a Cu(I/II/III) redox cycle. This study further revealed that benzyl radicals are generated at the outset of the reaction but are highly reactive and prone to deactivation unless they undergo rapid and regioselective addition to the multi‐π system. We show that these requirements are satisfied only when both terminal carbons of the alkene moiety in the 1,5‐enyne bear strong electron‐withdrawing substituents such as CN or CO_2_Me.

## Graphical abstract

A copper‐catalyzed three‐component radical cascade of 1,5‐enynes with toluene and benzoic acids enables regioselective benzyl radical addition to multi‐Π systems, efficiently delivering polysubstituted indenes from an abundant solvent feedstock.

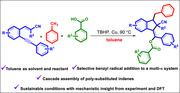

## 1 Introduction

Toluene, an abundant and inexpensive petroleum‐derived solvent, represents an attractive yet underutilized feedstock for the synthesis of complex molecules. Direct C─H functionalization offers a powerful approach to convert toluene from a simple solvent into versatile carbon building blocks. Given the high significance of such transformations, extensive efforts have been directed toward developing modern strategies that harness toluene directly as a reactive substrate rather than merely as an inert solvent [1, 2, 3, 4, 5, 6, 7, 8].

**SCHEME 1:**
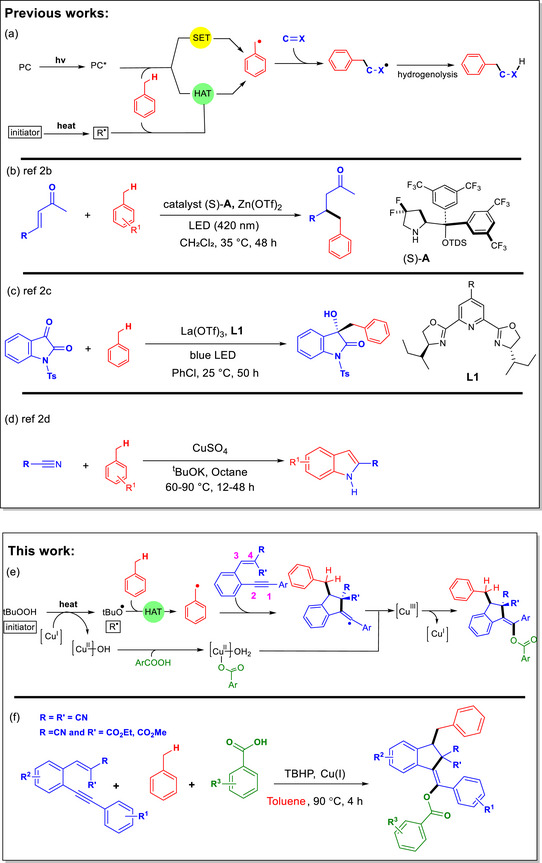
Representative strategies for benzyl radical generation from toluene and subsequent bond‐forming reactions: (a) general approaches via photoredox or thermal initiation; (b) addition of benzyl radicals to enals via iminium activation developed by Melchiorre et al. (ref. [10]); (c) addition to isatins via photochemical SET/HAT pathways developed by Jiang et al. (ref. [11]); (d) addition to benzonitriles and alkyl nitriles mediated by CuSO_4_/*t*BuOK developed by Kang et al. (ref. [12]); (e,f) this work: regioselective benzyl radical addition to 1,5‐enynes followed by cascade cyclization and benzoate incorporation enabled by a Cu(I) catalyst and TBHP oxidant.

One of the most widely used strategies for the functionalization of toluene derivatives relies on photochemical or thermal initiation (vide infra) to generate highly reactive benzyl radicals as key intermediates. These radicals readily add to unsaturated C═X bonds, and the resulting intermediates are typically quenched through a termination step such as hydrogenolysis, thereby furnishing the corresponding functionalized toluene derivatives (Scheme 1a) [9, 10, 11, 12, 13, 14].

In photocatalytic systems, the process begins with excitation of the photocatalyst (PC → PC*) (Scheme 1a). The excited state can generate benzyl radicals from toluene through two distinct pathways: (i) single‐electron transfer (SET), in which PC* oxidizes toluene and the resulting radical cation is deprotonated by an appropriate base to furnish the benzyl radical, or (ii) hydrogen atom transfer (HAT), in which PC* or a derived radical abstracts a benzylic hydrogen atom to produce the benzyl radical [15, 16, 17, 18, 19, 20, 21]. In thermal systems, the reaction is initiated by homolysis of a radical precursor (initiator) to generate a reactive radical R^•^, which subsequently abstracts a benzylic hydrogen atom from toluene via a HAT process [22, 23, 24, 25, 26, 27, 28, 29, 30, 31], thereby affording the benzyl radical. In addition to this pathway, alternative mechanisms have been reported in which benzyl radical formation proceeds through base‐assisted deprotonation of toluene followed by a metal‐mediated single‐electron transfer (SET) process [12, 32, 33, 34, 35, 36, 37, 38, 39, 40, 41, 42]. Once generated, benzyl radicals can undergo addition to a range of unsaturated bonds, as depicted in Scheme 1a.

A classic example of benzyl radical addition to a C═C bond was reported by Melchiorre and coworkers [10], who showed that visible‐light excitation of an iminium ion oxidizes toluene through a sequential proton‐coupled electron transfer (SET followed by deprotonation), generating benzyl radicals that add to enals to furnish β‐benzylated aldehydes (Scheme 1b).

Another representative example is the addition of benzyl radicals to a C═O π‐bond, as demonstrated by Jiang and coworkers [11], who achieved direct coupling of toluene‐derived benzyl radicals with isatins under photochemical conditions to deliver 3‐hydroxy‐3‐benzyl‐substituted oxindoles, for which both SET and HAT pathways were proposed (Scheme 1c).

In a related approach, Kang and coworkers [12] demonstrated that benzyl radicals generated from toluene can add to C≡N π‐bonds, enabling direct coupling with benzonitriles and alkyl nitriles under CuSO_4_/*t*BuOK conditions to form indole derivatives (Scheme 1d).

Although diverse synthetic strategies have been reported for C─H bond functionalization of toluene [43, 44, 45], none to date have demonstrated the controlled addition of benzyl radicals to multi‐π systems such as 1,5‐enynes. This limitation may arise from the intrinsically high reactivity of the benzyl radical, which often results in nonregioselective attack at multiple sites or rapid deactivation of the radical. For a synthetically useful outcome, the benzyl radical must (i) add regioselectively to a single site within the multi‐π framework, and (ii) react rapidly enough to avoid deactivation through side reactions. In this study, we have overcome these challenges by achieving the regioselective addition of benzyl radicals to 1,5‐enynes, thereby initiating cascade processes that furnish polysubstituted indene derivatives (Scheme 1e,f). We found that the two requirements discussed above are fulfilled when both terminal positions of the alkene moiety in the 1,5‐enyne substrates bear strongly π‐accepting substituents (such as CN or CO_2_Et).

Interestingly, under our reaction conditions the termination step proceeds via benzoate incorporation rather than conventional hydrogenolysis, a distinctive outcome whose mechanistic basis is examined in this study.

As schematically outlined in Scheme 1e,f, the presence of benzoic acid additives, a Cu(I) catalyst, and *t*BuOOH (TBHP) oxidant is essential for this transformation, and DFT calculations were employed to elucidate the mechanistic role of each species.

The present study not only introduces a new methodology for achieving regioselective benzyl radical addition to multi‐π systems but also provides a novel strategy for the synthesis of indene derivatives, a class of compounds with broad significance as precursors of metallocene complexes for catalytic polymerization and as valuable scaffolds in drug discovery and development [46, 47, 48, 49, 50]. Accordingly, the indene products obtained in this study represent versatile intermediates that offer opportunities for further transformation toward applications in metallocene‐based catalysis and medicinal chemistry. Finally, given the central role of C─H bond activation in modern synthetic chemistry [51, 52, 53, 54, 55, 56, 57, 58], the present work makes a significant contribution to the continued advancement of this pivotal field.

## 2 Results and Discussion

Our study was initiated with (phenylethynyl)benzylidene malononitrile **1** and 4‐methylbenzoic acid **2** as the model substrates to optimize the reaction conditions (Table 1). In the first attempt, 1,5‐enyne **1** was treated with 4‐methylbenzoic acid **2** in the presence of CuI (20 mol%) as catalyst and TBHP (3.0 equiv.) as oxidant in toluene at 90 °C. To our delight, the desired product **3b** was obtained in 41% yield when 1,5‐enyne **1** and 4‐methylbenzoic acid **2** were subjected to cyclization in toluene solvent (entry 1). Encouraged by this result, we next examined the effect of TBHP loading on the reaction. When the amount of TBHP was changed to 5.0 equiv. and 6.0 equiv., the desired product **3b** was obtained in 73% and 61% yields, respectively. We then systematically screened cascade cyclization by varying the solvent, catalyst, oxidant, temperature, and time. Oxidants such as K_2_S_2_O_8_ and DTBP proved unsuitable for this transformation (entries 4 and 5). We next examined common copper catalysts including Cu(OAc)_2_, CuCl, CuBr, and Cu_2_O; however, all of them suppressed the formation of the desired product **3b** (entries 6–9). Remarkably, when the reaction was performed without CuI, no product was formed, showing that the catalyst is essential for this transformation (entry 10). Screening solvent mixtures consisting of 0.4 mL of CH_3_CN, DMF, DMSO, DCE, 1,4‐dioxane, THF, or PhCl with 0.1 mL of toluene showed that these combinations dramatically hampered the reaction, whereas pure toluene proved to be the optimal medium (entries 11–17). Next, when the amount of toluene was increased to 1.0 mL, the yield of product **3b** decreased to 61% (entry 18). When the reaction was carried out at 120 °C, product **3b** was obtained in only 10% yield (entry 19), confirming 90 °C as the optimal temperature. Interestingly, the absence of 4‐methyl benzoic acid **2** completely suppressed the formation of cyclization product **3b** (entry 20). This observation underscores the crucial role of 4‐methylbenzoic acid as a cocatalyst and starting material in facilitating the synthesis of product **3b**. The optimal conditions were ultimately identified as those in Table 1, entry 2, which afforded product **3b** in 73% yield.

**TABLE 1:** Optimization of reaction conditions.^a^

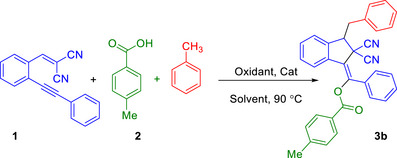				
Entry	Oxidant	Catalyst (20 mol%)	Solvent	Yield (%)^b^
1	TBHP (3)	CuI	PhCH_3_	41
2	**TBHP (5)**	**CuI**	**PhCH_3_**	**73**
3	TBHP (6)	CuI	PhCH_3_	61
4	K_2_S_2_O_8_	CuI	PhCH_3_	0
5	DTBP	CuI	PhCH_3_	Trace
6	TBHP	Cu(OAc)_2_	PhCH_3_	31
7	TBHP	CuCl	PhCH_3_	Trace
8	TBHP	CuBr	PhCH_3_	0
9	TBHP	Cu_2_O	PhCH_3_	0
10	TBHP	—	PhCH_3_	0
11^c^	TBHP	CuI	CH_3_CN	NR
12^c^	TBHP	CuI	DMF	NR
13^c^	TBHP	CuI	DMSO	NR
14^c^	TBHP	CuI	DCE	21
15^c^	TBHP	CuI	1,4‐Dioxane	NR
16^c^	TBHP	CuI	THF	NR
17^c^	TBHP	CuI	PhCl	28
18^d^	TBHP	CuI	PhCH_3_	61
19^e^ 20^f^	TBHP TBHP	CuI CuI	PhCH_3_ PhCH_3_	10 NR

^a^ Reaction condition: 1 (0.05 mmol), 2 (0.1 mmol), solvent (0.5 mL), at 90 °C for 4 h.

^b^ Isolated yield

^c^ Solvent: Toluene (0.4:0.1)

^d^ Solvent (1.0 mL)

^e^ At 120 °C

^f^ Without 4‐methyl benzoic acid

Having determined the optimal reaction conditions, the scope and limitation of this transformation were further explored (Table 2). Fortunately, this approach was successful across a broad range of starting materials, regardless of the electron density or positional variation of the substituents. First, a variety of carboxylic acid including electron‐donating (Me), halogen (Cl, Br, I), as well as electron withdrawing groups (CF_3_) successfully reacted with 1,5‐enyene and toluene, affording the indanones **3a–f** and in moderate to good yields (Table 2). In addition, the structure of **3b** was unambiguously confirmed by X‐ray crystallographic analysis (see the Supporting Information for details) [59], with ellipsoids drawn at the 50% probability level (Table 2). Surprising, indole‐2‐carboxylic acid also proved to be suitable substrate in this reaction, and corresponding product **3** **g** was delivered with 61% yield. Notably, 1,5‐enynes bearing electron‐donating groups (Me, OMe), electron withdrawing groups (CN) and as well as halogen group (F) were compatible with this reaction, affording moderate to good yields of the expected products **3h‐y**. It is worth noting that this method was effectively applied to 1‐naphthoic acid as starting material, and desired products **3m** and **3q** were obtained with 85% and 77% yields, respectively. Moreover, alkyl 2‐cyano‐3‐(2‐arylethynyl)phenyl)acrylates were also compatible in the reaction, giving target products **3z‐b'** with 67%‐88% yields. Notably, 2‐phenylacetic acid was compatible with this transformation, and corresponding product **3c'** was isolated with 67% yield. Both *m*‐xylene and *p*‐xylene were found to be suitable replacements for toluene, serving as both solvent and benzyl radical sources, and afforded the corresponding products **3d′** and **3e′** in 90% and 88% yield, respectively. It should be mentioned, a series of starting materials such as 3‐NO_2_, 4‐NO_2_‐benzoic acid and 2‐iodotoluene were tested under current conditions and regretfully, corresponding products **3f'‐h'** were not detected for these substrates. Aliphatic carboxylic acids such as acetic acid and propionic acid were also examined; however, these substrates led to complex mixtures and no isolable indene products **3i'** and **3j'** were obtained. In all cases, the reactions proceeded with complete diastereoselectivity, and only a single diastereomer was detected by ^1^H and ^13^C NMR analysis.

**TABLE 2:** Synthesis of polysubstituted indene‐2,2‐dicarbonitrile derivatives.^a^, ^b^

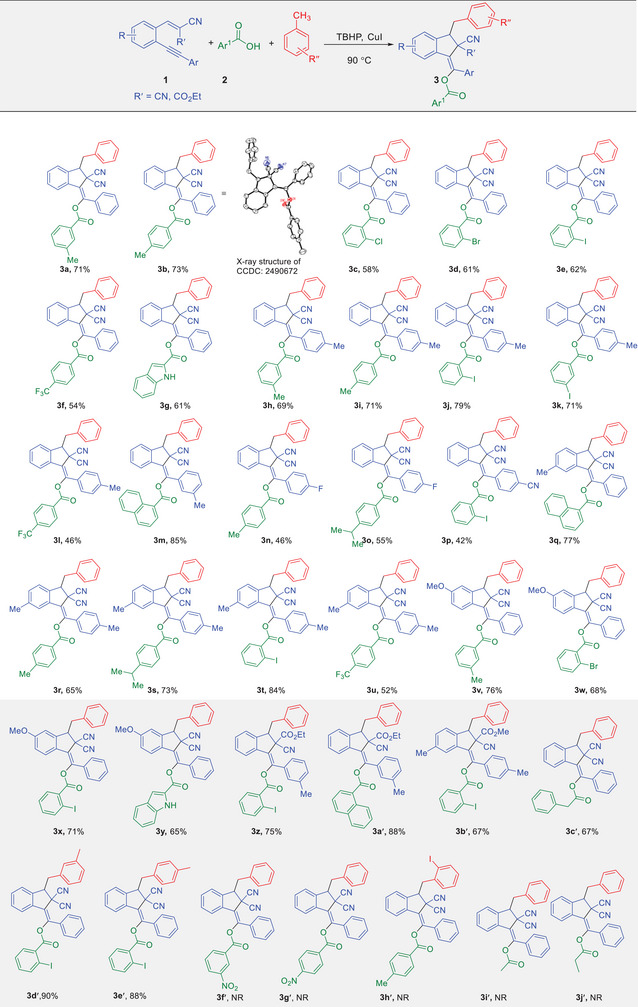

^a^ Reaction conditions: 1,5‐enynes 1 (1.0 equiv.), benzoic acids 2 (2.0 equiv.), TBHP (5.0 equiv.), and CuI (20 mol%) in 0.5 mL of PhCH_3_ at 90°C for 4 h

^b^ Isolated yields

Interestingly, we found that the scope of this transformation is not limited to the use of benzoic acids as cocatalysts and reactants. When aldehydes were employed instead of acids, the same indene products were obtained under the standard conditions (Table 3). The fact that aldehydes can replace benzoic acids yet furnish the same indene products indicates that aldehydes are oxidized in situ to the corresponding acids under the reaction conditions. The resulting acids then serve as true cocatalysts and reactants in the catalytic cycle. The mechanistic details of this oxidation process were examined computationally, and the results are discussed later in this study.

**TABLE 3:** Synthesis of polysubstituted indene‐2,2‐dicarbonitrile derivatives by aldehydes.^a^, ^b^

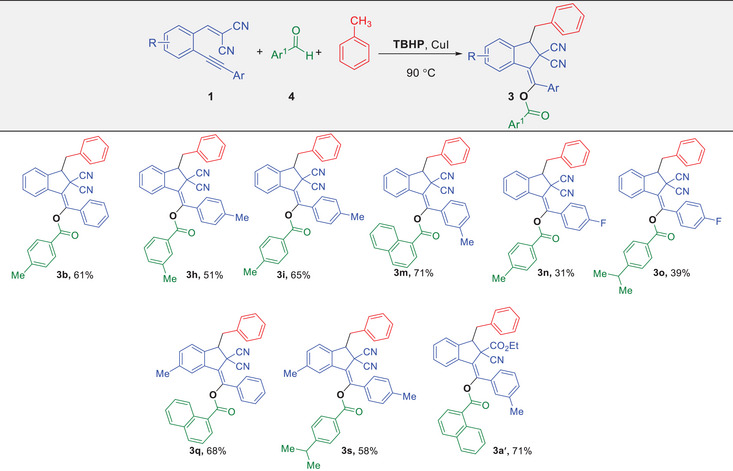

^a^ Reaction conditions: 1,5‐enynes 1 (1.0 equiv.); aldehydes 4 (2.4 equiv.), TBHP (5.0 equiv.), and CuI (20 mol%) in 0.5 mL of PhCH_3_ at 90°C for 8 h

^b^ Isolated yields.

To evaluate the reactivity of aldehydes, a range of aromatic derivatives was examined, all of which afforded the corresponding indene products **3** in moderate to good yields (Table 3). We found that aldehydes bearing electron‐donating substituents such as 4‐Me, 4‐i‐Pr, and 1‐naphthyl groups underwent the reaction smoothly, affording the expected products (**3b**, **3** **h**, **3i**, **3m**, **3s**, **3q**, **3a′**). In contrast, aldehydes bearing electron‐withdrawing substituents such as CF_3_ or halogens were incompatible with this transformation, and the corresponding products **3** were not detected.

**SCHEME 2:**
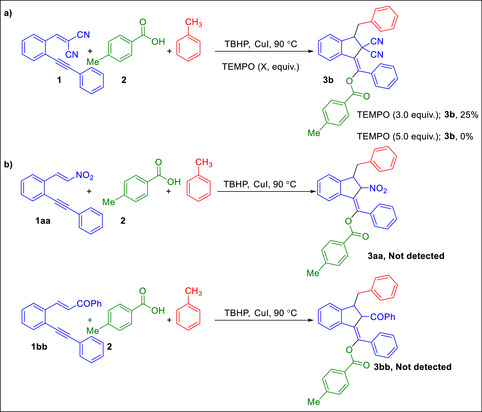
Control experiments.

To gain deep insight into the reaction mechanism, we set out to conduct radical inhibition experiments using radical scavenger (TEMPO) under the standard reaction conditions lead to inhibiting reaction (Scheme 2a). These results indicated that a radical pathway might be involved in this transformation. Furthermore, when the reaction was performed with 1,5‐enyenes **1aa** and **1bb** bearing only a single strongly π‐accepting substituent (such as NO_2_, COPh) under optimized conditions, the expected products **3aa** and **3bb** were not detected (Scheme 2b). These findings clearly demonstrate that in this transformation, the presence of two bear strongly π‐accepting substituents (such as CN or CO_2_Et) is essential.

### 2.1 DFT Mechanistic Studies—Questions Prompted by Our Experiments

Although the experimental optimization established efficient conditions for the radical cascade cyclization, several mechanistic questions remain unresolved. The first concerns the role of toluene: in our earlier study [60] under similar Cu/TBHP conditions, toluene served only as solvent and was never incorporated into the catalytic cycle, whereas in the present system it clearly acts as a reactant and becomes incorporated into the final product. Why does toluene behave differently under these conditions? A second question relates to the HAT step required to generate the benzyl radical. Which species within the Cu/TBHP manifold initiates this crucial process? A further issue relates to regioselectivity: once formed, why does the benzyl radical add exclusively to a single site of the 1,5‐enyne substrate, even though several radical acceptor sites are available? The role of carboxylic acid in the transformation remains an important question. What function does it serve in the reaction, and why does the absence of acid completely shut down the cascade cyclization? The mode of benzoates incorporation remains unclear. How is the benzoate moiety introduced into a possible radical intermediate, and why does this addition occur with complete regioselectivity at a single site of the substrate? As discussed above, when benzoic acids are replaced with aldehydes, the same indene products are obtained. This observation implies that the aldehydes must be oxidized in situ to the corresponding acids under the reaction conditions. How does this oxidation occur, and through which mechanism? To gain insight into these mechanistic issues, density functional theory (DFT) calculations were performed at the SMD/B3LYP‐D3/def2‐TZVP//SMD/B3LYP‐D3/def2‐SVP level of theory in toluene. This computational setup allowed us to evaluate the energetics of each elementary step and clarify the mechanistic details of this crucial transformation.

### 2.2 DFT Mechanistic Studies—Role of Benzoic Acid in Trapping Cu(II) and Initiating HAT by *t*BuO^•^

**SCHEME 3:**
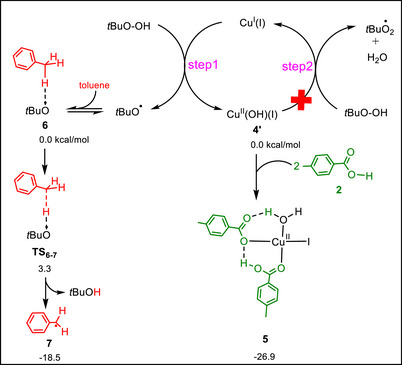
DFT mechanistic studies showing the CuI/TBHP redox cycle, trapping of Cu(II)(OH)I by benzoic acid to form complex **5** and thereby shutting down step 2, and hydrogen atom transfer (HAT) from toluene by *t*BuO^•^ to yield the benzyl radical **7**. The relative free energies are calculated at the SMD/B3LYP‐D3/def2‐TZVP//SMD/B3LYP‐D3/def2‐SVP level of theory in toluene.

Based on the widely accepted mechanism for catalytic reactions involving CuI and TBHP, the transformation begins with the reaction of the [CuI] complex with *t*BuOOH, producing the *t*BuO^•^ radical together with the Cu^II^(OH)I species (step 1, Scheme 3). The Cu^II^(OH)I intermediate then reacts with another equivalent of *t*BuOOH to generate the *t*BuOO^•^ radical, while Cu(II) is reduced back to Cu(I), thereby completing the redox cycle (step 2, Scheme 3) [61, 62, 63].

In a recent study we proposed that in the presence of benzoic acids, the Cu(II)(OH)I complex is trapped by protonation, thereby preventing its further reaction with a second equivalent of *t*BuOOH and effectively halting step 2 of the redox cycle [60]. In the present work, this hypothesis is computationally supported by examining the reactivity of cis‐ and trans‐Cu(II)(OH)I with one and two equivalents of 4‐methylbenzoic acid. As shown in Scheme 3, the most stable species is calculated to be complex **5**, formed from the reaction of cis‐Cu(II)(OH)I with two equivalents of acid, with an exergonicity of −26.9 kcal mol^−^^1^. The relative stabilities of other possible complexes are provided in the Supporting Information (Figure S1). Our calculations further indicate that the OH ligand in Cu(II)(OH)I is highly basic and readily abstracts a proton from the acid to yield a coordinated H_2_O ligand in complex **5**. This behavior explains why step 2 of the redox cycle is shut down: conversion of OH into H_2_O removes the basic functionality required for further activation of *t*BuOOH and traps Cu(II) predominantly as complex **5**. This conclusion is consistent with our recent study, which demonstrated that for *t*BuOOH to be converted into a *t*BuOO^•^ radical, the metal complex must possess a basic ligand capable of deprotonating *t*BuOOH [64]. Conversion of the OH ligand into a coordinated H_2_O ligand eliminates this requirement and thus leaves Cu(II) available to mediate the final step of the reaction (vide infra).

As shown in Scheme 3, step 1 of the redox cycle generates the *t*BuO^•^ radical. Because this radical is formed in the immediate presence of toluene, the bulk solvent, it is readily available to undergo a HAT process to produce the benzyl radical. This HAT step is calculated to proceed with an activation barrier of only 3.3 kcal mol^−^^1^ and is highly exergonic by about −18.5 kcal mol^−^^1^. The combination of this very low barrier and strong exergonicity indicates that *t*BuO^•^ has essentially no kinetic opportunity to engage in alternative reactions; instead, it rapidly abstracts a benzylic hydrogen from toluene, yielding the benzyl radical as the primary reactive species.

### 2.3 DFT Mechanistic Studies—Competition Between Benzyl Radical Addition and Deactivation

**SCHEME 4:**
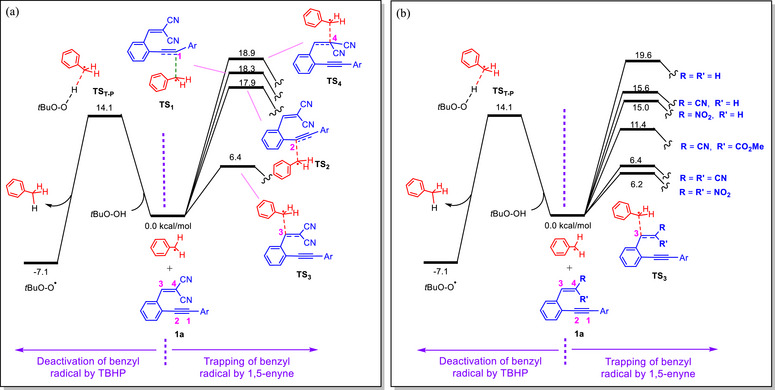
Free Energy profiles comparing benzyl radical deactivation by *t*BuOOH with benzyl radical addition: (a) to enyne **1a** at four possible sites; (b) at site 3 of enynes with different substituents R and R′. Relative free energies (kcal mol^−^^1^) were calculated at the SMD/B3LYP‐D3/def2‐TZVP//SMD/B3LYP‐D3/def2‐SVP level of theory in toluene.

As shown in Scheme 4a, once the benzyl radical is generated, two possible pathways may be considered. In the first scenario, the benzyl radical reacts with *t*BuOOH to produce the more stable *t*BuOO^•^ radical. This process is exergonic by −7.1 kcal mol^−^^1^ and occurs with an activation free energy of 14.1 kcal mol^−^^1^ through **TS_T‐P_**. In the second scenario, the benzyl radical can undergo addition to the 1,5‐enyne substrate; in our calculations, compound **1a** was used as the model enyne. The enyne offers four different possible addition sites (sites 1–4). Our calculations show that addition at site 3 via transition structure **TS_3_** is the most favorable pathway, with a barrier of only 6.4 kcal mol^−^^1^, whereas additions at the other sites are much less accessible, requiring barriers above 17 kcal mol^−^^1^. Comparison of these two scenarios clearly reveals that **TS_3_** lies lower in energy than **TS_T–P_** by about 7.7 kcal mol^−^^1^. Therefore, under the reaction conditions, the benzyl radical preferentially adds to the enyne rather than abstracting a hydrogen atom from *t*BuOOH.

As shown in Scheme 4b, our calculations indicate that the reactivity of site 3 is strongly dependent on the substituents attached to the terminal carbon of the alkene moiety in the enyne (R and R'). For **TS_3_** to lie below **TS_T‐P_**, both R and R' must be π‐accepting groups such as CN, NO_2_ or CO_2_Me. If only one of R or R' is π‐accepting, **TS_3_** shifts above **TS_T‐P_**, and in this case the benzyl radical does not add to the enyne but is instead deactivated by *t*BuOOH. These computational results clearly explain why only the enynes reported in Table 2 are reactive, since in all of them both R and R' are π‐accepting substituents. These results also clarify why in our previous studies, although toluene was present as the solvent under Cu/TBHP conditions, it never acted as a reactant: the enynes previously employed lacked electron‐withdrawing substituents at both R and R'. In support of this conclusion, we synthesized two enynes bearing R = NO_2_, R' = H, and R = COPh, R' = H, and found that the desired product **3aa** and **3bb** were not formed under the standard reaction conditions (Scheme 2b).

The enhanced reactivity of enynes bearing electron‐withdrawing R and R' groups can be attributed to their reduced singlet–triplet energy gap, as supported by our calculations (Table S2), which facilitates interaction with the benzyl radical and lowers the barrier for addition.

### 2.4 DFT Mechanistic Studies—Cyclization Pathways and Formation of Indene **3b**

**SCHEME 5:**
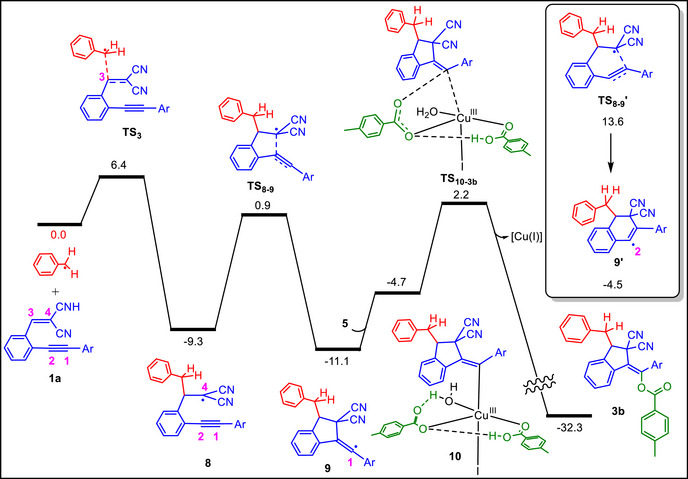
Free energy profile for the formation of indene **3b** calculated at the SMD/B3LYP‐D3/def2‐TZVP//SMD/B3LYP‐D3/def2‐SVP level of theory in toluene. Relative free energies are given in kcal mol^−^^1^.

The full energy profile for the formation of indene **3b** is presented in Scheme 5, beginning from the stage at which the benzyl radical is generated. Following its addition to the enyne through **TS_3_**, intermediate **8** is formed, in which the unpaired electron is mainly localised at C4. From this intermediate, two possible cyclization routes can be considered. In the first pathway, C4 adds to C2 to generate a five‐membered ring in intermediate **9** via transition structure **TS_8‐9_**. In the second pathway, C4 adds to C1 to generate a six‐membered ring in intermediate **9'** via transition structure **TS_8‐9'_**, depicted in the boxed structures in Scheme 5.

According to our calculations, **TS_8‐9_** lies 12.7 kcal mol^−^^1^ lower in energy than **TS_8‐9'_**, indicating that formation of the five‐membered ring is energetically more favorable than formation of the six‐membered ring. This result is consistent with experiment, where cyclization furnishes only the five‐membered indene ring and never the six‐membered one. The greater favorability of five‐membered ring formation compared to the six‐membered analogue can be plausibly explained by the relative stability of the resulting intermediates: intermediate **9** is calculated to be 6.6 kcal mol^−^^1^ lower in energy than **9'**. Intermediate **9** is more stable than **9'** because in **9** the single electron is located on C1, where it is further stabilized by π‐conjugation with the adjacent phenyl ring. In contrast, in **9'** the unpaired electron is located on C2, where its orientation is orthogonal to the π‐system of the neighboring aromatic ring and therefore cannot gain additional stabilization through π‐conjugation.

Once intermediate **9** is formed, it can react with the Cu(II) complex **5**, which is trapped by two molecules of 4‐methylbenzoic acid as discussed above, to generate the Cu(III) complex **10**. From this intermediate, C─OAc reductive elimination occurs via transition structure **TS_10‐3b_** to afford the final product **3b**, while simultaneously regenerating the Cu(I) catalyst.

### 2.5 Summary of the DFT‐Based Proposed Mechanism

**SCHEME 6:**
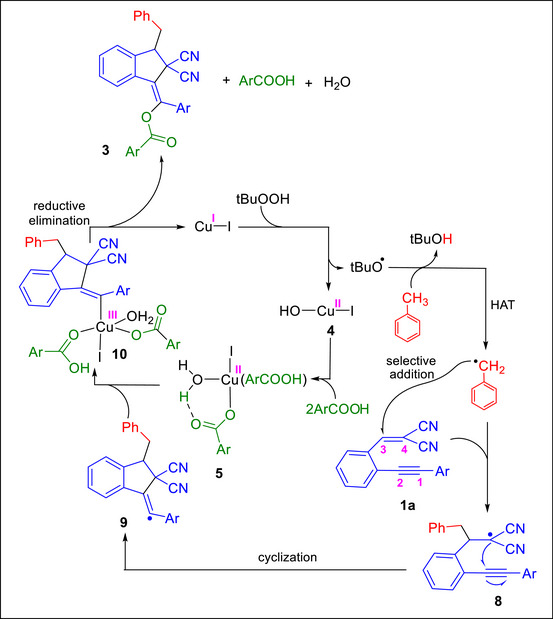
DFT‐based proposed catalytic cycle for the Cu/TBHP‐mediated three‐component cascade cyclization of 1,5‐enynes with toluene and benzoic acids.

Scheme 6 summarizes the DFT‐based proposed overall reaction pathway, in which toluene (solvent) undergoes C─H activation to generate a benzyl radical that engages in a cascade sequence involving an initial Giese‐type radical addition to 1,5‐enynes, cyclization, and copper‐mediated functionalization to furnish indene **3b**. The reaction begins with oxidation of Cu(I) in CuI to Cu(II) in Cu(OH)I by *t*BuOOH, generating the *t*BuO^•^ radical. The *t*BuO^•^ radical immediately promotes a HAT process, abstracting a hydrogen atom from toluene to give the benzyl radical. A crucial role of the benzoic acid additive is to trap the Cu(II) species by protonating the OH ligand in Cu(OH)(I), thereby preventing its reduction back to Cu(I) by another equivalent of *t*BuOOH and maintaining Cu(II) in the cycle to enable subsequent benzoate functionalization. The benzyl radical then adds preferentially to the alkene moiety of the 1,5‐enyne when it bears two geminal electron‐withdrawing substituents such as CN; otherwise, it is deactivated through hydrogen abstraction from *t*BuOOH. The regioselective addition of the benzyl radical to the enyne is thus dictated by the presence of these two electron‐withdrawing substituents, which lower the activation barrier for addition at site 3 of enyne **1a** relative to all other possible sites. Following addition of the benzyl radical at site 3, intramolecular cyclization occurs to produce radical intermediate **9**, in which the unpaired electron is localized at the C1 atom. Coordination of intermediate **9** through its C1 atom to the copper center of complex **5** oxidizes the metal to Cu(III), setting the stage for a facile C─O reductive elimination that furnishes the indene product **3** and simultaneously regenerates the Cu(I) catalyst.

### 2.6 DFT Studies on Aldehyde Oxidation Mechanism

**SCHEME 7:**
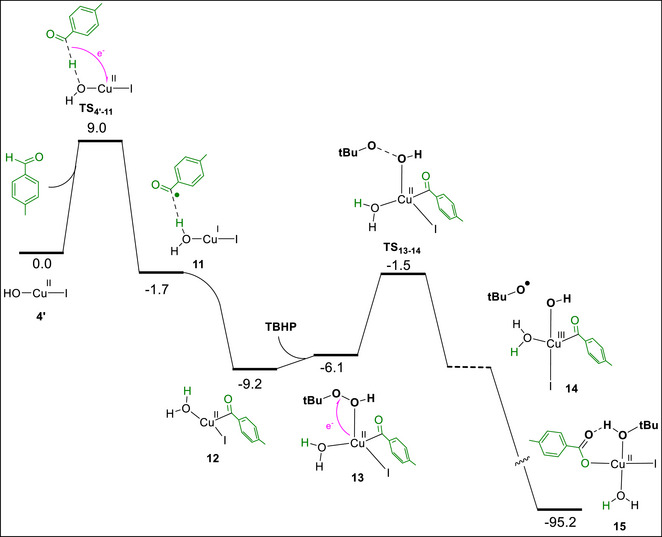
Computed free‐energy profile for the oxidation of a representative aldehyde to the corresponding benzoic acid under Cu/TBHP conditions. Relative free energies (kcal mol^−^^1^) were calculated at the SMD/B3LYP‐D3/def2‐TZVP//SMD/B3LYP‐D3/def2‐SVP level of theory in toluene.

As discussed above, replacing benzoic acids with aldehydes under the standard reaction conditions affords the same indene products, indicating that the aldehydes must be oxidized to the corresponding acids under the utilized catalytic system. To clarify how this oxidation occurs, we examined the mechanism computationally and found that both Cu(OH)I (**4′**) and TBHP cooperate to promote this oxidation. The calculated free energy profile (Scheme 7) reveals that the reaction begins with abstraction of the formyl hydrogen atom of the aldehyde by the hydroxo ligand of complex **4′**, accompanied by a single‐electron transfer from the aldehyde to the Cu(II) center. This redox process generates intermediate **11**, in which an acyl radical is formed as an outer‐sphere ligand. Our calculations indicate that this radical can approach the copper center barrierlessly, reoxidizing it to Cu(II) and affording the more stable complex **12**.

Subsequent interaction of complex **12** with TBHP produces the four‐coordinate complex **13**, which is highly predisposed to an internal redox process wherein the Cu(II) center transfers one electron to the O─O bond of the coordinated TBHP, generating the Cu(III) complex **14** that bears a tert‐butoxy radical as an outer‐sphere ligand. However, complex **14** was detected only as a transient geometry along the forward IRC path and was not found to be a true minimum on the potential energy surface. The calculations show that this geometry directly evolves to complex **15**. In fact, the Cu(III) complex **14** is highly reactive and barrierlessly undergoes C─O reductive elimination to furnish the carboxylic acid product, regenerating the Cu(I) species. The outer‐sphere tert‐butoxy radical ligand in **14** then immediately interacts with Cu(I), reoxidizing it to Cu(II) while being converted into a tert‐butoxy anion. This strongly basic anion subsequently abstracts a proton from the benzoic acid formed in situ, affording the stable complex **15**.

Formation of complex **15** from the reaction between complex **4′**, the aldehyde, and TBHP is calculated to be highly exergonic (Δ*G* ≈ −95 kcal mol^−^^1^). Complex **15** closely resembles complex **5**, which forms when benzoic acid is used as the substrate. Consequently, when aldehydes are employed instead of acids, complex **15** functions analogously to complex **5**, enabling reaction with radical **9** (Scheme 6) to generate the corresponding Cu(III) species. Subsequent C─O reductive elimination from this Cu(III) intermediate furnishes the final indene product.

## 3 Conclusions

In conclusion, we have developed the first methodology that achieves direct C─H bond functionalization of toluene solvent through benzyl radical generation and its regioselective addition to multi‐π systems. This strategy enables a three‐component cascade reaction of 1,5‐enynes, toluene solvent, and benzoic acids in the presence of a Cu(I) catalyst and TBHP oxidant, affording polysubstituted indene derivatives of broad significance in catalysis, materials, and medicinal chemistry. Interestingly, replacing benzoic acids with aldehydes affords the same indene products, indicating that aldehydes are oxidized to the corresponding acids under the reaction conditions. Our DFT calculations further show that this oxidation is mediated cooperatively by Cu(OH)I and TBHP.

The transformation developed in this study exploits toluene in a dual role as both solvent and benzyl radical source, highlighting a sustainable approach to C─H functionalization. Our investigations provided detailed mechanistic insight, clarifying how the Cu(I)/TBHP manifold generates the *t*BuO^•^ radical, which then abstracts a benzylic hydrogen atom to form the benzyl radical. The calculations further revealed that benzoic acids play a dual role. First, they protonate the Cu(II)─OH intermediate generated during benzyl radical formation, thereby stabilizing Cu(II) as the key intermediate needed for continuation of the catalytic cycle. Second, they supply the benzoate group that is incorporated into the final product through C─O reductive elimination from a Cu(III) intermediate. Importantly, our investigations uncovered a key design principle: regioselective addition of the benzyl radical occurs only when both terminal substituents of the alkene moiety in the 1,5‐enyne are strongly π‐accepting, which lowers the activation barrier for benzyl radical addition below that of competing deactivation pathways. This requirement is confirmed by the substrate scope established in our experiments and is further supported by our DFT results.

More broadly, this work establishes a general framework for harnessing the inert solvent toluene as a reactive carbon source by enabling its controlled installation onto multi‐π systems under catalytic conditions.

## Conflicts of Interest

The authors declare no conflicts of interest.

## Supporting information



Additional references cited within the Supporting Information [65, 66, 67, 68, 69, 70, 71, 72, 73]. **Supporting File 1**: chem70721‐sup‐0001‐SuppMat.docx

## Data Availability

The data that support the findings of this study are available in the supplementary material of this article.
