# Supplementary material for: Harnessing Toluene Solvent as a Reactant: Regioselective Benzyl Radical Addition to Multi‐π 1,5‐Enynes in a Copper‐Catalyzed Cascade to Indenes
Source: Chemistry. 2026 Jan 28;32(14):e03120. doi: 10.1002/chem.202503120 (PMC13088009; doi:10.1002/chem.202503120)
Supplement: Supplementary file 1 — Additional references cited within the Supporting Information [65, 66, 67, 68, 69, 70, 71, 72, 73]. Supporting File 1: chem70721‐sup‐0001‐SuppMat.docx [file CHEM-32-e03120-s001.docx]

**Supplementary Information for:**

**Harnessing Toluene Solvent as a Reactant: Regioselective Benzyl Radical Addition to Multi-π 1,5-Enynes in a Copper-Catalyzed Cascade to Indenes**

Saideh Rajai-Daryasarei,^a^ Fatemeh Chahardehi,^b^ Mohammad Mahdi Sharifani,^b^ Morteza Jamshidi,^c^ Robert Stranger,^c^ Frank Rominger,^d^ Alireza Ariafard,*^c^ Saeed Balalaie*^b^

*^a^School of Chemistry, College of Science, University of Tehran, 14155-6455 Tehran, Iran.*

*^b^Peptide Chemistry Research Institute, K. N. Toosi University of Technology, P.O. Box 15875-4416, Tehran, Iran E-mail:* *[balalaie@kntu.ac.ir](mailto:balalaie@kntu.ac.ir).*

*^c^Research School of Chemistry, Australian National University, Canberra, ACT 2601, Australia E-mail:* [*alireza.ariafard@anu.edu.au*](mailto:alireza.ariafard@anu.edu.au)*.*

*^d^Organisch-Chemisches Institut der Universität Heidelberg, Im Neuenheimer Feld 271, 69120 Heidelberg, Germany.*

**Table of Contents:**

**1. General information**

**2. General procedures for the preparation of starting materials**

**3. General experimental procedures**

**4. Experimental characterization data**

**5. ^1^H, ^13^C NMR and ^19^F NMR spectra of products**

**6. Crystal data of 3b**

**7.Computational Data**

**8. References**

**1. General information**

All chemicals were purchased from Merck (Germany) and were used without further purification. Melting points were measured on an Electrothermal 9100 apparatus. High-resolution mass spectrum (HRMS) (ESI-TOF) was recorded using a Waters LCT Premier ^XE^ mass spectrometer. X-ray crystal data were measured on a Bruker APEX-II Quazar area detector. Nuclear Magnetic Resonance (NMR) spectra (^1^H and ^13^C NMR) were recorded on a Bruker AVANCE 300, 400, and 500 MHz spectrometer. Column chromatographies were done using silica gel 230–240 meshes.

**2. General procedures for the preparation of starting materials**

**2. 1. General procedure for preparation of** **2-(2-(arylethynyl)benzylidene)malononitrile.^1^**

**Step 1:** [2-bromobenzaldehyde](https://www.google.com/search?sca_esv=618eb12c7c97926b&rlz=1C1CHZN_enIR1011IR1011&sxsrf=AHTn8zplI9GzDAsPuhj7-_0PTcbWymPuUw:1743180583023&q=2-bromobenzaldehyde&spell=1&sa=X&ved=2ahUKEwiRtrjbna2MAxU-gf0HHVx4BVEQkeECKAB6BAgNEAE) **A** (20 mmol), and acetylene **B** (24 mmol) were added to a mixture of CuI (0.2 mmol) and PdCl_2_(PPh_3_)_2_ (0.4 mmol) in Et_3_N (50 mL). The resulting mixture was heated under an argon atmosphere in an oil bath at 50 °C for 12 hours. After the reaction was completed, the mixture was quenched by adding distilled water and extracted with DCM (three times). The combined organic layers were washed with brine, dried over Na_2_SO_4_, filtrated, and concentrated under reduced pressure. The residue was purified by column chromatography on silica gel, eluting with n-hexane and ethyl acetate (50:1) to afford the desired product **C**.

**Step 2:** To a stirred solution of 2-alkynylaldehyde **D** (10.0 mmol) and malononitrile/ alkyl 2-cyanoacetate **C** (12 mmol) in 15 mL of ethanol was added piperidine (5% mol). Then, the resulting mixture was allowed to stirre for 2 h. Next, the solid product was filtered and washed repeatedly with cold ethanol. Finally, recrystallization by ethanol afforded the desired products **1**.

**3. General experimental procedures**

**3.1. General procedure for the preparation (*E*)-(3-benzyl-2,2-dicyano-2,3-dihydro-1*H*-inden-1-ylidene)(aryl)methyl benzoate**, **exemplified with 3b**:

A mixture of 2-(2-(phenylethynyl)benzylidene)malononitrile **1** (0.05 mmol, 0.013 g), *p*-methylbenzoic acid **2** (0.12 mmol, 0.014 g), TBHP (5.0 equiv., 0.25 mmol) and CuI (0.01 mmol, 0.002 g) in toluene (0.5 mL) was stirred at 90 °C for 4 h. The completion of the reaction was indicated by TLC monitoring, the reaction mixture was cooled to ambient temperature. A saturated solution of NaHCO_3_ (3 mL) was added and the product was extracted with ethyl acetate (3 × 4 mL). The combined organic phase was dried over Na_2_SO_4_. The solvent was removed under the reduced pressure. The residue was purified by column chromatography using *n*-hexane-EtOAc (15:1) to afford the desired product **3b (**35 mg, Yield 73%).

**3.2. General procedure for the preparation (*E*)-(3-benzyl-2,2-dicyano-2,3-dihydro-1*H*-inden-1-ylidene)(aryl)methyl benzoate by benzaldehydes**, **exemplified with 3b**:

A mixture of 2-(2-(phenylethynyl)benzylidene)malononitrile **1** (0.05 mmol, 0.013 g), *p*-methylbenzaldehyde **2** (0.12 mmol, 0.014 g), TBHP (5.0 equiv., 0.25 mmol) and CuI (0.01 mmol, 0.002 g) in toluene (0.5 mL) was stirred at 90 °C for 8 h. The completion of the reaction was indicated by TLC monitoring, the reaction mixture was cooled to ambient temperature. A saturated solution of NaHCO_3_ (3 mL) was added and the product was extracted with ethyl acetate (3 × 4 mL). The combined organic phase was dried over Na_2_SO_4_. The solvent was removed under the reduced pressure. The residue was purified by column chromatography using *n*-hexane-EtOAc (15:1) to afford the desired product **3b (**Yield 61%).

**4. Experimental characterization data**

**(*E*)-(3-Benzyl-2,2-dicyano-2,3-dihydro-1*H*-inden-1-ylidene)(phenyl)methyl 3-methylbenzoate (3a):** Yield: 34 mg, 71%; brown oil. ^1^H NMR (400 MHz, CDCl_3_): δ = 2.47 (s, 3H), 3.29 (dd, *J* = 14.0, 8.0 Hz, 1H), 3.44 (dd, *J* = 14.0, 7.0 Hz, 1H), 4.24 (t, *J* = 7.7 Hz, 1H), 6.86-6.94 (m, 1H), 7.20-7.26 (m, 2H), 7.26-7.31 (m, 2H), 7.31-7.39 (m, 3H), 7.44 (t, *J* = 8.0 Hz, 1H), 7.49-7.57 (m, 4H), 7.58-7.63 (m, 1H), 7.73-7.78 (m, 2H), 7.92-8.06 (m, 2H). ^13^C NMR (100 MHz, CDCl_3_): δ = 21.3, 37.8, 43.7, 56.2, 113.5, 113.8, 124.1, 124.8, 125.3, 127.4, 127.6, 128.4, 128.8, 128.9, 129.0, 129.3, 129.5, 129.7, 130.4, 130.8, 130.9, 133.7, 134.5, 135.1, 136.1, 138.9, 141.1, 147.5, 164.1. HRMS-ESI (m/z): calc. for C_33_H_25_N_2_O_2_ [M+H]^+^ 481.1910, found 481.1907.

**(*E*)-(3-Benzyl-2,2-dicyano-2,3-dihydro-1*H*-inden-1-ylidene)(phenyl)methyl 4-methylbenzoate (3b):** Yield: 35 mg, 73%; white solid; m.p. 154-156 °C. ^1^H NMR(400 MHz, CDCl_3_): δ = 2.51 (s, 3H), 3.30 (dd, *J* = 14.0, 8.0 Hz, 1H), 3.45 (dd, *J* = 14.0, 7.0 Hz, 1H), 4.25 (t, *J* = 8.0 Hz, 1H), 6.91-6.94 (m, 1H), 7.22-7.26 (m, 2H), 7.28-7.31 (m, 2H), 7.32-7.35 (m, 1H), 7.35-7.39 (m, 4H), 7.51-7.55 (m, 3H), 7.61- 7.64 (m, 1H), 7.74-7.79 (m, 2H), 8.10 (d, *J* = 8.3 Hz, 2H). ^13^C NMR (100 MHz, CDCl_3_): δ = 21.9, 37.8, 43.7, 56.2, 113.5, 113.9, 124.1, 124.8, 125.3, 125.8, 127.4, 128.8, 128.9, 129.1, 129.3, 129.5, 129.6, 129.7, 130.5, 130.8, 133.8, 134.5, 136.1, 141.1, 145.4, 147.6, 164.1. HRMS-ESI (m/z): calc. for C_33_H_25_N_2_O_2_ [M+H]^+^ 481.1910, found 481.1911.

**(*E*)-(3-Benzyl-2,2-dicyano-2,3-dihydro-1*H*-inden-1-ylidene)(phenyl)methyl 2-chlorobenzoate (3c):** Yield: 29 mg, 58%; white solid; m.p. 228-230 °C. ^1^H NMR (300 MHz, CDCl_3_): δ = 3.27 (dd, *J* = 13.9, 8.4 Hz, 1H), 3.43 (dd, *J* = 13.9, 7.0 Hz, 1H), 4.23 (t, *J* = 7.7 Hz, 1H), 6.84-6.95 (m, 1H), 7.18-7.25 (m, 2H), 7.25-7.30 (m, 2H), 7.29-7.38 (m, 3H), 7.47 - 7.56 (m, 4H), 7.56-7.64 (m, 2H), 7.68 (t, *J* = 7.4 Hz, 1H), 7.71-7.82 (m, 2H), 8.18 (d, *J* = 7.0 Hz, 1H). ^13^C NMR (75 MHz, CDCl_3_): δ = 37.8, 43.7, 56.2, 113.5, 113.7, 124.8, 125.3, 127.0, 127.4, 128.8, 128.9, 129.0, 129.1, 129.2, 129.3, 129.5, 129.9, 130.9, 131.9, 132.4, 133.4, 134.1, 134.3, 135.2, 136.1, 141.3, 147.1, 162.5. HRMS-ESI (m/z): calc. for C_32_H_22_ClN_2_O_2_ [M+H]^+^ 501.1364, found 501.1360.

**(*E*)-(3-Benzyl-2,2-dicyano-2,3-dihydro-1*H*-inden-1-ylidene)(phenyl)methyl 2-bromoobenzoate (3d):** Yield: 33 mg, 61%; red oil. ^1^H NMR (400 MHz, CDCl_3_): δ = 3.23 (dd, *J* = 14.0, 8.5 Hz, 1H), 3.38 (dd, *J* = 14.0, 7.0 Hz, 1H), 4.19 (t, *J* = 7.7 Hz, 1H), 6.86 (d, *J* = 7.7 Hz, 1H), 7.16-7.22 (m, 2H), 7.23-7.24 (m, 2H), 7.25-7.27 (m, 1H), 7.27-7.35 (m, 2H), 7.38- 7.43 (m, 2H), 7.45-7.51 (m, 3H), 7.58 (d, *J* = 7.3 Hz, 1H), 7.67-7.80 (m, 3H), 7.96-8.04 (m, 1H). ^13^C NMR (100 MHz, CDCl_3_): δ = 37.8, 43.7, 56.2, 113.4, 113.7, 123.1, 124.8, 125.4, 127.4, 127.6, 128.8, 128.9, 129.1, 129.2, 129.3, 129.5, 129.6, 129.8, 130.9, 132.4, 133.3, 134.0, 134.2, 135.3, 136.1, 141.3, 147.1, 162.8. HRMS-ESI (m/z): calc. for C_32_H_22_BrN_2_O_2_ [M+H]^+^ 545.0858, found 545.0861.

**(*E*)-(3-Benzyl-2,2-dicyano-2,3-dihydro-1*H*-inden-1-ylidene)(phenyl)methyl 2- iodobenzoate (3e):** Yield: 37 mg, 62%; white solid; m.p. 164-166 °C. ^1^H NMR (400 MHz, CDCl_3_): δ = 3.20 (dd, *J* = 14.0, 8.3 Hz, 1H), 3.34 (dd, *J* = 14.0, 7.1 Hz, 1H), 4.15 (t, *J* = 7.8 Hz, 1H), 6.82 (d, *J* = 7.3 Hz, 1H), 7.13-7.16 (m, 1H), 7.17-7.20 (m, 3H), 7.20-7.24 (m, 2H), 7.24-7.28 (m, 2H), 7.37-7.48 (m, 4H), 7.51 (d, *J* = 7.0 Hz, 1H), 7.65-7.74 (m, 2H), 8.00 (dd, *J* = 7.9, 1.7 Hz, 1H), 8.04 (dd, *J* = 7.9, 1.3 Hz, 1H).^13^C NMR (100 MHz, CDCl_3_): δ = 37.8, 43.8, 56.2, 95.3, 113.4, 113.7, 124.8, 125.4, 127.4, 128.2, 128.8, 128.9, 129.1, 129.2, 129.3, 129.4, 129.5, 129.8, 130.9, 132.0, 133.3, 133.9, 134.3, 136.1, 141.4, 142.3, 147.1, 163.2. HRMS-ESI (m/z): calc. for C_32_H_22_IN_2_O_2_ [M+H]^+^ 593.0720, found 593.0718.

**(*E*)-(3-Benzyl-2,2-dicyano-2,3-dihydro-1*H*-inden-1-ylidene)(phenyl)methyl 2-(trifluoromethyl)benzoate (3f):** Yield: 29 mg, 54%; white solid; m.p. 190-192 °C. ^1^H NMR (300 MHz, CDCl_3_): δ = 3.27 (dd, *J* = 13.9, 8.4 Hz, 1H), 3.43 (dd, *J* = 13.9, 7.1 Hz, 1H), 4.23 (t, *J* = 7.7 Hz, 1H), 6.85-6.97 (m, 1H), 7.18-7.29 (m, 4H), 7.29-7.39 (m, 3H), 7.47-7.59 (m, 4H), 7.67- 7.76 (m, 2H), 7.80 (d, *J* = 8.4 Hz, 2H), 8.29 (d, *J* = 8.4 Hz, 2H). ^13^C NMR (125 MHz, CDCl_3_) δ = 37.9, 43.7, 56.3, 113.4, 113.7, 123.5 (q, *J* = 271 Hz), 125.0, 125.1, 126.0, 126.1, 127.5, 128.9, 129.0, 129.1, 129.2, 129.3, 129.6, 130.0, 130.8, 131.1, 131.8, 133.3, 134.2, 136.0, 141.4, 147.1, 162.9. HRMS-ESI (m/z): calc. for C_33_H_22_F_3_N_2_O_2_ [M+H]^+^ 535.1628, found 535.1630.

**(*E*)-(3-Benzyl-2,2-dicyano-2,3-dihydro-1*H*-inden-1-ylidene)(phenyl)methyl 1*H*indole- 2-carboxylate (3g):** Yield: 31 mg, 61%; yellow oil. ^1^H NMR (400 MHz, CDCl_3_): δ = 3.24 (dd, *J* = 14.0, 8.3 Hz, 1H), 3.37 (dd, *J* = 14.0, 7.1 Hz, 1H), 4.18 (t, *J* = 7.7 Hz, 1H), 6.86 (d, *J* = 7.4 Hz, 1H), 7.18-7.22 (m, 2H), 7.22 -7.27 (m, 4H), 7.27-7.34 (m, 3H), 7.41-7.52 (m, 5H), 7.55 (d, *J* = 8.0 Hz, 1H), 7.70-7.76 (m, 2H), 8.06 (t, *J* = 9.0 Hz, 2H). ^13^C NMR (100 MHz, CDCl_3_): δ = 37.8, 43.7, 56.2, 95.4, 113.5, 113.7, 124.7, 124.8, 125.4, 127.4, 128.3, 128.9, 129.0, 129.1, 129.2, 129.4, 129.6, 129.9, 130.4, 131.0, 132.1, 132.3, 133.3, 134.1, 134.2, 136.1, 141.3, 142.4, 147.1, 163.2. HRMS-ESI (m/z): calc. for C_34_H_23_N_3_O_2_ [M+H]^+^ 506.1862, found 506.1870.

**(*E*)-(3-Benzyl-2,2-dicyano-2,3-dihydro-1*H*-inden-1-ylidene)(p-tolyl)methyl 3-methylbenzoate (3h)**: Yield: 34 mg, 69%; white solid; m.p. 216-218 °C. ^1^H NMR (400 MHz, CDCl_3_): δ = 2.43 (s, 3H), 2.48 (s, 3H), 3.32 (dd, *J* = 14.0, 8.3 Hz, 1H), 3.46 (dd, *J* = 14.0, 7.1 Hz, 1H), 4.26 (t, *J* = 7.7 Hz, 1H), 6.91-6.96 (m, 1H), 7.22-7.27 (m, 2H), 7.29-7.36 (m, 5H), 7.36-7.41 (m, 2H), 7.45 (t, *J* = 8 Hz, 1H), 7.53 (d, *J* = 7.4 Hz, 1H), 7.60-7.63 (m, 1H), 7.66 (d, *J* = 8.3 Hz, 2H), 7.99-8.04 (m, 2H). ^13^C NMR (100 MHz, CDCl_3_): δ = 21.4, 21.7, 37.8, 43.8, 56.3, 113.6, 114.0, 123.6, 124.7, 125.2, 127.4, 127.6, 128.6, 128.7, 128.8, 129.3, 129.5, 129.6, 129.8, 130.4, 130.9, 131.1, 132.5, 134.7, 135.0, 136.2, 138.9, 141.1, 147.8, 164.2. HRMS-ESI (m/z): calc. for C_34_H_27_N_2_O_2_ [M+H]^+^ 495.2066, found 495.2075.

**(*E*)-(3-benzyl-2,2-dicyano-2,3-dihydro-1*H*-inden-1-ylidene)(p-tolyl)methyl 4-methylbenzoate (3i):** Yield: 35 mg, 71%; white solid; m.p. 231-232 °C. ^1^H NMR (500 MHz, CDCl_3_)**:** δ = 2.40 (s, 3H), 2.48 (s, 3H), 3.28 (dd, *J* = 14.0, 8.4 Hz, 1H), 3.43 (dd, *J* = 14.0, 7.1 Hz, 1H), 4.23 (t, *J* = 7.9 Hz, 1H), 6.88-6.93 (m, 1H), 7.18-7.23 (m, 3H), 7.26-7.31 (m, 5H), 7.33-7.37 (m, 3H), 7.56-7.60 (m, 1H), 7.62 (d, *J* = 8.2 Hz, 2H), 8.06 (d, *J* = 8.2 Hz, 2H). ^13^C NMR (125 MHz, CDCl_3_) δ = 21.6, 21.8, 37.8, 43.8, 56.2, 113.5, 114.0, 124.7, 125.2, 126.0, 127.3, 128.2, 128.7, 128.8, 129.1, 129.4, 129.5, 129.6, 129.7, 130.4, 130.9, 134.7, 136.2, 141.0, 141.1, 145.2, 147.8, 164.0. HRMS-ESI (m/z): calc. for C_34_H_27_N_2_O_2_ [M+H]^+^ 495.2066, found 495.2070.

**(*E*)-(3-Benzyl-2,2-dicyano-2,3-dihydro-1*H*-inden-1-ylidene)(p-tolyl)methyl 2-iodobenzoate (3j):** Yield: 48 mg, 79%; white solid m.p. 196-198 °C. ^1^H NMR (300 MHz, CDCl_3_) δ = 2.4 (s, 3H), 3.28 (dd, *J* = 14.0, 8.4 Hz, 1H), 3.42 (dd, *J* = 14.0, 7.2 Hz, 1H), 4.23 (t, *J* = 7.5 Hz, 1H), 6.85-6.96 (m, 1H), 7.12-7.24 (m, 3H), 7.25-7.30 (m, 3H), 7.31-7.42 (m, 4H), 7.49-7.58 (m, 2H), 7.58-7.74 (m, 3H), 8.17 (d, *J* = 7.9 Hz, 1H). ^13^C NMR (125 MHz, CDCl_3_) δ = 21.7, 37.8, 43.9, 56.2, 95.4, 113.6, 113.8, 124.3, 124.8, 125.4, 127.4, 128.3, 128.9, 129.0, 129.3, 129.6, 129.7, 129.9, 130.5, 132.1, 132.5, 134.0, 134.4, 136.2, 141.2, 141.3, 142.4, 147.4, 163.3. HRMS-ESI (m/z): calc. for C_33_H_24_IN_2_O_2_ [M+H]^+^ 607.0876, found 607.0878.

**(*E*)-(3-Benzyl-2,2-dicyano-2,3-dihydro-1*H*-inden-1-ylidene)(p-tolyl)methyl 3-iodobenzoate (3k):** Yield: 43 mg, 71%; white solid; m.p. 206-208 °C. ^1^H NMR (400 MHz, CDCl_3_) δ = 2.45 (s, 3H), 3.32 (dd, *J* = 14.0, 8.2 Hz, 1H), 3.46 (dd, *J* = 14.0 , 7.1 Hz, 1H), 4.27 (t, *J* = 8.0 Hz, 1H), 6.94-6.98 (m, 1H), 7.23-7.33 (m, 5H), 7.34-7.44 (m, 5H), 7.57 (dd, *J* = 7.6, 2.0 Hz, 1H), 7.65 (d, *J* = 7.6 Hz, 2H), 8.05 (dt, 8.0, 1.2 Hz, 1H), 8.15-8.20 ( m, 1H), 8.51-8.57 (m, 1H). ^13^C NMR (100 MHz, CDCl_3_) δ = 21.7, 37.9, 43.8, 56.3, 94.3, 113.5, 113.9, 124.0, 124.9, 125.1, 127.5, 128.9, 129.0, 129.3, 129.5, 129.6, 129.8, 129.9, 130.5, 130.6, 134.3, 134.5, 136.1, 139.1, 141.3, 141.4, 143.1, 147.4, 162.6. HRMS-ESI (m/z): calc. for C_33_H_24_IN_2_O_2_ [M+H]^+^ 607.0876, found 607.0873.

**(*E*)-(3-Benzyl-2,2-dicyano-2,3-dihydro-1*H*-inden-1-ylidene)(p-tolyl)methyl 4-(trifluoromethyl)benzoate (3l):** Yield: 25 mg, 46%; white solid; m.p. 203–205 °C, ^1^H NMR (300 MHz, CDCl_3_): δ = 2.42 (s, 3H), 3.31 (dd, *J* = 14.0, 8.2 Hz, 1H), 3.45 (dd, *J* = 14.0, 7.3 Hz, 1H), 4.26 (t, *J* = 7.5 Hz, 1H), 6.90-7.01 (m, 1H), 7.21-7.32 (m, 5H), 7.33-7.42 (m, 4H), 7.51-7.59 (m, 1H), 7.65 (d, *J* = 8.1 Hz, 2H), 7.82 (d, *J* = 8.4 Hz, 2H), 8.32 (d, *J* = 8.3 Hz , 2H). ^13^C NMR (75 MHz, CDCl_3_) δ = 21.1, 37.4, 43.3, 55.8, 113.0, 113.4, 123.8, 124 (q, *J* = 272 Hz), 124.5, 125.5, 126.9, 128.4, 128.6, 128.8, 129.1, 129.3, 129.8, 130.0, 130.3, 131.5, 134.0, 135.4, 135.6, 138.6, 140.9, 141.0, 146.9, 162.4. ^19^F NMR (471 MHz, CDCl_3_) δ = -63.21. HRMS-ESI (m/z): calc. for C_34_H_24_F_3_N_2_O_2_ [M+H]^+^ 549.1784 found 549.1777.

**(*E*)-(3-Benzyl-2,2-dicyano-2,3-dihydro-1*H*-inden-1-ylidene)(m-tolyl)methyl 1-naphthoate (3m):** Yield: 45 mg, 85%; white solid; m.p. 171-173 °C. ^1^H NMR (400 MHz, CDCl_3_) δ = 2.49 (s, 3H), 3.32 (dd, *J* = 14.0, 8.2 Hz, 1H), 3.50 (dd, *J* = 14.0, 7.1 Hz, 1H), 4.30 (dd, *J* = 14.0, 3.4 Hz, 1H), 6.92-6.97 (m, 1H), 7.18-7.28 (m, 2H), 7.29-7.38 (m, 4H), 7.38-7.51 (m, 3H), 7.54-7.58 (m, 6H), 7.99 (d, *J* = 8.0 Hz, 1H), 8.21 (d, *J* = 8.0 Hz, 1H), 8.55-8.61 (m, 1H), 9.04 - 9.1 (m, 1H). ^13^C NMR (100 MHz, CDCl_3_) δ = 21.5, 37.9, 43.9, 56.3, 113.7, 114.0, 124.2, 124.5, 124.7, 124.8, 125.3, 125.7, 126.1, 126.8, 127.4, 128.5, 128.7, 128.9, 129.0, 129.3, 129.4, 129.6, 130.5, 131.7, 132.0, 132.2 133.8, 134.1, 134.7, 135.3, 136.2, 138.9, 141.2, 147.9, 164.3. HRMS-ESI (m/z): calc. for C_37_H_27_N_2_O_2_ [M+H]^+^ 531.2066, found 531.2073.

**(*E*)-(3-Benzyl-2,2-dicyano-2,3-dihydro-1*H*-inden-1-ylidene)(4-fluorophenyl)methyl 4-methylbenzoate (3n):** Yield: 23 mg, 46%; white solid m.p. 177-175 °C. ^1^H NMR (400 MHz, CDCl_3_) δ = 2.51 (s, 3H), 3.32 (dd, *J* = 13.9, 8.2 Hz, 1H), 3.45 (dd, *J* = 13.9, 7.2 Hz, 1H), 4.26 (t, *J* = 7.5 Hz, 1H), 6.99-6.92 (m, 1H), 7.26-7.19 (m, 3H), 7.26-7.32 (m, 3H), 7.32-7.40 (m, 5H), 7.65-7.59 (m, 1H), 7.80-7.72 (m, 2H), 8.09 (d, *J* = 8.3 Hz, 2H). ^13^C NMR (100 MHz, CDCl_3_) δ = 21.9, 37.8, 43.5, 56.2, 113.5, 113.8, 116.3 (d, *J* = 21.7 Hz), 124.6, 124.8, 125.3, 125.7, 127.5, 128.9, 129.3, 129.6, 129.7, 129.8, 129.9, 130.5, 131.2 (d, *J* = 9.2 Hz), 134.4, 136.0, 141.2, 145.5, 146.5, 163.9 (d, *J* = 250.2 Hz), 164.0. HRMS-ESI (m/z): calc. for C_33_H_24_FN_2_O_2_ [M+H]^+^ 499.1817, found 499.1825.

**(*E*)-(3-Benzyl-2,2-dicyano-2,3-dihydro-1*H*-inden-1-ylidene)(4-fluorophenyl)methyl 4-isopropylbenzoate (3o):** Yield: 29 mg, 55%; white solid m.p. 148-150 °C. ^1^H NMR (400 MHz, CDCl_3_) δ = 1.34 (d, *J* = 6.8 Hz, 6H), 3.05 (m, 1H), 3.31 (dd, *J* = 13.9, 8.2 Hz, 1H), 3.44 (dd, *J* = 13.9, 7.1 Hz, 1H), 4.26 (t, *J* = 7.4, 1H), 6.92 -6.97 (m, 1H), 7.20 (dd, *J* = 8.9, 8.4 Hz, 2H), 7.24-7.27 (m, 1H), 7.27-7.29 (m, 1H), 7.30-7.33 (m, 2H), 7.33-7.40 (m, 3H), 7.42 (d, *J* = 8.1 Hz, 2H), 7.61-7.66 (m, 1H), 7.71-7.78 (m, 2H), 8.11 (d, *J* = 8.6 Hz, 2H). ^13^C NMR (100 MHz, CDCl_3_) δ = 23.7, 34.5, 37.8, 43.5, 56.2,113.5, 113.8, 116.3 (d, *J* = 22.0 Hz), 124.7, 125.4, 126.0, 127.1, 127.5, 128.9, 129.3, 129.6, 129.8, 129.9, 130.6, 131.2, 131.3, 134.4, 136.0, 141.2, 146.6, 156.1, 163.9 (d, *J* = 250.7 Hz), 164.0. ^19^F NMR (471 MHz, CDCl_3_) δ = -108.22. HRMS-ESI (m/z): calc. for C_35_H_28_FN_2_O_2_ [M+H]^+^ 527.2130, found 527.2133.

**(*E*)-(3-Benzyl-2,2-dicyano-2,3-dihydro-1*H*-inden-1-ylidene)(4-cyanophenyl)methyl 2-iodobenzoate (3p):** Yield: 26 mg, 42% white solid m.p. 186-184 °C. ^1^H NMR (500 MHz, CDCl_3_) δ = 3.29-3.44 (m, 2H), 4.26 (t, *J =* 7.6 Hz, 1H), 6.95-7.04 (m, 1H), 7.24-7.31 (m, 3H), 7.32-7.39 (m, 5H), 7.53 (t, *J =* 7.6 Hz, 1H), 7.58-7.63 (m, 1H), 7.81 (d, *J* = 8.3 Hz, 2H), 7.88 (d, *J =* 8.3 Hz, 2H), 8.08 (d, *J =* 7.1 Hz, 1H), 8.14 (d, *J =* 7.8 Hz, 1H). ^13^C NMR (125 MHz, CDCl_3_) δ = 37.8, 43.2, 56.3, 95.4, 113.2, 113.4, 114.6, 118.0, 124.9 125.6, 126.6, 127.6, 128.4, 128.9, 129.5, 129.6, 130.0, 130.6, 131.8, 132.0, 132.8, 133.7, 134.3, 135.7, 137.6. 141.7, 142.6, 144.7, 163.0. HRMS-ESI (m/z): calc. for C_33_H_21_IN_3_O_2_ [M+H]^+^ 618.0673, found 618.0673.

**(*E*)-(3-Benzyl-2,2-dicyano-6-methyl-2,3-dihydro-1*H*-inden-1-ylidene)(phenyl)methyl 1-naphthoate (3q):** Yield: 41 mg, 77%; white solid m.p. 193-191 °C. ^1^H NMR (400 MHz, CDCl_3_) δ = 2.08 (s, 3H), 3.31 (dd, *J* = 13.9, 8.3 Hz, 1H), 3.47 (dd, *J* = 13.9, 7.1 Hz, 1H), 4.26 (t, *J* = 8.0 Hz, 1H), 6.82 (d, *J* = 8.0 Hz, 1H), 7.04 (d, *J* = 8.1 Hz, 1H), 7.28-7.34 (m, 3H), 7.34-7.42 ( m, 3H), 7.51-7.58 (m, 3H), 7.61-7.69 (m, 3H), 7.84-7.91 (m, 2H), 7.99 (d, *J* = 7.9 Hz, 1H), 8.20 (d, *J* = 7.9 Hz, 1H), 8.54 (d, *J* = 7.4 Hz, 1H), 8.98 (d, *J* = 8.4, 1H). ^13^C NMR (100 MHz, CDCl_3_) δ = 21.4, 38.0, 44.0, 56.1, 113.7, 114.0, 124.9, 125.6, 125.8, 126.8, 127.4, 128.6, 128.8, 128.9, 129.0, 129.1, 129.6, 130.4, 130.7, 130.8, 131.6, 131.8, 133.9, 134.0, 134.3,134.6, 134.7, 135.1, 136.3, 138.5, 139.2, 147.2, 164.4. HRMS-ESI (m/z): calc. for C_37_H_27_N_2_O_2_ [M+H]^+^ 531.2066, found 531.2058.

**(*E*)-(3-Benzyl-2,2-dicyano-6-methyl-2,3-dihydro-1*H*-inden-1-ylidene)(p-tolyl)methyl 4-methylbenzoate (3r):** Yield: 33 mg, 65%; white solid m.p. 251-249 °C. ^1^H NMR (400 MHz, CDCl_3_) δ = 2.17 (s, 3H), 2.42 (s, 3H), 2.50 (s, 3H), 2.26 (dd, *J* = 13.9, 8.3 Hz, 1H), 3.43 (dd, *J* = 13.9, 7.2 Hz, 1H), 4.21 (t, *J* = 7.7 Hz, 1H), 6.78 (d, *J* = 7.8 Hz, 1H), 7.00 (d, *J* = 7.8 Hz, 1H), 7.28 - 7.32 (m, 4H), 7.32 - 7.39 (m, 5H), 7.43 (brs, 1H), 7.64 (d, *J* = 8.3 Hz, 2H), 8.09 (d, *J* = 8.3 Hz, 2H) . ^13^C NMR (100 MHz, CDCl_3_) δ = 21.4, 21.7, 21.9, 37.9, 43.9, 56.1, 113.7, 114.0, 123.7, 124.4, 125.8, 126.0, 127.3, 128.6, 128.8, 129.3, 129.6, 129.7, 130.4, 131.0, 134.2, 134.8, 136.3, 138.3, 139.0, 141.0, 145.2, 147.5, 164.2. HRMS-ESI (m/z): calc. for C_35_H_29_N_2_O_2_ [M+H]^+^ 509.2223, found 509.2227.

**(*E*)-(3-Benzyl-2,2-dicyano-6-methyl-2,3-dihydro-1*H*-inden-1-ylidene)(p-tolyl)methyl 4-isopropylbenzoate (3s):** Yield: 39 mg, 73%; white solid m.p. 171-173 °C. ^1^H NMR (400 MHz, CDCl_3_) δ = 1.34 (d, *J* = 6.8 Hz, 6H), 2.18 (s, 3H), 2.42 (s, 3H), 3.00-3.11 (m, 1H), 3.27 (dd, *J* = 13.9, 8.3 Hz, 1H), 3.43 (dd, *J* = 13.9, 7.1 Hz, 1H), 4.21 (t, 7.5 Hz, 1H), 6.79 (d, *J* = 7.8 Hz, 1H), 7.03 (d, *J* = 7.8 Hz, 1H), 7.27-7.32 (m, 4H), 7.33-7.39 (m, 3H), 7.39-7.46 (m, 3H), 7.64 (d, *J* = 8.3 Hz, 2H), 8.12 (d, *J* = 8.6 Hz, 2H). ^13^C NMR (100 MHz, CDCl_3_) δ = 21.4, 21.7, 23.7, 34.4, 37.9, 43.9, 56.1, 113.7, 114.0, 123.6, 124.4, 125.8, 126.4, 127.0, 127.3, 128.5, 128.8, 129.6, 129.7, 130.4, 130.6, 131.1, 134.8, 136.3, 138.3, 139.0, 141.0, 147.6, 155.9, 164.2. HRMS-ESI (m/z): calc. for C_37_H_33_N_2_O_2_ [M+H]^+^ 537.2536, found 537.2533.

**(*E*)-(3-Benzyl-2,2-dicyano-6-methyl-2,3-dihydro-1*H*-inden-1-ylidene)(p-tolyl)methyl 2-iodobenzoate (3t):** Yield: 52 mg, 84%; white solid; m.p. 172-174 °C. ^1^H NMR (400 MHz, CDCl_3_) δ = 2.26 (s, 3H), 2.45 (s, 3H), 3.27 (dd, *J* = 13.9, 8.3 Hz, 1H), 3.42 (dd, *J* = 13.9, 7.1 Hz, 1H), 4.22 (t, *J* = 8.1 Hz, 1H), 6.79 (d, *J* = 7.8 Hz, 1H), 7.05 (d, *J* = 7.8 Hz, 1H), 7.27 - 7.33 (m, 4H), 7.33 - 7.44 (m, 5H), 7.53 (td, *J* = 7.6, 1.2 Hz, 1H), 7.68 (d, *J* = 8.2 Hz, 2H), 8.05 (dd, *J* = 7.8, 1.7 Hz, 1H), 8.13 (dd, J = 7.9, 1.2 Hz, 1H). ^13^C NMR (100 MHz, CDCl_3_) δ = 21.5, 21.7, 37.9, 44.0, 56.0, 95.0, 113.6, 113.9, 124.4, 124.5, 125.8, 127.3, 128.2, 128.8, 129.0, 129.6, 129.8, 130.5, 130.7, 131.7, 133.1, 133.8, 134.4, 136.3, 138.5, 139.1, 141.2, 142.1, 146.9, 163.7. HRMS-ESI (m/z): calc. for C_34_H_26_IN_2_O_2_ [M+H]^+^ 621.1033, found 621.1033.

**(*E*)-(3-Benzyl-2,2-dicyano-6-methyl-2,3-dihydro-1*H*-inden-1-ylidene)(p-tolyl)methyl 4-(trifluoromethyl)benzoate (3u):** Yield: 29 mg, 52%; white solid m.p. 212-214 °C. ^1^H NMR (400 MHz, CDCl_3_) δ = 2.18 (s, 3H), 2.44 (s, 3H), 3.28 (dd, *J* = 13.9, 8.3 Hz, 1H), 3.43 (dd, *J* = 13.9, 7.2 Hz, 1H), 4.22 (t, *J* = 6.0 Hz, 1H), 6.82 (d, *J* = 8.0 Hz, 1H), 7.07 (dd, *J* = 7.8, 1.0 Hz, 1H), 7.28-7.29 (m, 1H), 7.31-7.35 ( m, 3H), 7.35-7.42 (m, 4H), 7.64 (d, *J* = 8.3 Hz, 2H), 7.85 (d, *J* = 8.4 Hz, 2H), 8.34 (d, *J* = 8.0 Hz, 2H). ^13^C NMR (125 MHz, CDCl_3_) δ = 21.5, 21.7, 37.9, 43.9, 56.1, 113.5, 113.9, 123.5 (q, *J* = 271 Hz), 124.2, 124.6, 125.6, 125.9, 126.0, 127.4, 128.8, 128.9, 129.6, 129.8, 130.5, 130.7, 130.8, 132.0, 134.5, 136.2, 138.6, 139.1, 141.3, 147.0, 163.0.^19^F NMR (471 MHz, CDCl_3_) δ = -63.20. HRMS-ESI (m/z): calc. for C_35_H_26_F_3_N_2_O_2_ [M+H]^+^ 563.1940, found 563,1935.

**(*E*)-(3-Benzyl-2,2-dicyano-5-methoxy-2,3-dihydro-1*H*-inden-1- ylidene)(phenyl)methyl 3-methylbenzoate (3v):** Yield: 39 mg, 76%; white solid; m.p. 145-147 °C. ^1^H NMR (300 MHz, CDCl_3_): δ = 2.48 (s, 3H), 3.21 (dd, *J* = 13.8, 8.9 Hz, 1H), 3.49 (dd, *J* = 13.8, 6.7 Hz, 1H), 3.65 (s, 3H), 4.19 (t, *J* = 7.9 Hz, 1H), 6.32 (s, 1H), 6.78 (d, *J* = 8.7 Hz, 1H), 7.23-7.33 (m, 3H), 7.33-7.44 (m, 3H), 7.45-7.61 (m, 6H), 7.72-7.81 (m, 2H), 8.02 (s, 1H). ^13^C NMR (75 MHz, CDCl_3_): δ = 21.3, 38.0, 43.8, 55.4, 56.0, 109.7, 113.4, 114.2, 115.9, 123.7, 126.3, 126.9, 127.4, 127.5, 128.6, 128.8, 128.9, 129.0, 129.6, 130.4, 130.6, 130.9, 133.9, 135.0, 136.3, 138.8, 143.2, 145.4, 160.7, 164.2. HRMS-ESI (m/z): calc. for C_34_H_27_N_2_O_3_ [M+H]^+^ 511.2015, found 511.2009.

**(*E*)-(3-Benzyl-2,2-dicyano-5-methoxy-2,3-dihydro-1*H*-inden-1- ylidene)(phenyl)methyl 2-bromobenzoate (3w):** Yield: 39 mg, 68%; white solid; m.p. 161-163 °C. ^1^H NMR (300 MHz, CDCl_3_): δ = 3.22 (dd, *J* = 13.8, 8.8 Hz, 1H), 3.47 (dd, *J* = 13.8, 6.7 Hz, 1H), 3.67 (s, 3H), 4.15 - 4.24 (m, 1H), 6.33 (d, *J* = 2.7 Hz, 1H), 6.83 (dd, *J* = 8.8, 2.5 Hz, 1H), 7.24-7.34 (m, 3H), 7.34-7.41 (m, 2H), 7.43-7.51 (m, 2H), 7.51-7.60 (m, 4H), 7.72-7.86 (m, 3H), 8.11 (dd, *J* = 7.1, 2.7 Hz, 1H). ^13^C NMR (100 MHz, CDCl_3_): δ = 37.9, 43.8, 55.4, 56.0, 109.7, 113.4, 114.0, 115.9, 123.1, 124.2, 126.4, 126.7, 127.4, 127.6, 128.8, 129.1, 129.2, 129.6, 129.8, 130.7, 132.4, 133.5, 133.9, 135.2, 136.2, 143.4, 145.0, 160.8, 163.0. HRMS-ESI (m/z): calc. for C_33_H_24_BrN_2_O_3_ [M+H]^+^ 575.0964, found 575.0972.

**(*E*)-(3-Benzyl-2,2-dicyano-5-methoxy-2,3-dihydro-1*H*-inden-1- ylidene)(phenyl)methyl 2-iodobenzoate (3x):** Yield: 44 mg, 71%; white solid; m.p. 127-129 °C. ^1^H NMR (400 MHz, CDCl_3_): δ = 3.20 (dd, *J* = 13.8, 8.9 Hz, 1H), 3.45 (dd, *J* = 13.7, 6.8 Hz, 1H), 3.65 (s, 3H), 4.17 (t, *J* = 7.8 Hz, 1H), 6.30 (d, *J* = 2.7 Hz, 1H), 6.81 (dd, *J* = 8.7, 2.6 Hz, 1H), 7.25-7.29 (m, 3H), 7.29-7.33 (m, 1H), 7.33-7.39 (m, 2H), 7.44-7.59 (m, 5H), 7.77 (dd, *J* = 7.5, 2.1 Hz, 2H), 8.09 (dd, *J* = 7.9, 1.8 Hz, 1H), 8.12 (d, *J* = 7.9 Hz, 1H). ^13^C NMR (100 MHz, CDCl_3_): δ = 37.9, 43.8, 55.4, 56.0, 95.4, 109.7, 113.4, 114.0, 116.0, 124.2, 126.5, 126.6, 127.4, 128.3, 128.8, 129.1, 129.2, 129.6, 130.7, 132.0, 132.5, 133.5, 134.0, 136.2, 142.3, 143.3, 145.0, 160.8, 163.3. HRMS-ESI (m/z): calc. for C_33_H_24_IN_2_O_2_ [M+H]^+^ 623.0825, found 623.0820.

**(*E*)-(3-Benzyl-2,2-dicyano-5-methoxy-2,3-dihydro-1*H*-inden-1- ylidene)(phenyl)methyl 1*H*-indole-2-carboxylate (3y):** Yield: 35 mg, 65%; yellow solid; m.p. 145-147 °C. ^1^H NMR (500 MHz, CDCl_3_): δ = 3.12-3.28 (m, 1H), 3.38-3.52 (m, 1H), 3.65 (s, 3H), 4.07-4.26 (m, 1H), 6.17-6.39 (m, 1H), 6.70-6.90 (m, 1H), 7.06-7.43 (m, 7H), 7.43-7.69 (m, 6H), 7.71-7.87 (m, 2H), 8.03-8.17 (m, 2H). ^13^C NMR (125 MHz, CDCl_3_): δ = 37.9, 43.9, 55.4, 56.0, 95.2, 109.8, 113.4, 114.0, 116.0, 124.2, 124.3, 126.5, 126.7, 127.4, 128.3, 128.8, 129.0, 129.3, 129.6, 130.7, 132.0, 132.6, 132.7, 133.5, 133.9, 136.2, 142.3, 143.4, 145.0, 160.9, 163.3. HRMS-ESI (m/z): calc. for C_35_H_26_N_3_O_3_ [M+H]^+^ 536.1968, found 536.1970.

**Ethyl (*E*)-1-benzyl-2-cyano-3-(((2-iodobenzoyl)oxy)(m-tolyl)methylene)-2,3-dihydro-1*H*-indene-2-carboxylate (3z):** Yield: 49 mg, 75%; white solid m.p. 181-179 °C. ^1^H NMR (500 MHz, CDCl_3_) δ = 0.79 (t, *J* = 7.2 Hz, 3H), 2.37 (s, 3H), 3.00-3.15 (m, 2H), 3.24 (dd, *J* = 14.3, 10.4 Hz, 1H), 3.60 (dd, *J* = 14.3, 5.4 Hz, 1H), 4.37 (dd, *J* = 10.3, 5.4 Hz, 1H), 7.15-7.24 (m, 3H), 7.24-7.28 (m, 3H), 7.29-7.37 (m, 5H), 7.44-7.51 (m, 2H), 7.53 (s, 1H), 7.60 (d, *J* = 7.9 Hz, 1H), 8.05 (dd, *J* = 7.9, 1.7 Hz, 1H), 8.09 (d, *J* = 8.0 Hz, 1H). ^13^C NMR (125 MHz, CDCl_3_) δ = 13.1, 21.3, 36.6, 55.3, 58.4, 62.7, 95.1, 117.4, 123.5, 125.4, 126.7, 126.8, 128.1, 128.2, 128.4, 128.6, 128.7, 129.5, 129.7, 130.4, 130.5, 131.9, 133.0, 133.6, 133.7, 135.9, 137.1, 137.9, 142.2, 143.3, 145.8, 163.4, 167.0. HRMS-ESI (m/z): calc. for C_35_H_29_INO_4_ [M+H]^+^ 654.1135, found 654.1141.

**(*E*)-(3-Benzyl-2-cyano-2-(ethoxycarbonyl)-2,3-dihydro-1*H*-inden-1-ylidene)(m-tolyl)methyl 1-naphthoate (3a'):** Yield: 51 mg, 88%; white solid m.p. 155-153 °C. ^1^H NMR (500 MHz, CDCl_3_) δ **=** 0.84 (t, *J* = 7.2 Hz, 3H), 2.39 (s, 3H), 3.05-3.20 (m, 2H), 3.29 (dd, *J =* 14.4*,* 10.4 Hz, 1H), 3.64 (dd, *J* = 14.4, 5.4 Hz, 1H), 4.44 (dd, *J* = 10.3, 5.4 Hz, 1H), 7.13-7.27 (m, 4H), 7.28-7.33 (m, 3H), 7.34-7.40 (m, 3H), 7.52-7.61 (m, 4H), 7.62-7.72 (m, 2H), 7.94 (dd, *J* = 8.2, 1.8 Hz, 1H), 8.14 (d, *J* = 8.2 Hz, 1H), 8.53 (dd, *J* = 7.2, 1.5 Hz, 1H), 9.07 (d, *J* = 8.6 Hz, 1H). ^13^C NMR (125 MHz, CDCl_3_) δ = 13.1, 21.4, 36.7, 55.4, 58.4, 62.7, 117.6, 123.5, 124.6, 125.0 125.2, 125.9, 126.5, 126.6, 126.8, 128.3, 128.4, 128.5, 128.6, 128.7, 128.8, 129.4, 129.7, 130.2, 130.4, 131.8, 131.9, 134.0, 134.3, 134.9, 136.2, 137.2, 138.0, 143.2, 146.2, 164.5, 167.1. HRMS-ESI (m/z): calc. for C_39_H_32_NO_4_ [M+H]^+^ 578.2326, found 578.2329.

**Methyl (*E*)-1-benzyl-2-cyano-3-(((2-iodobenzoyl)oxy)(p-tolyl)methylene)-5-methyl-2,3-dihydro-1*H*-indene-2-carboxylate (3b'):** Yield: 44 mg, 67%; white solid m.p. 172-174 °C. ^1^H NMR (500 MHz, CDCl_3_) δ **=** 2.26 (s, 3H), 2.34 (s, 3H), 2.77 (s, 3H), 3.19 (dd, *J* = 14.5, 10.7 Hz, 1H), 3.58 (dd, *J* = 14.5, 5.3 Hz, 1H), 4.32 (dd, *J* = 10.5, 5.3 Hz, 1H), 7.13 (s, 2H), 7.17-7.25 (m, 4H), 7.27-7.35 (m, 4H), 7.42 (s, 1H), 7.47 (t, *J* =7.6 Hz, 1H), 7.56 (d, *J* = 8.0 Hz, 2H), 7.99 (dd, *J* = 7.9, 1.7 Hz, 1H), 8.07 (d, *J* = 7.8 Hz, 1H). ^13^C NMR (125 MHz, CDCl_3_) δ = 21.4, 21.5, 36.6, 52.8, 55.1, 58.5, 94.7, 117.5, 123.2, 125.9, 126.8, 128.1, 128.4, 128.7, 128.9, 129.6, 129.7, 130.4, 130.9, 131.6, 133.5, 135.9, 136.8, 137.0, 138.3, 139.8, 140.4, 142.0, 145.4, 163.8, 167.4. HRMS-ESI (m/z): calc. for C_35_H_29_INO_4_ [M+H]^+^ 654.1135, found 654.1132.

**(*E*)-(3-Benzyl-2,2-dicyano-2,3-dihydro-1*H*-inden-1-ylidene)(phenyl)methyl 2-phenylacetate (3c'):** Yield: 32 mg, 67%; white solid m.p. 134-131 °C. ^1^H NMR (400 MHz, CDCl_3_) δ = 3.23 (dd, *J* = 14.0, 8.4 Hz, 1H), 3.39 (dd, *J* = 14.0, 7.0 Hz, 1H), 3.87 (s, 2H), 4.17 (t, *J* = 7.8 Hz, 1H), 6.87 (dd, *J* = 8.0, 1.0 Hz, 1H), 7.12 (tt, *J* = 7.6, 1.2 Hz, 1H), 7.18-7.28 (m, 4H), 7.29-7.35 (m, 4H), 7.36-7.42 (m, 4H), 7.43-7.53 (m, 3H), 7.55-7.61 (m, 2H). ^13^C NMR (100 MHz, CDCl_3_) δ = 37.7, 41.8, 43.6, 56.2, 113.4, 113.7, 124.1, 124.7, 125.3, 127.4, 127.8, 128.4, 128.8, 129.0, 129.2, 129.5, 129.6, 129.7, 130.8, 132.2, 132.5, 133.5, 134.3, 136.1, 141.1, 147.3, 168.5. HRMS-ESI (m/z): calc. for C_33_H_25_N_2_O_2_ [M+H]^+^ 481.1910, found 481.1914.

**(*E*)-(2,2-Dicyano-3-(3-methylbenzyl)-2,3-dihydro-1*H*-inden-1-ylidene)(phenyl)methyl 2-iodobenzoate (3d'):** Yield: 55 mg, 90%; white solid; m.p. 174-176 °C. ^1^H NMR (500 MHz, CDCl_3_) δ = 2.37 (s, 3H), 3.28 (dd, *J* = 13.9, 8.4 Hz, 1H), 3.41 (dd, *J* = 13.9, 7.0 Hz, 1H), 4.27 (t, *J* = 7.7 Hz, 1H), 6.95 (d, *J* = 7.7 Hz, 1H), 7.17 – 7.07 (m, 3H), 7.33 – 7.24 (m, 4H), 7.60 – 7.50 (m, 4H), 7.64 (d, *J* = 7.5 Hz, 1H), 7.82 (d, *J* = 8.0 Hz, 2H), 8.18 – 8.09 (m, 2H). ^13^C NMR (125 MHz, CDCl_3_) δ = 21.5, 37.7, 43.8, 56.1, 95.4, 113.5, 113.8, 124.8, 124.9, 125.4, 126.7, 128.2, 128.4, 128.7, 129.1, 129.2, 129.4, 129.9, 130.3, 131.0, 132.1, 132.4, 133.4, 134.1, 134.3, 136.0, 138.5, 141.5, 142.4, 147.1, 163.3. HRMS-ESI (m/z): calc. for C_33_H_24_IN_2_O_2_ [M+H]^+^ 607.0876, found 607.0873.

**(*E*)-(2,2-Dicyano-3-(4-methylbenzyl)-2,3-dihydro-1*H*-inden-1-ylidene)(phenyl)methyl 2-iodobenzoate (3e'):** Yield: 53 mg, 88%; white solid; m.p. 175-177 °C. ^1^H NMR (500 MHz, CDCl_3_) δ = 2.37 (s, 3H), 3.28 (dd, *J* = 14.0, 8.2 Hz, 1H), 3.40 (dd, *J* = 14.0, 7.2 Hz, 1H), 4.24 (t, *J* = 7.7 Hz, 1H), 6.97 (d, *J* = 7.4 Hz, 1H), 7.18 (s, 3H), 7.33 – 7.24 (m, 4H), 7.58 – 7.50 (m, 4H), 7.63 (d, *J* = 8.7 Hz, 1H), 7.82 (d, *J* = 8.0 Hz, 2H), 8.12 (t, *J* = 8.5 Hz, 2H). ^13^C NMR (125 MHz, CDCl_3_) δ = 21.2, 37.4, 43.7, 56.3, 95.4, 113.5, 113.8, 124.8, 124.9, 125.4, 128.4, 129.1, 129.2, 129.3, 129.4, 129.5, 129.9, 130.9, 132.1, 132.4, 133.0, 133.4, 134.1, 134.3, 137.0, 141.5, 142.4, 147.1, 163.3. HRMS-ESI (m/z): calc. for C_33_H_24_IN_2_O_2_ [M+H]^+^ 607.0876, found 607.0881.

**5. ^1^H and ^13^C NMR spectra of products**

^1^H NMR (400 MHz, CDCl_3_) spectrum of **3a**


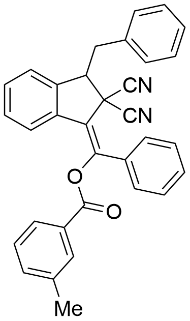
**
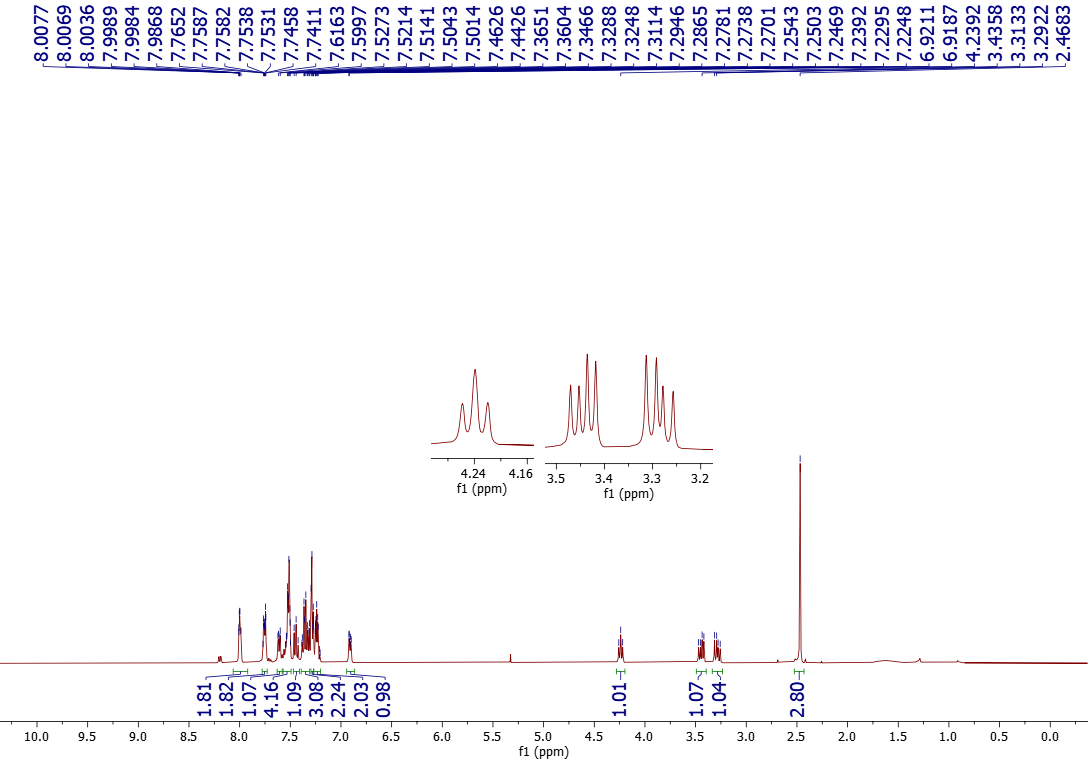
**

^13^C NMR (100 MHz, CDCl_3_) spectrum of **3a**


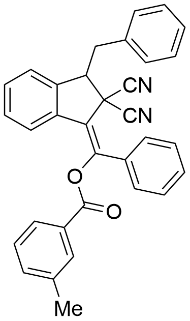

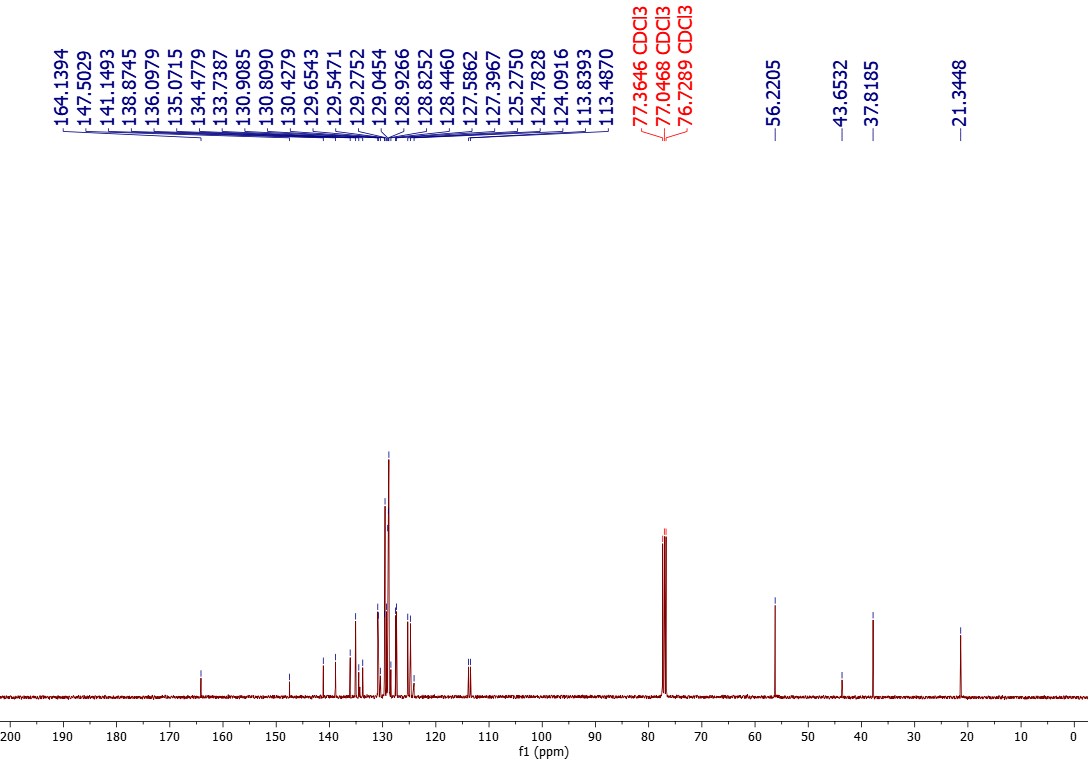


HRMS-ESI Compound **3a**


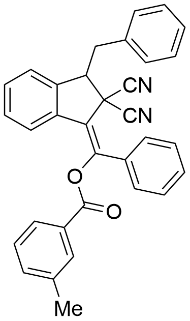

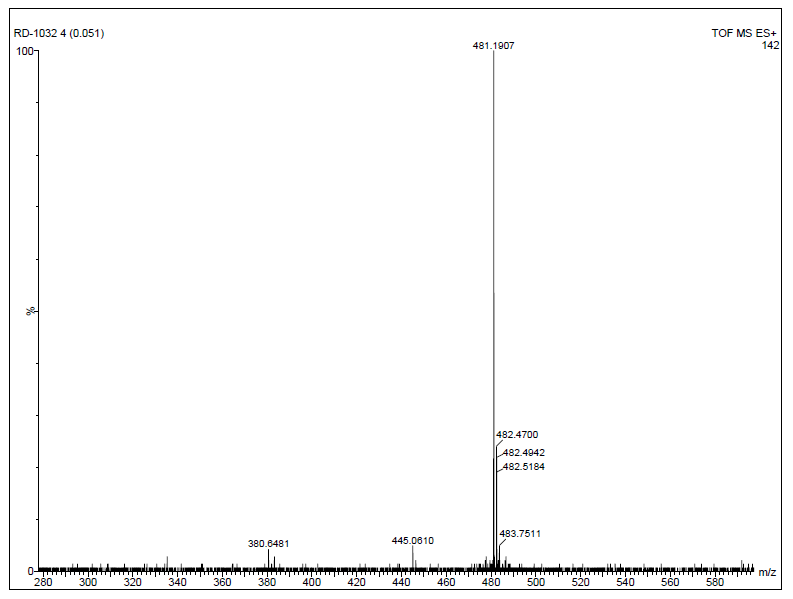


[M+H]^+^

^1^H NMR (400 MHz, CDCl_3_) spectrum of **3b**


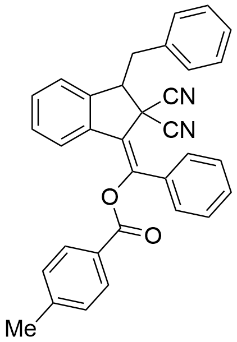

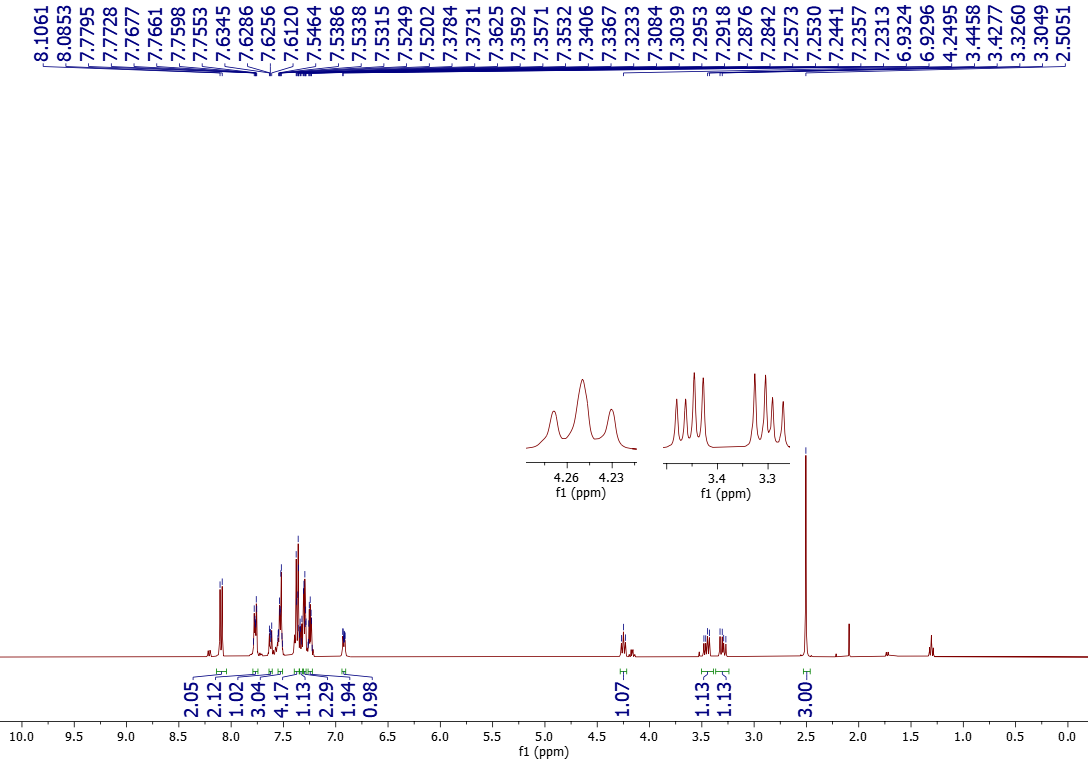


^13^C NMR (100 MHz, CDCl_3_) spectrum of **­3b**


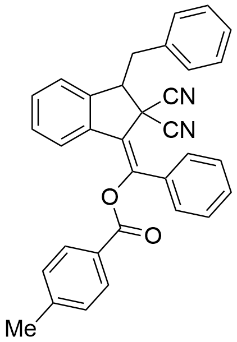

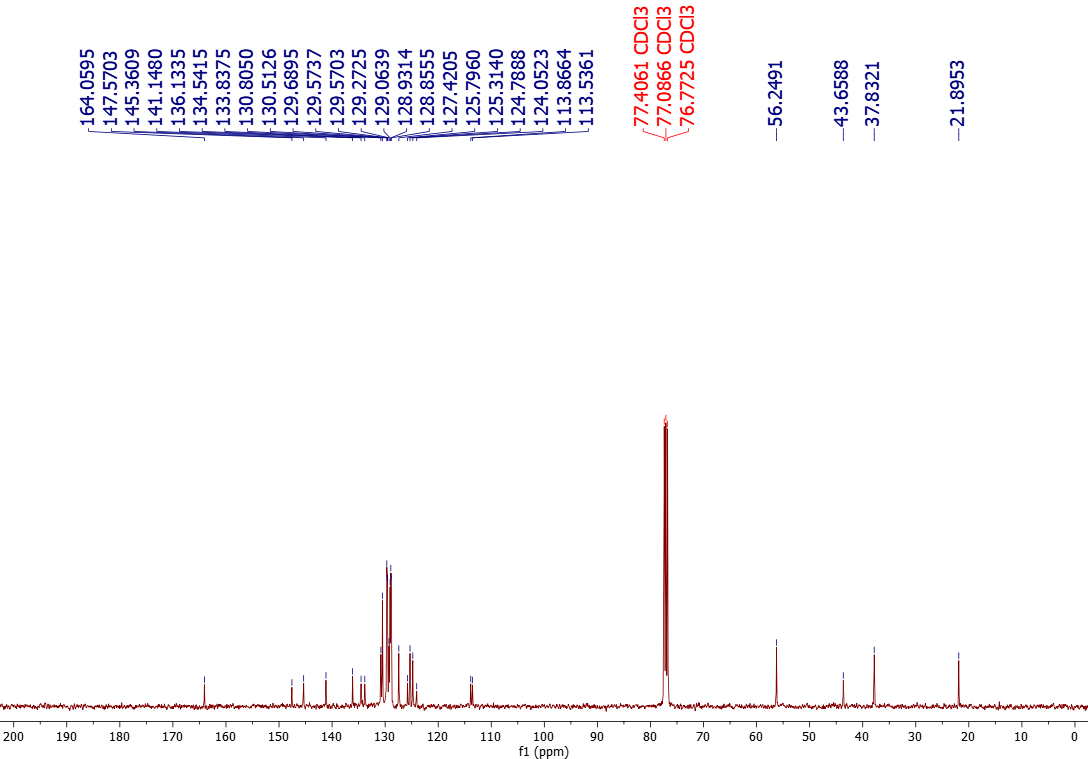


HRMS-ESI Compound **3b**


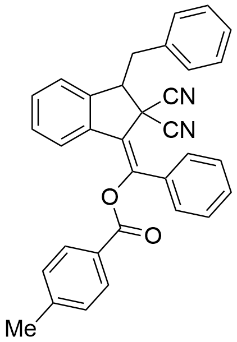

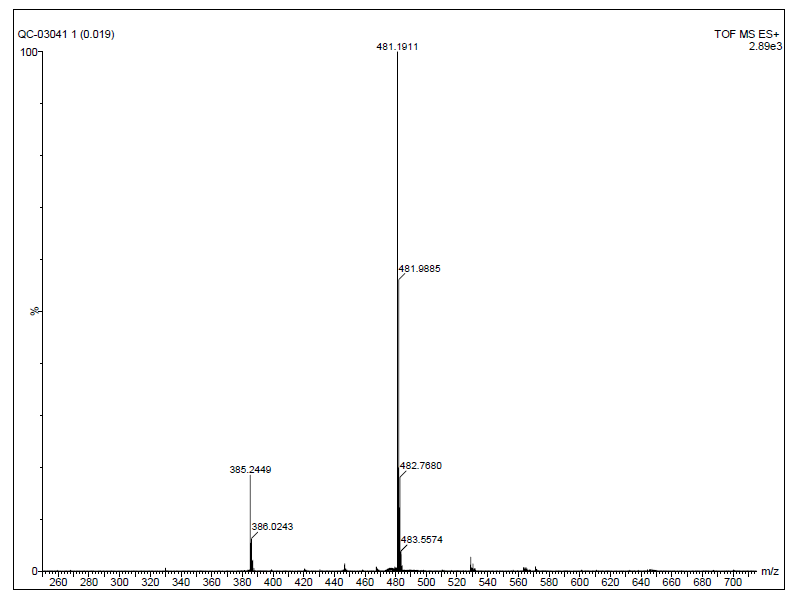


[M+H]^+^

^1^H NMR (300 MHz, CDCl_3_) spectrum of **3c**


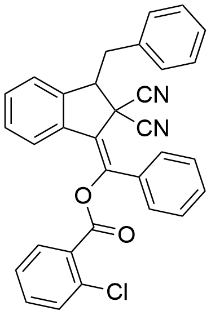

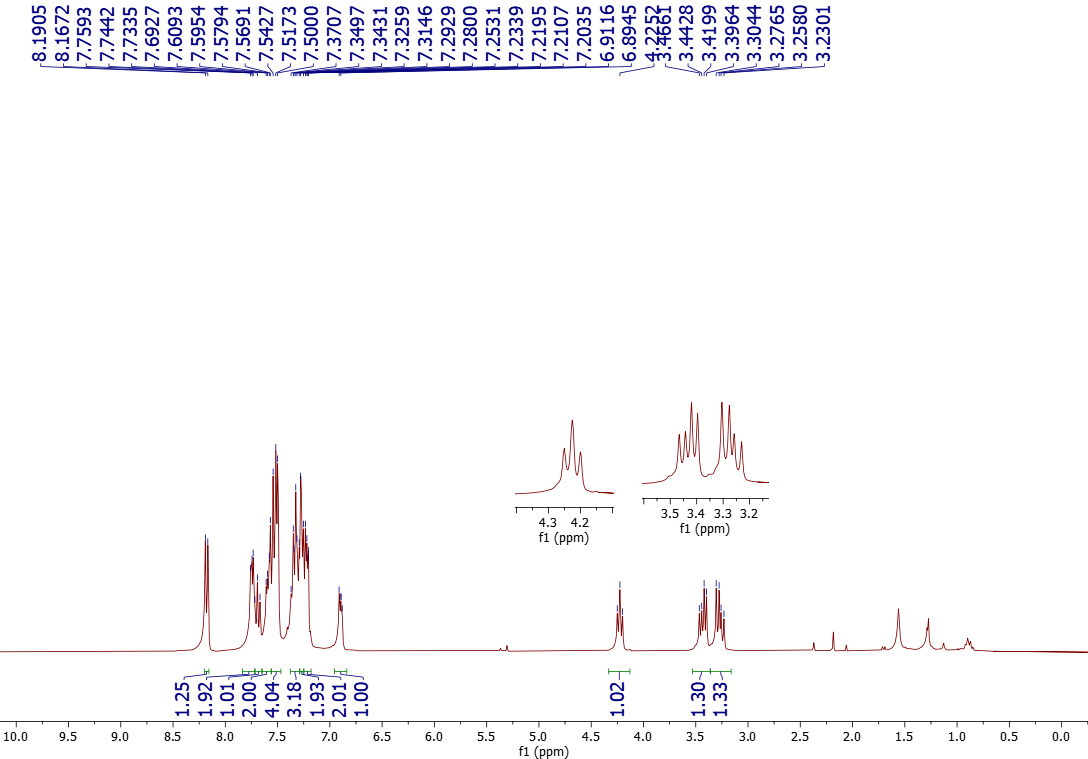


^13^C NMR (75 MHz, CDCl_3_) spectrum of **3c**


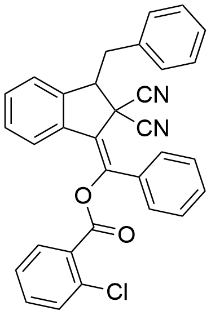
**
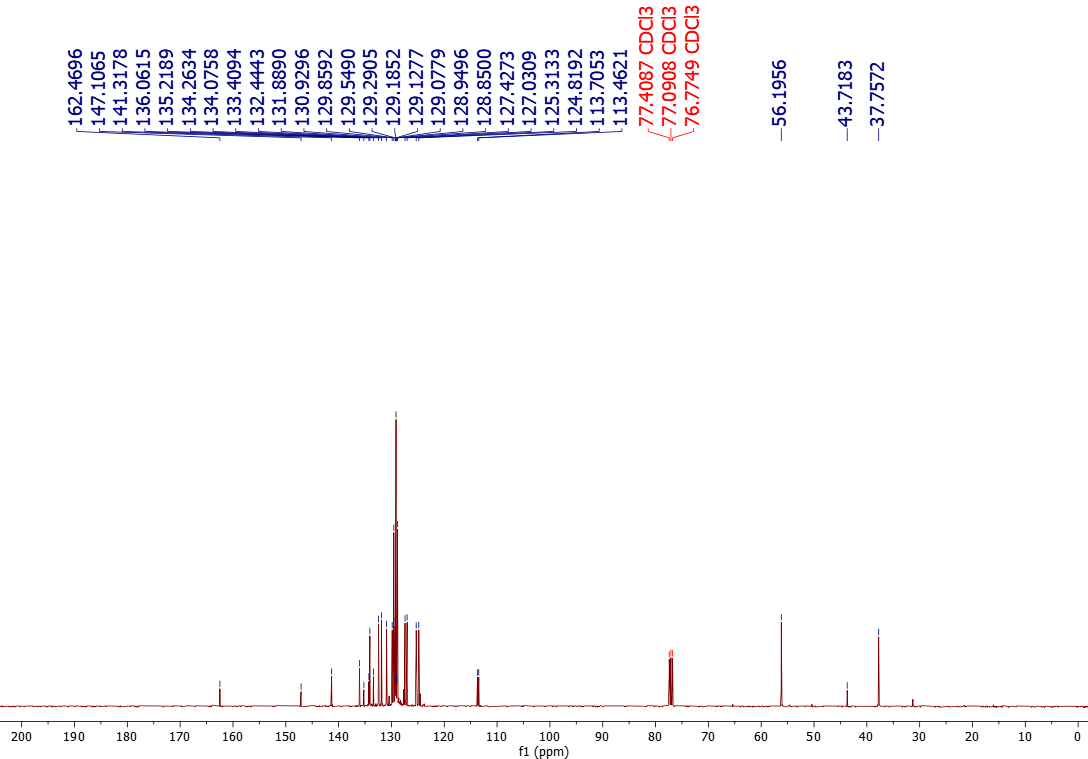
**

HRMS-ESI Compound **3c**


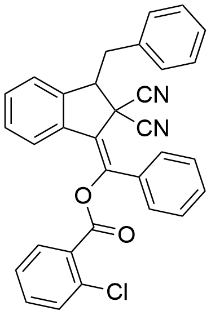

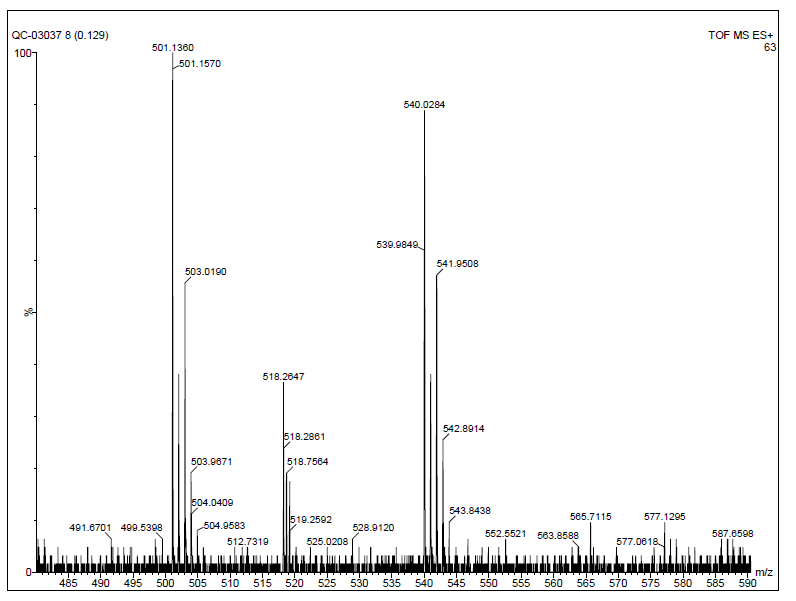


[M+H]^+^

^1^H NMR (400 MHz, CDCl_3_) spectrum of **3d**


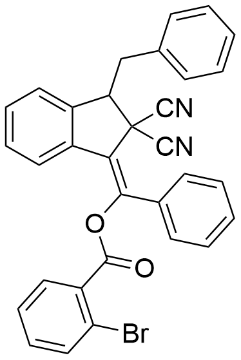

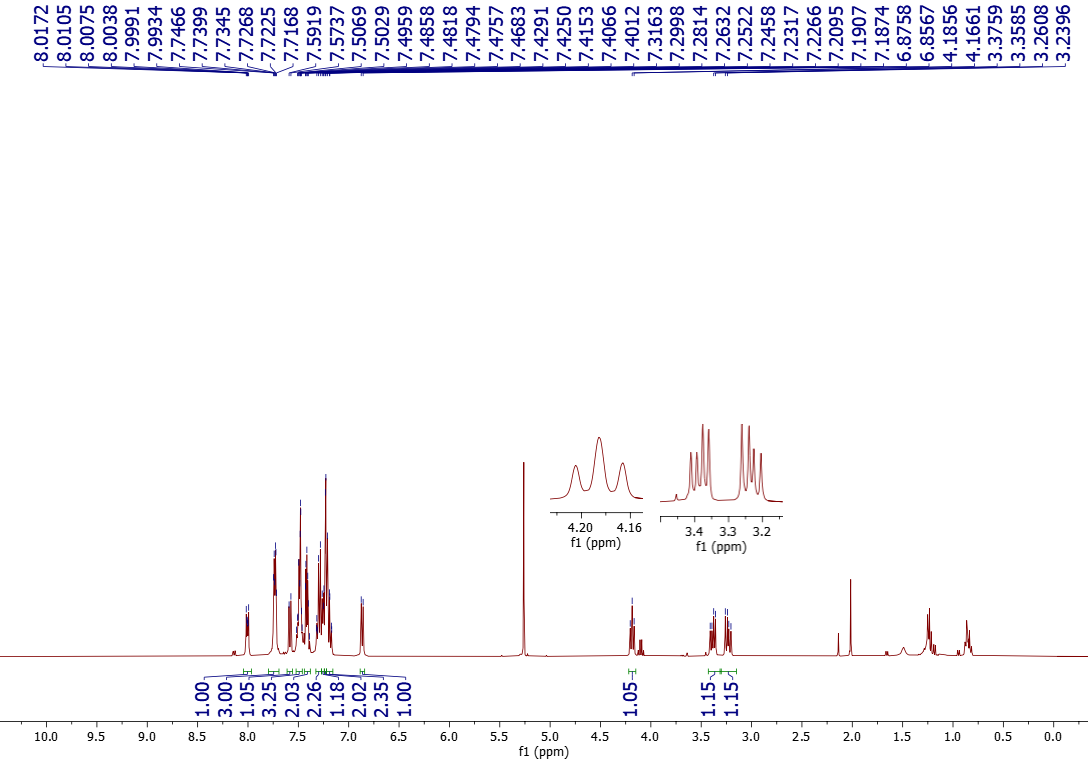


^13^C NMR (100 MHz, CDCl_3_) spectrum of **3d**


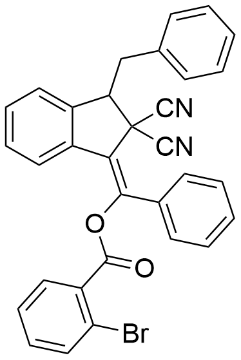

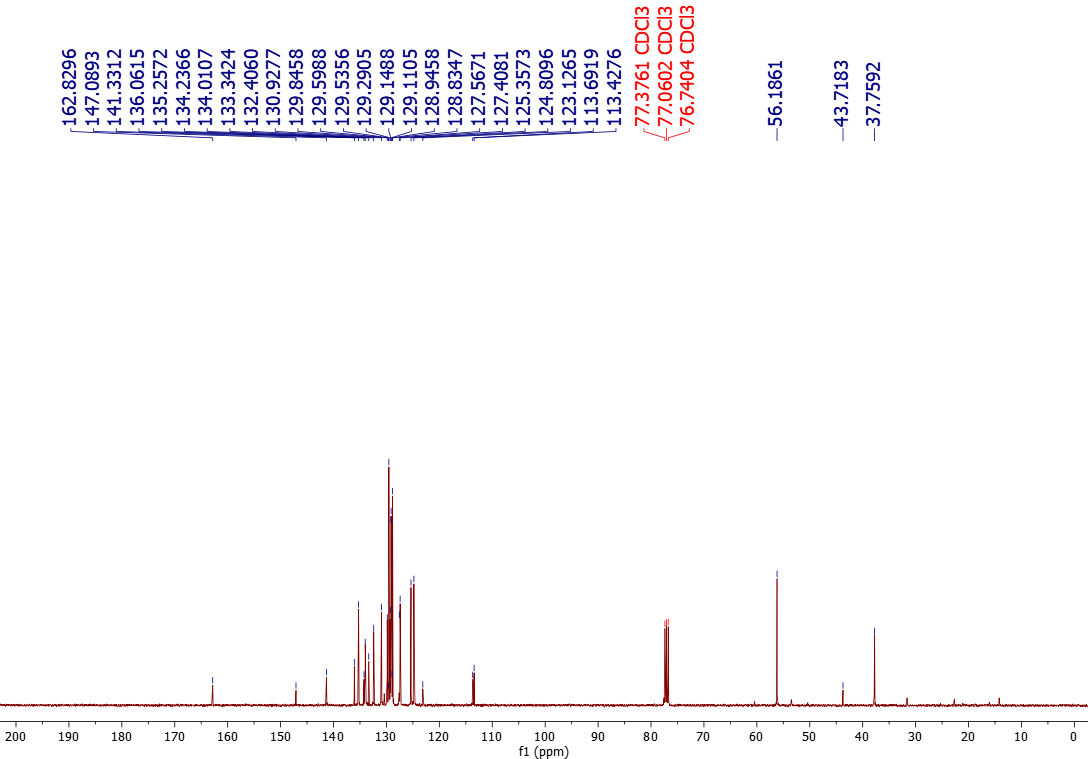


HRMS-ESI Compound **3d**


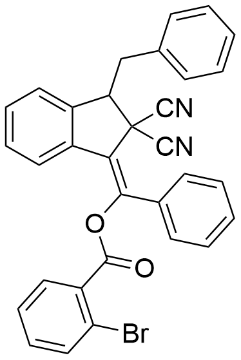

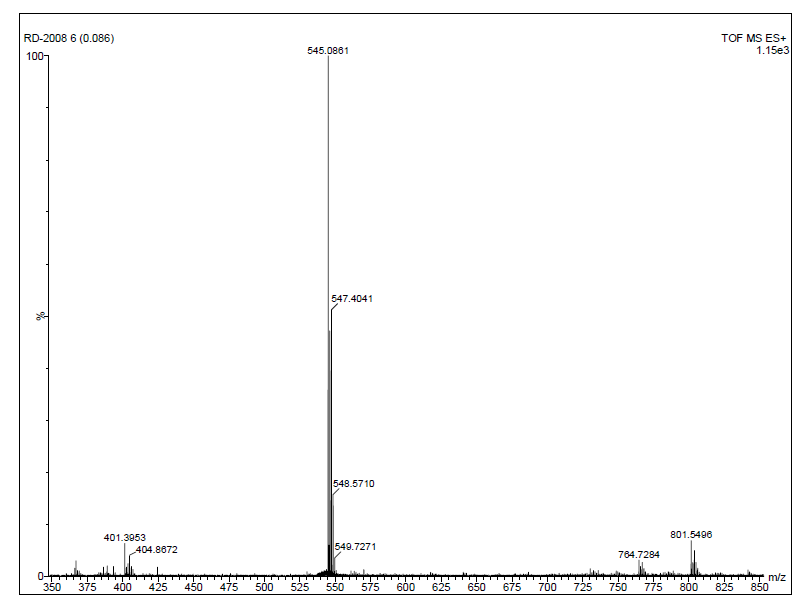


[M+H]^+^

^1^H NMR (400 MHz, CDCl_3_) spectrum of **3e**


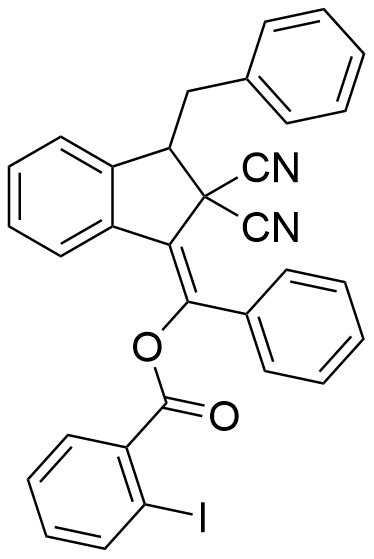

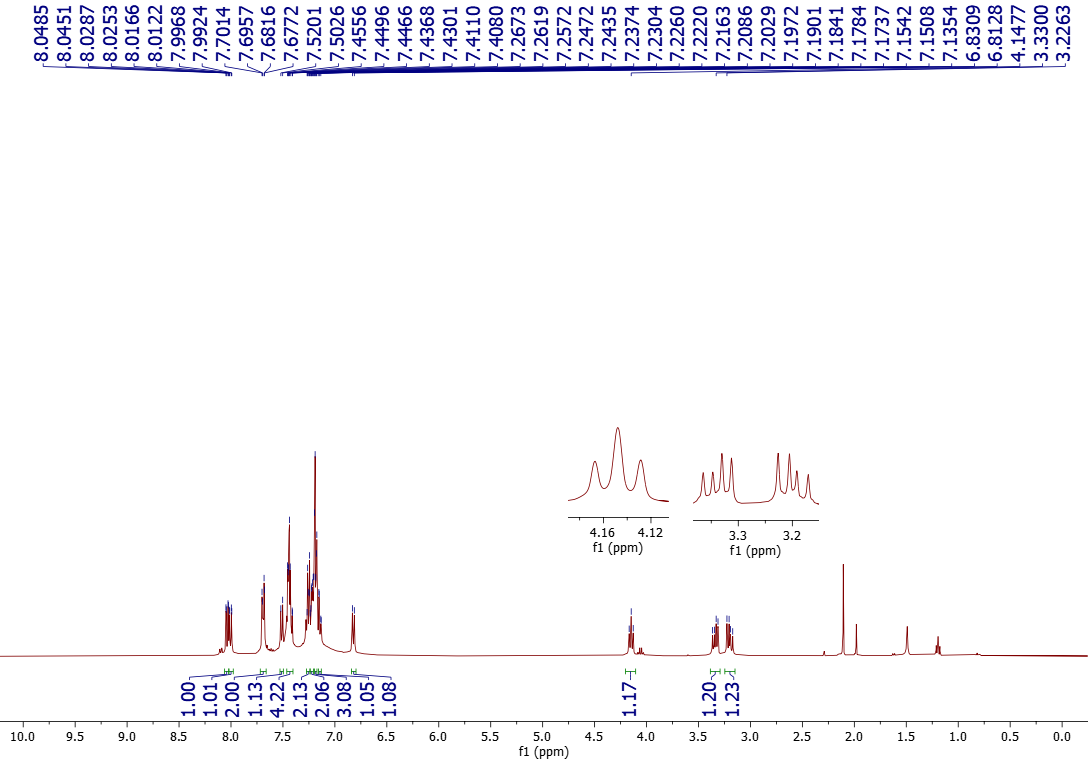


^13^C NMR (100 MHz, CDCl_3_) spectrum of **3e**


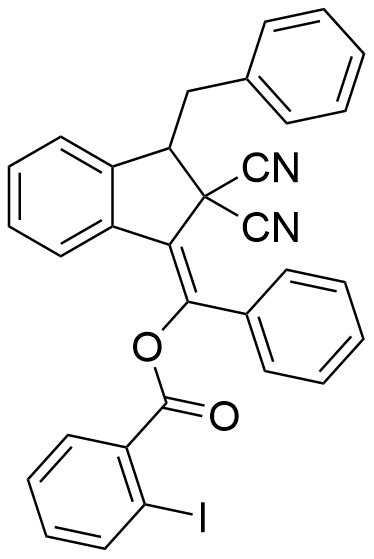

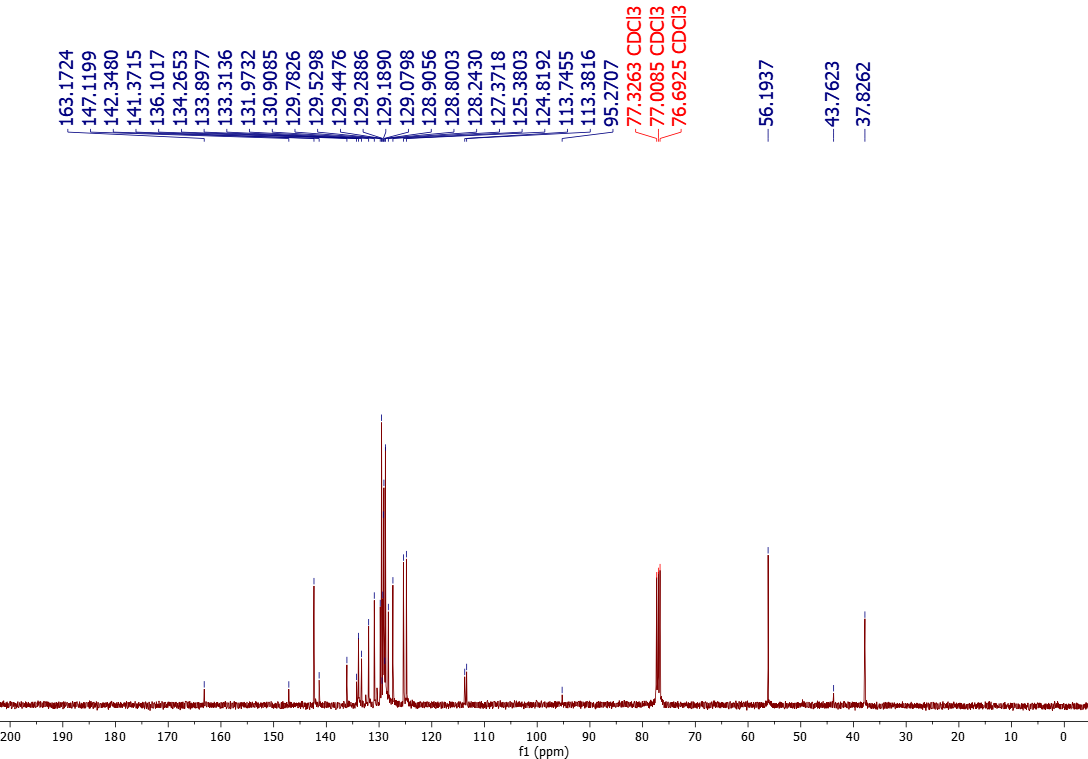


HRMS-ESI Compound **3e**


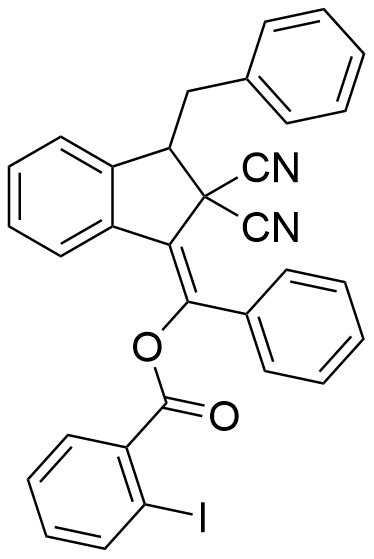

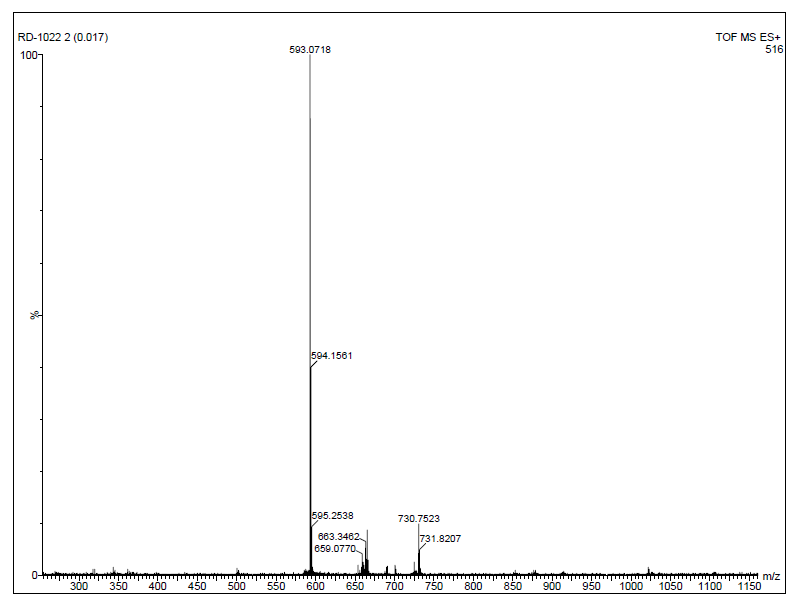


[M+H]^+^

^1^H NMR (300 MHz, CDCl_3_) spectrum of **3f**


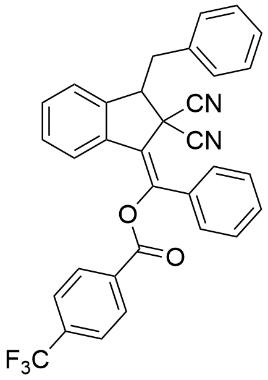

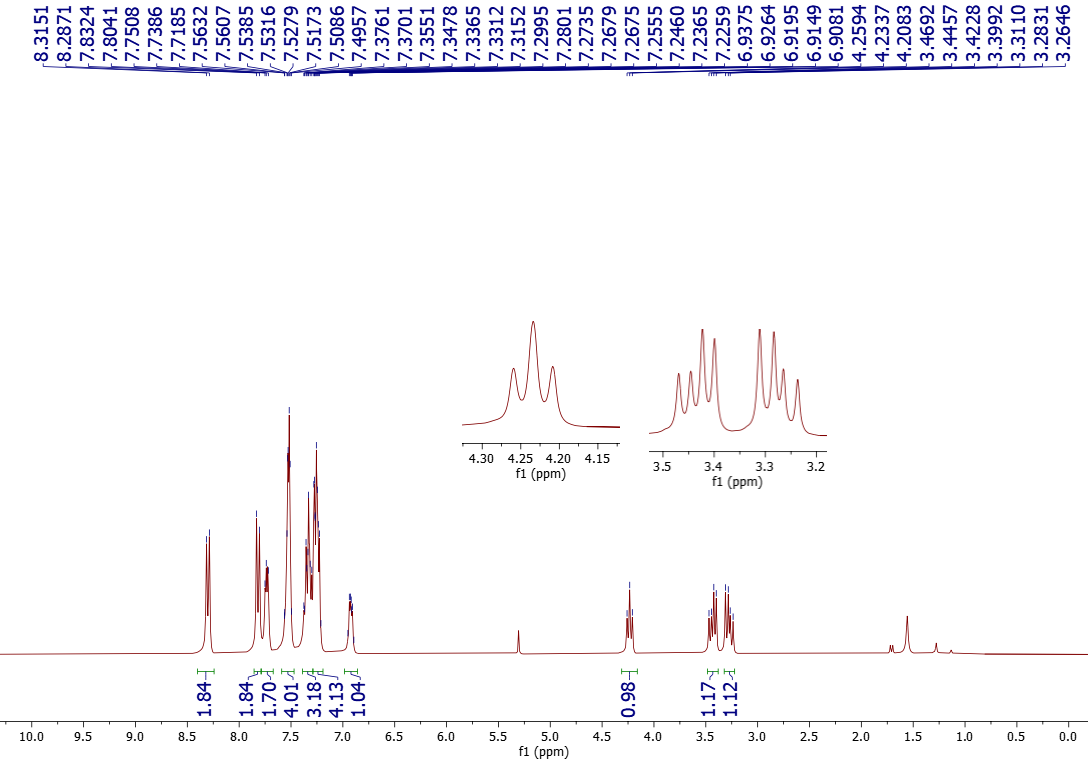


^13^C NMR (125 MHz, CDCl_3_) spectrum of **3f**


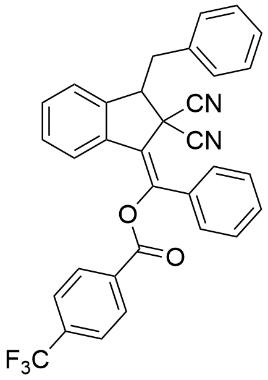

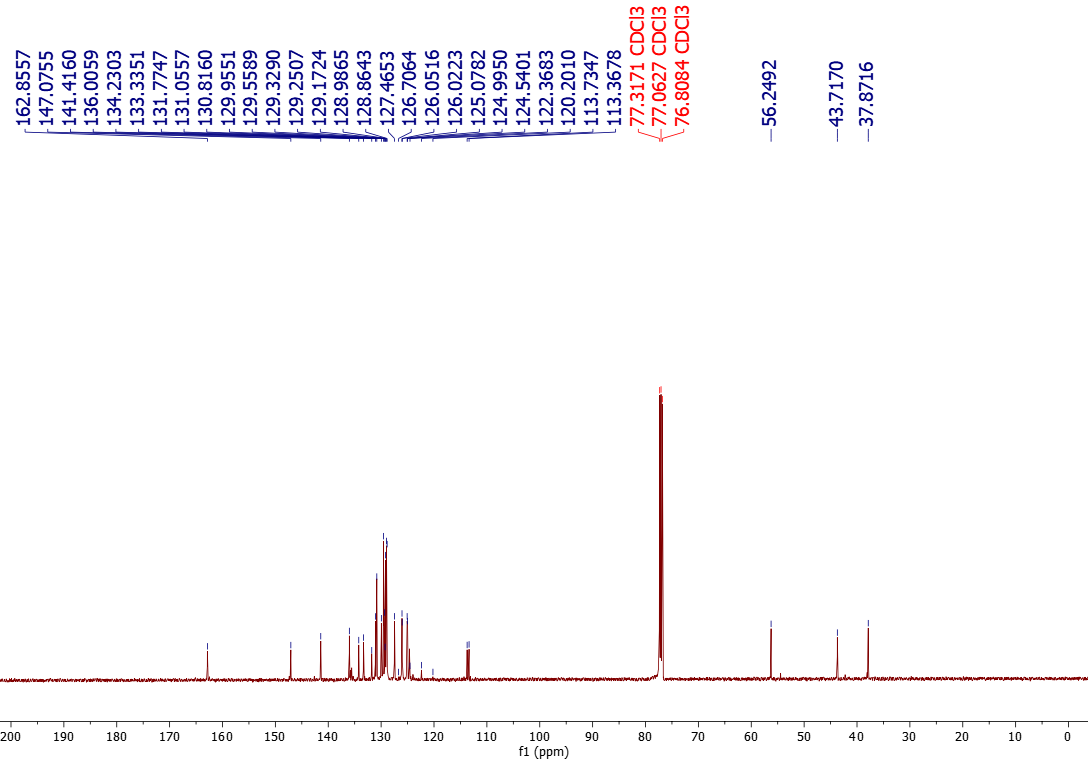


HRMS-ESI Compound **3f**


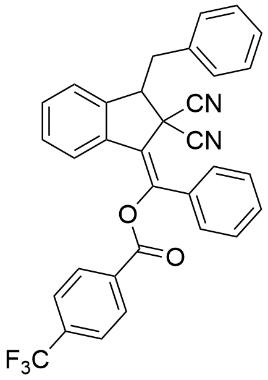

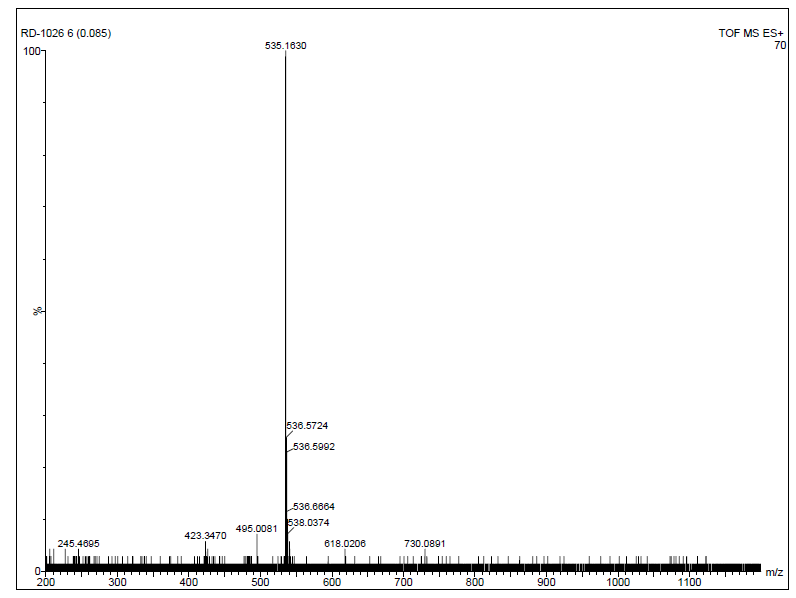


[M+H]^+^

^1^H NMR (400 MHz, CDCl_3_) spectrum of **3g**


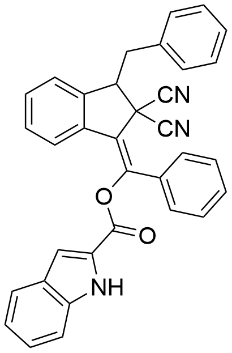

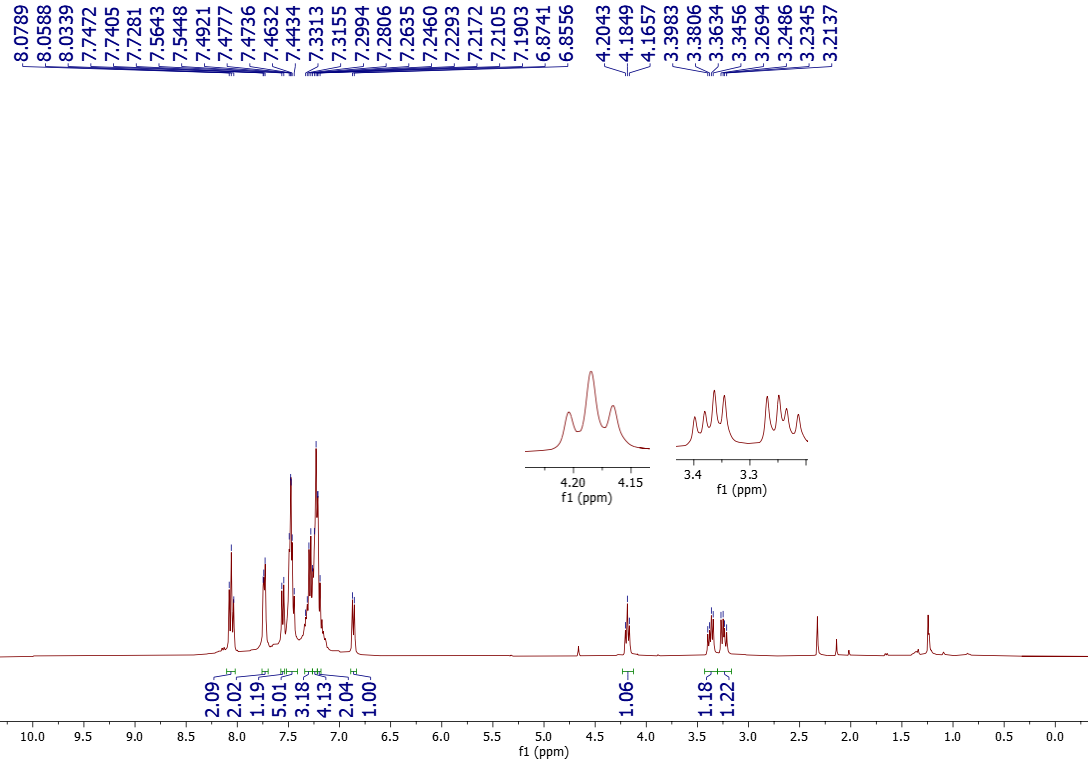


^13^C NMR (100 MHz, CDCl_3_) spectrum of **3g**


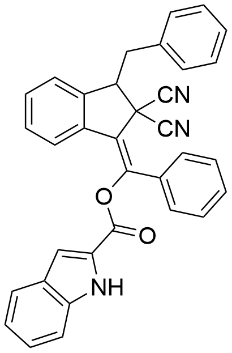

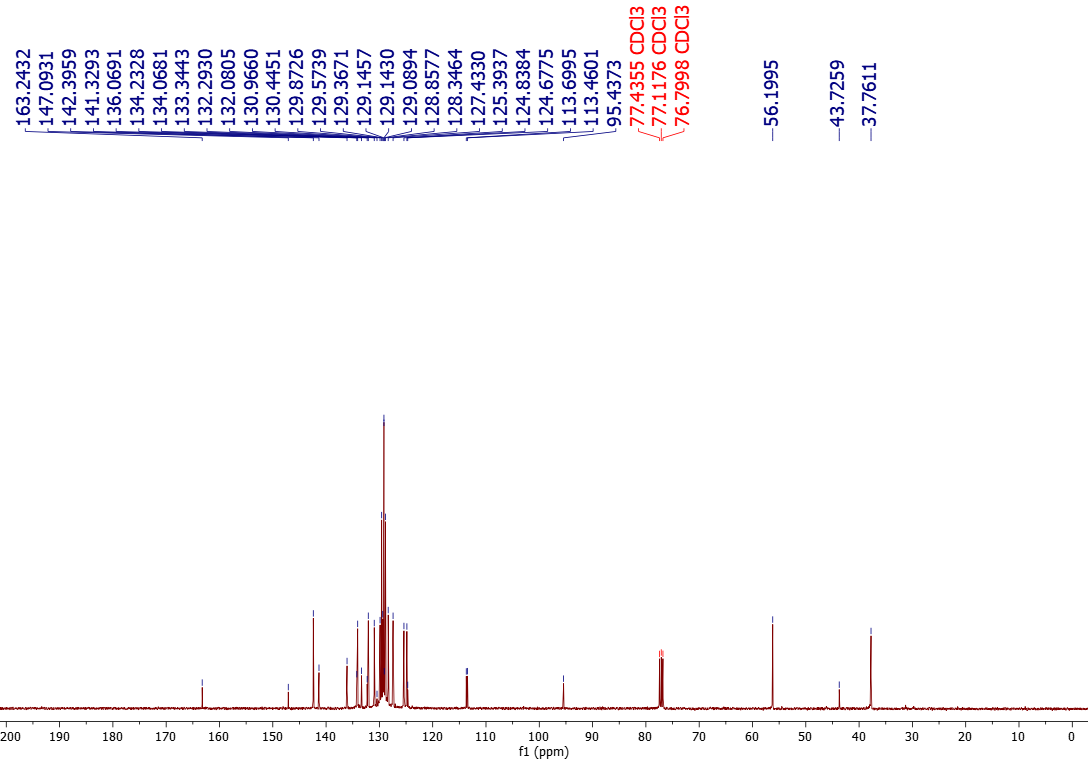


HRMS-ESI Compound **3g**


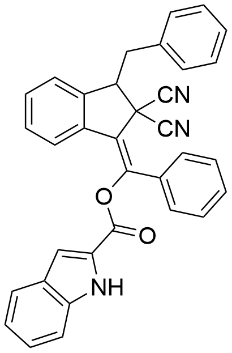

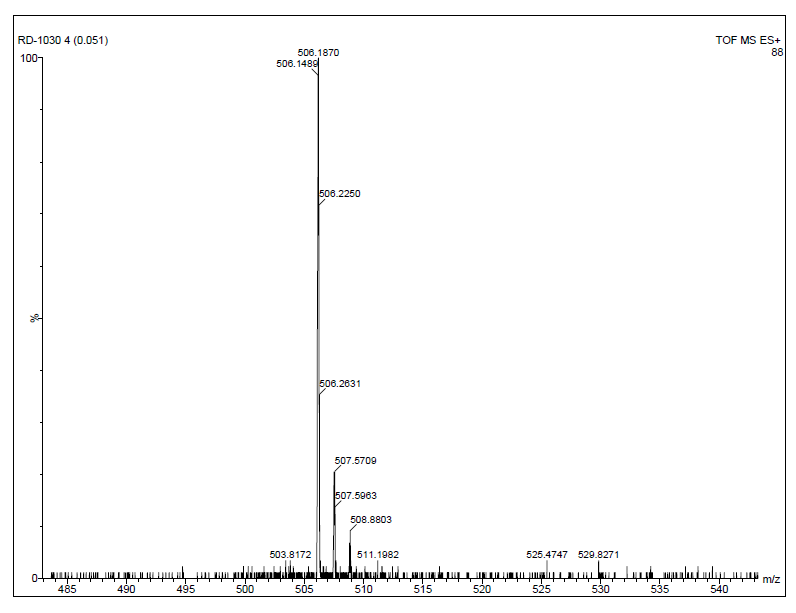


[M+H]^+^

^1^H NMR (400 MHz, CDCl_3_) spectrum of **3h**


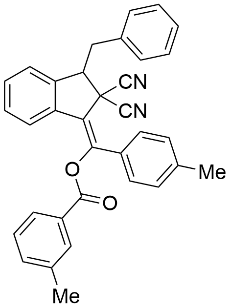

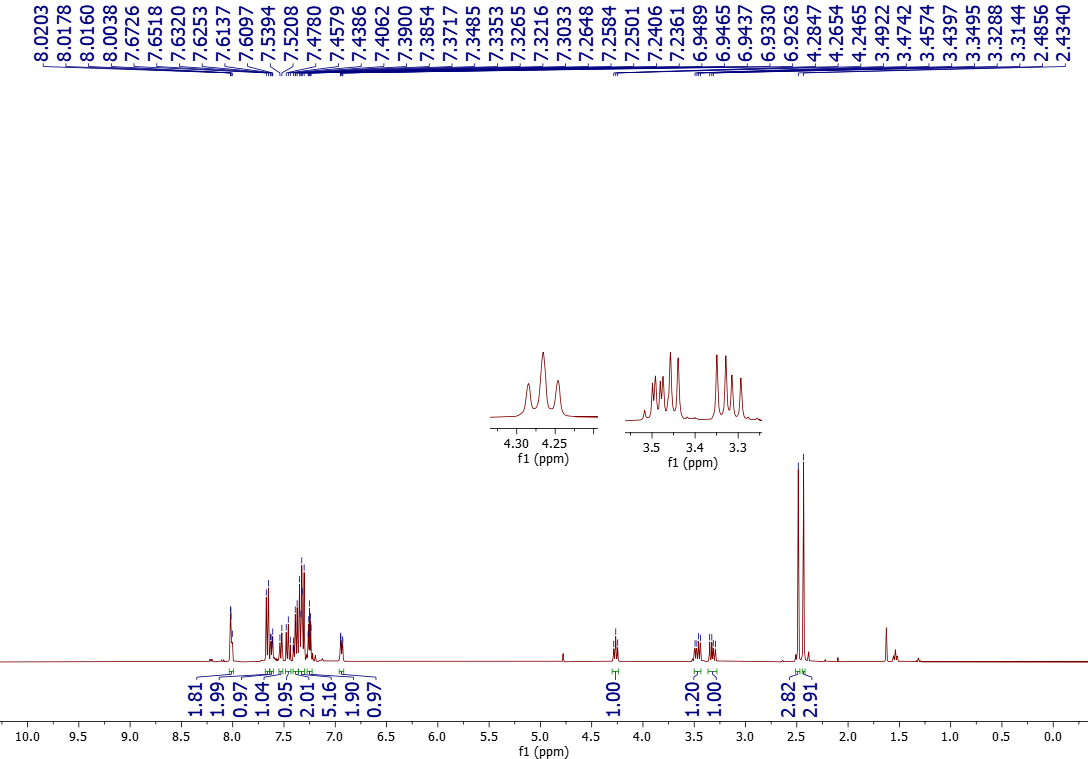


^13^C NMR (100 MHz, CDCl_3_) spectrum of **3h**


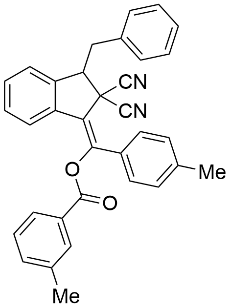
**
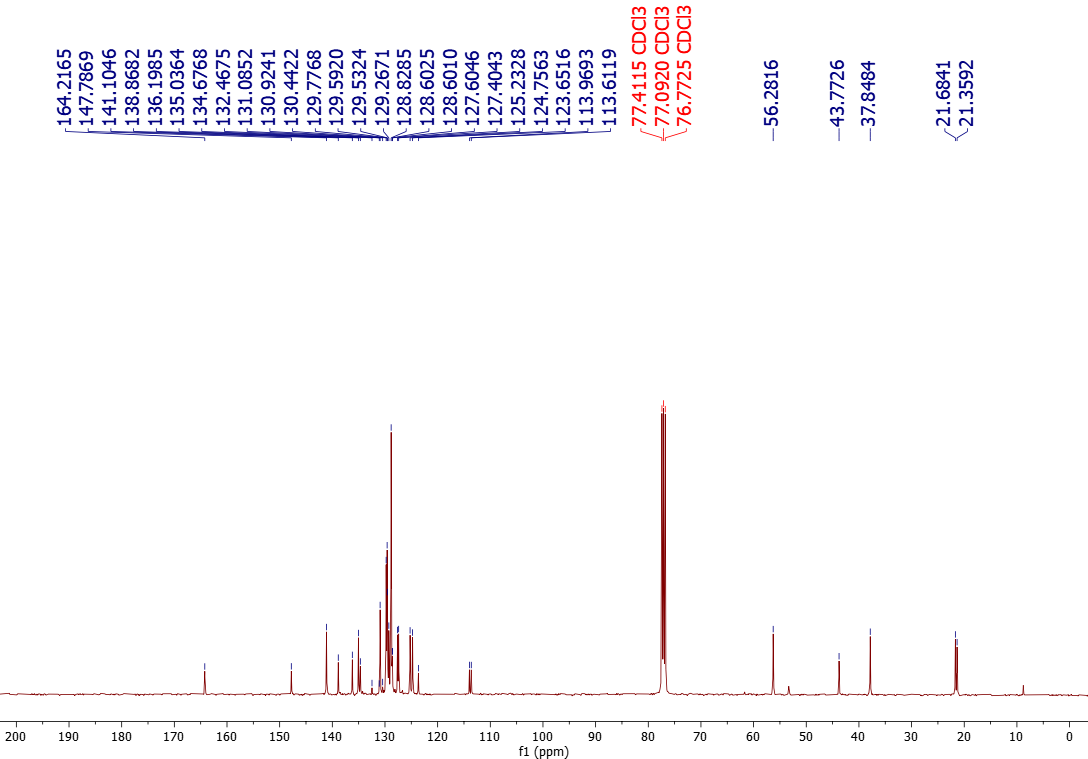
**

HRMS-ESI Compound **3h**


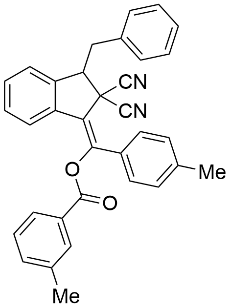

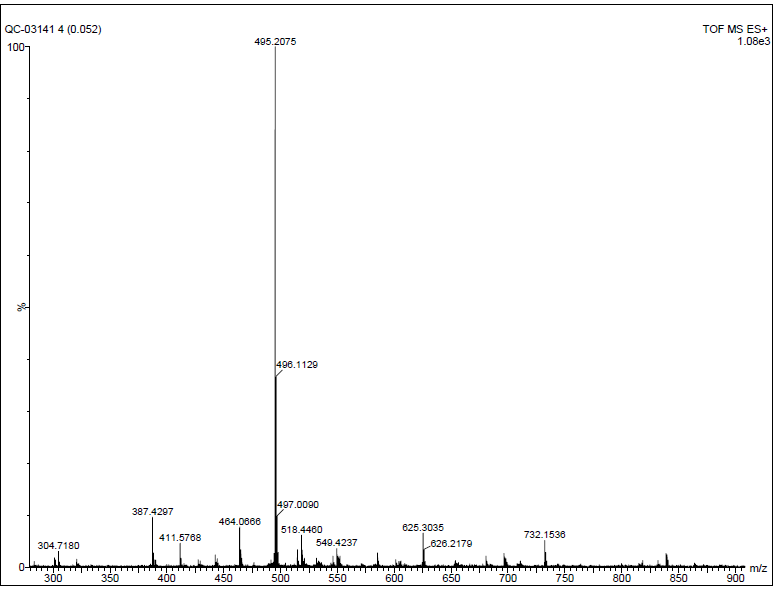


[M+H]^+^

^1^H NMR (500 MHz, CDCl_3_) spectrum of **3i**


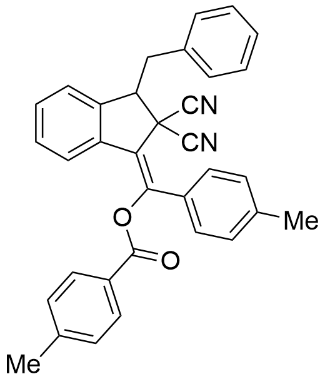

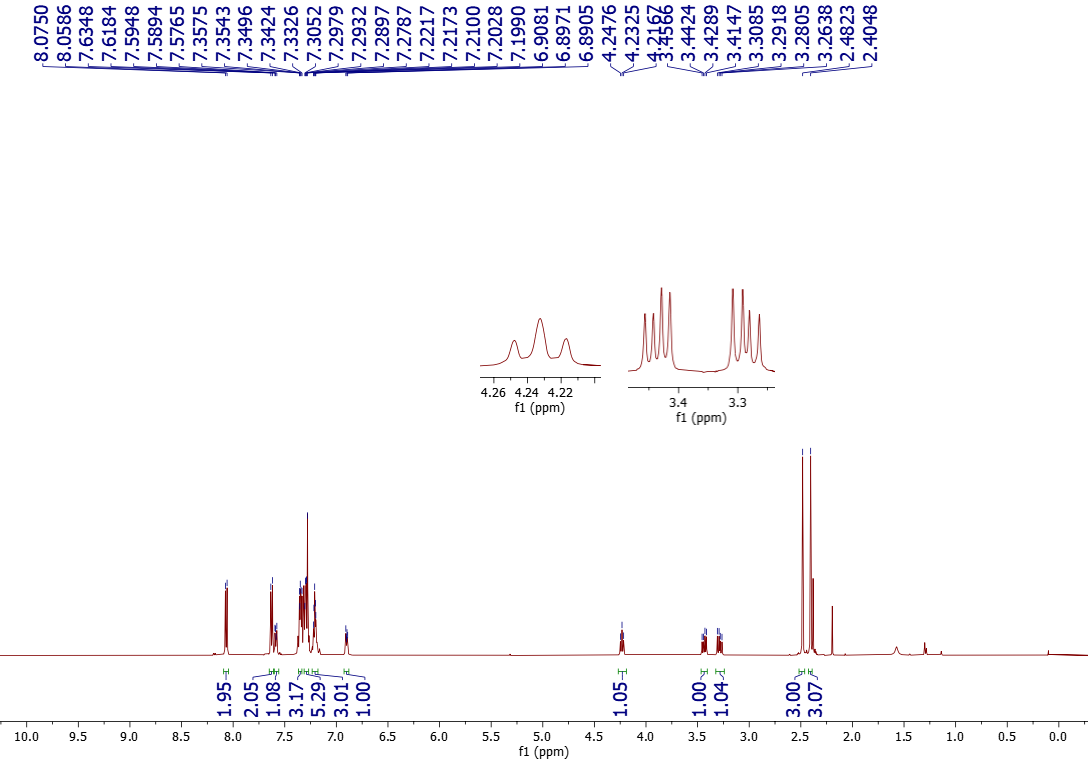


^13^C NMR (125 MHz, CDCl_3_) spectrum of **3i**


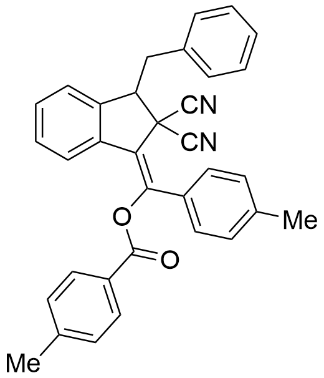

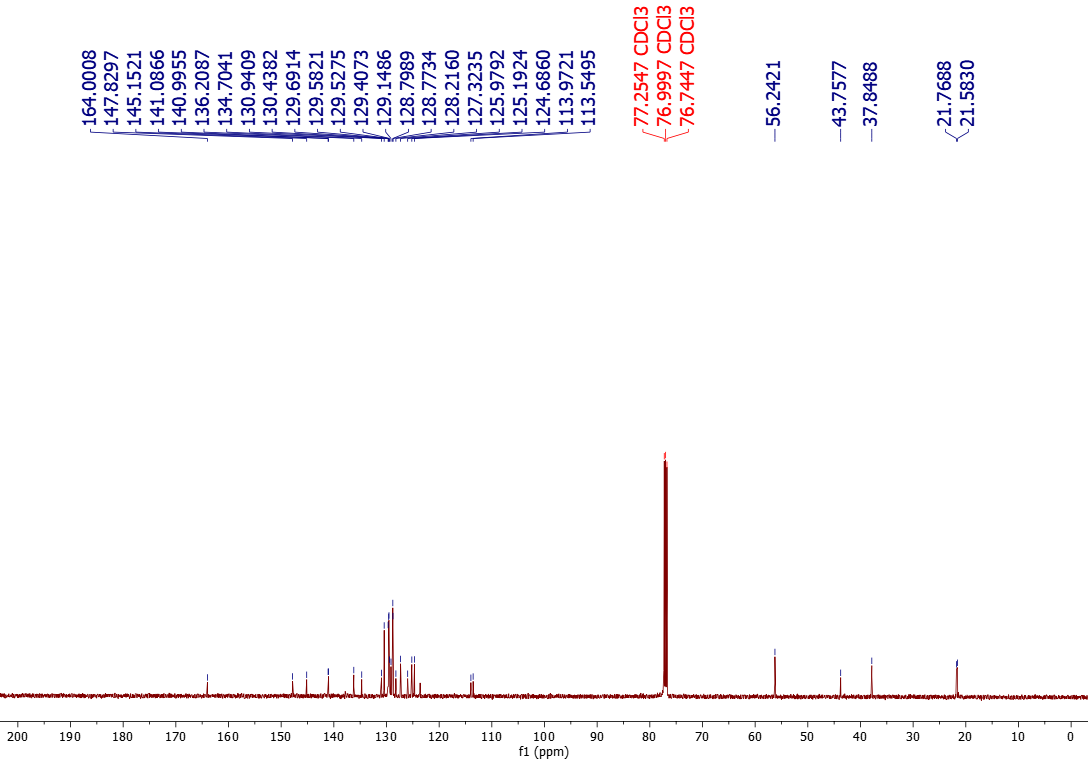


HRMS-ESI Compound **3i**


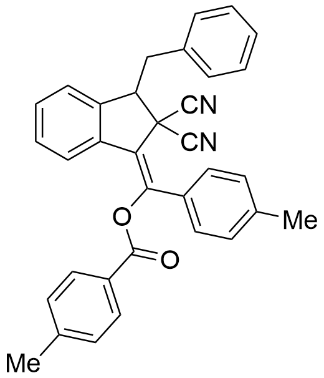

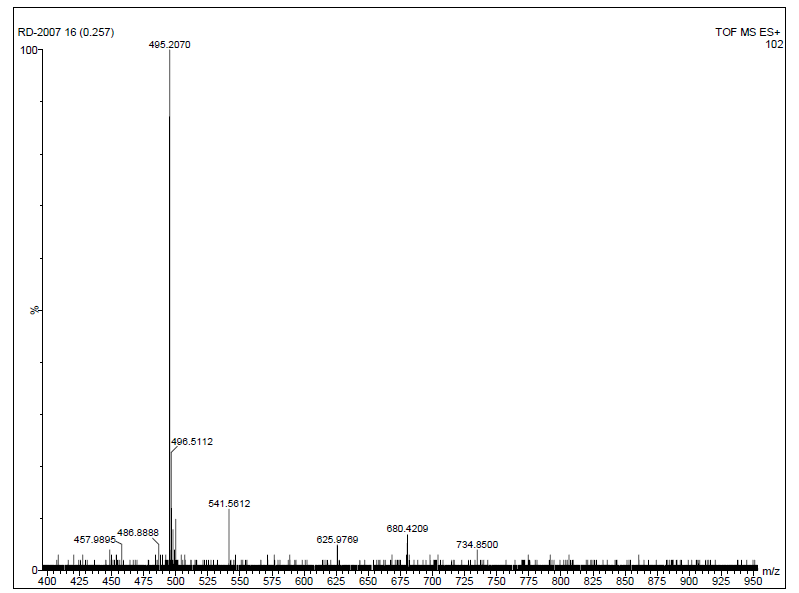


[M+H]^+^

^1^H NMR (300 MHz, CDCl_3_) spectrum of **3j**


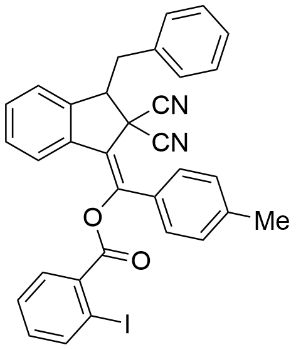

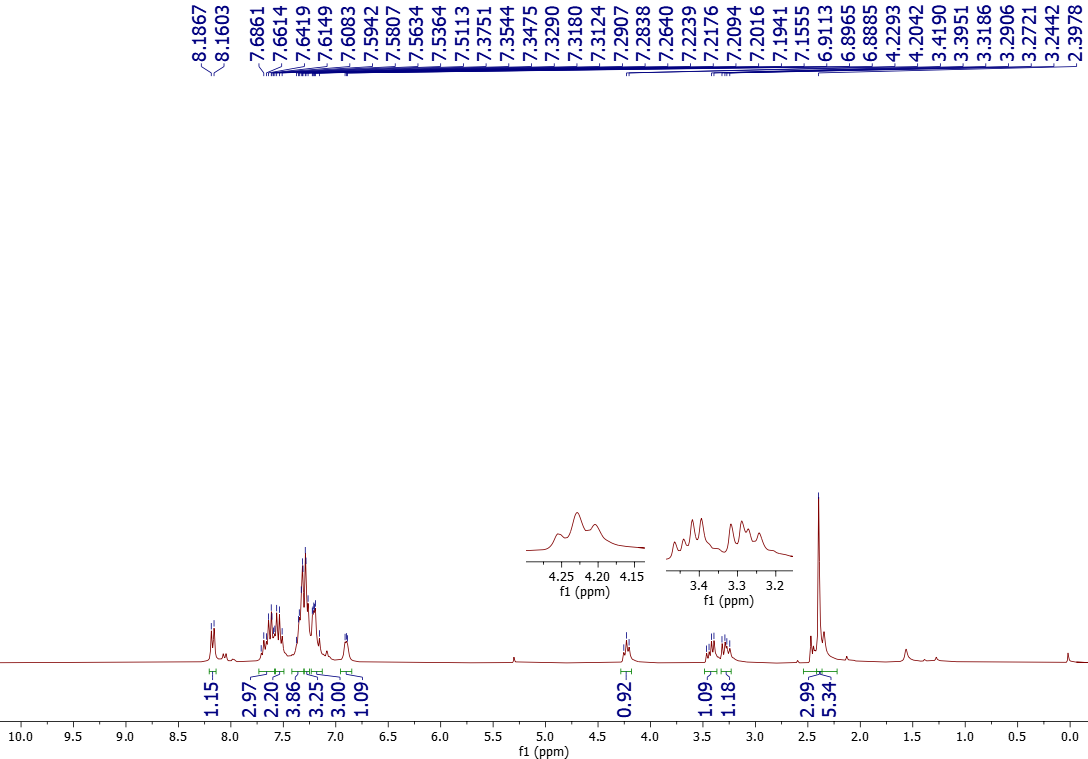


^13^C NMR (125 MHz, CDCl_3_) spectrum of **3j**


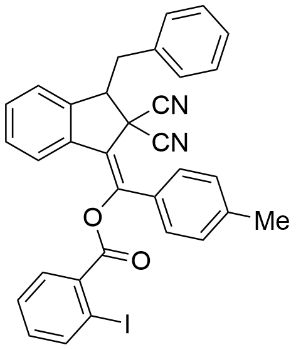

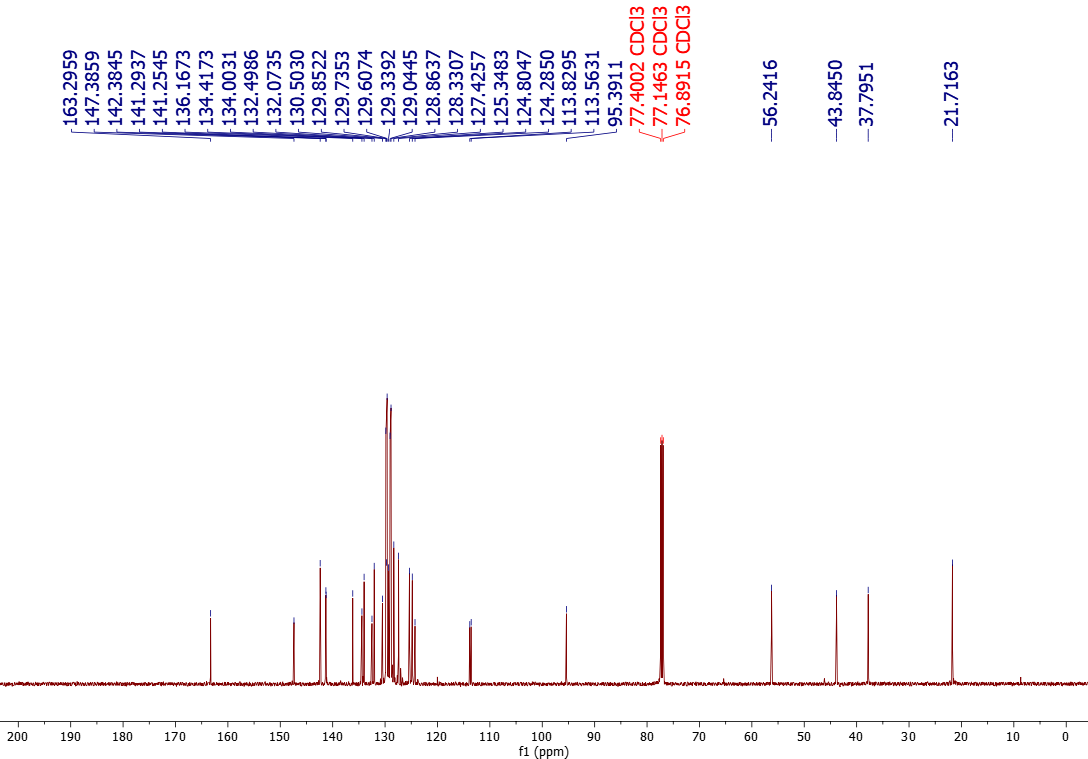


HRMS-ESI Compound **3j**


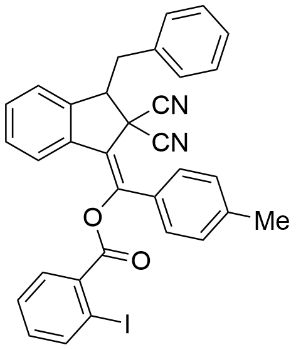

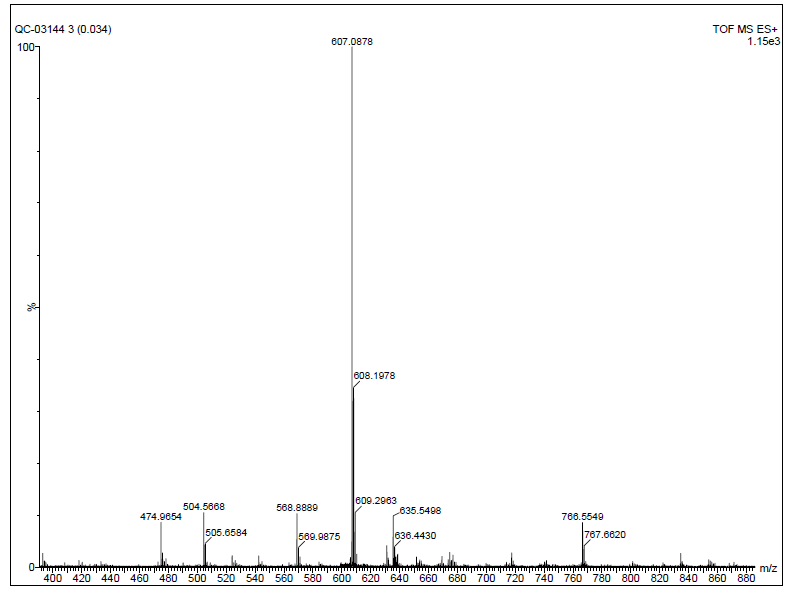


[M+H]^+^

^1^H NMR (400 MHz, CDCl_3_) spectrum of **3k**

**
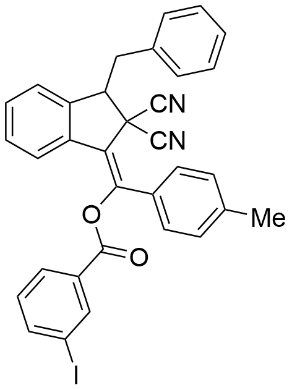
**
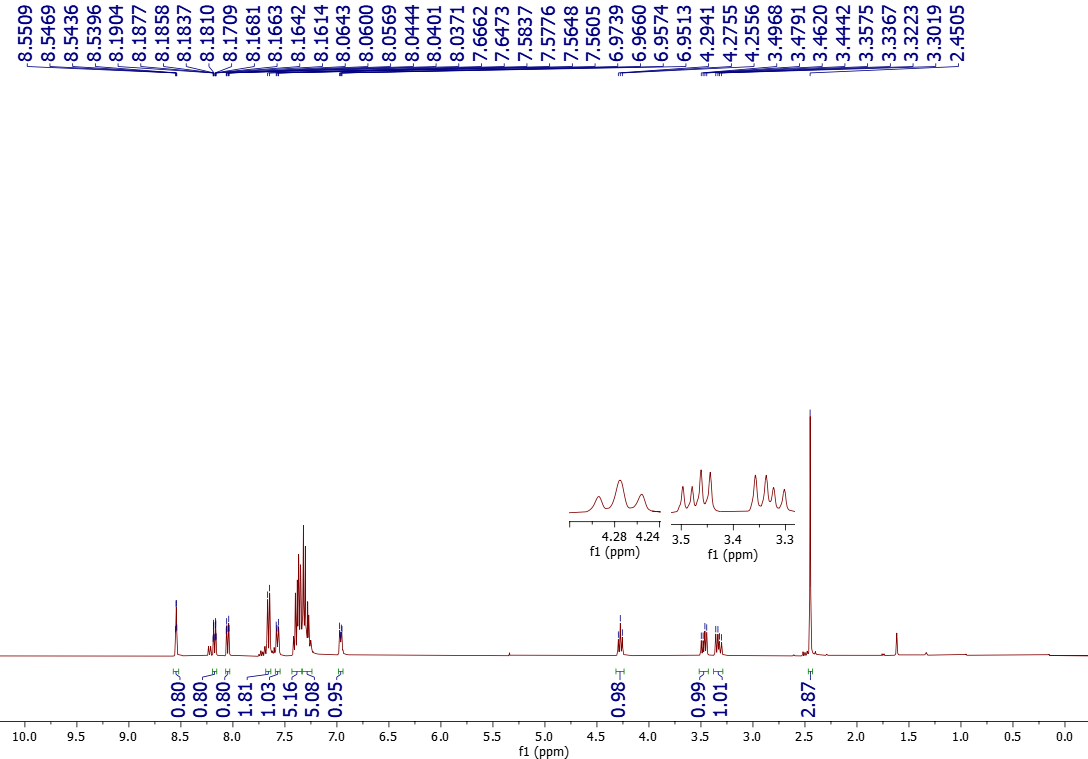


^13^C NMR (100 MHz, CDCl_3_) spectrum of **3k**

**
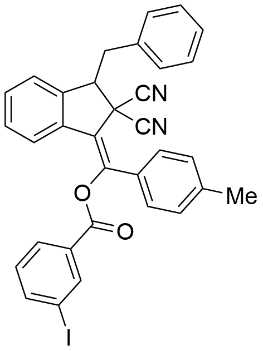
**
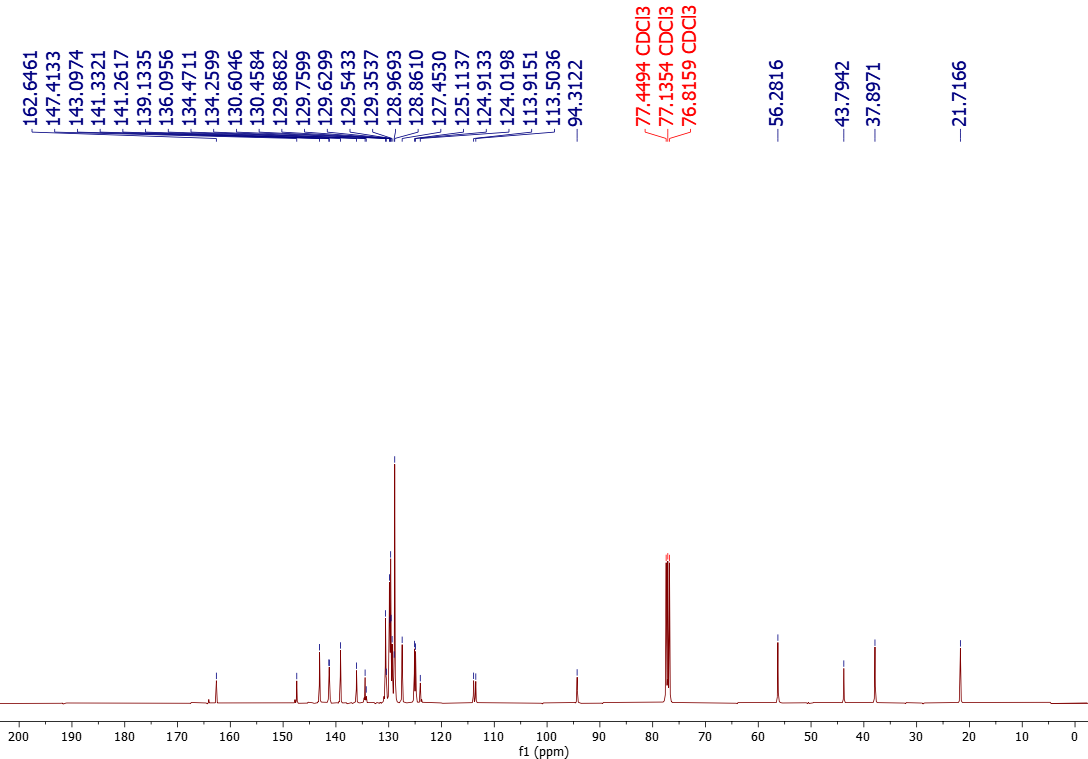


HRMS-ESI Compound **3k**

**
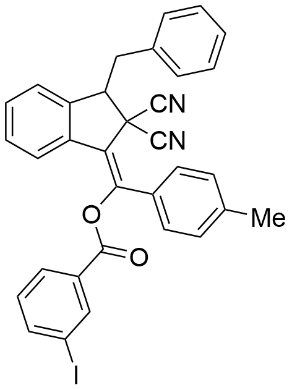
**
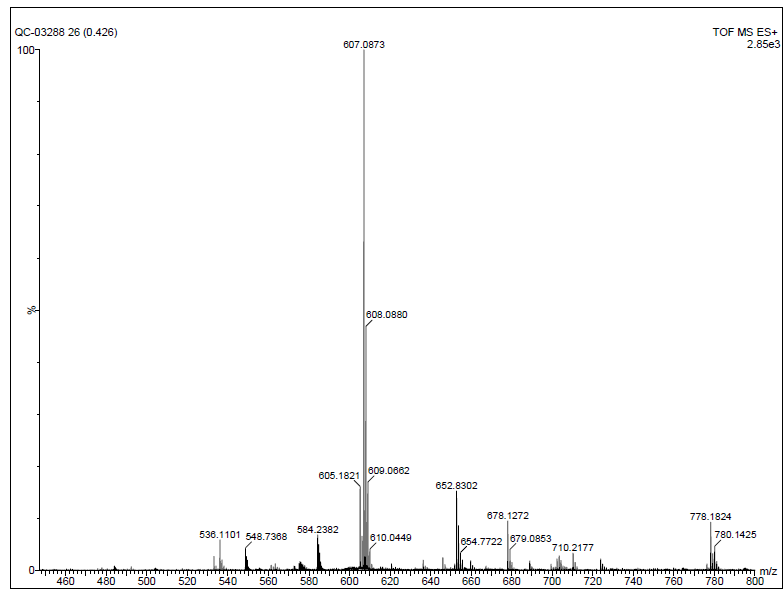


[M+H]^+^

^1^H NMR (300 MHz, CDCl_3_) spectrum of **3l**


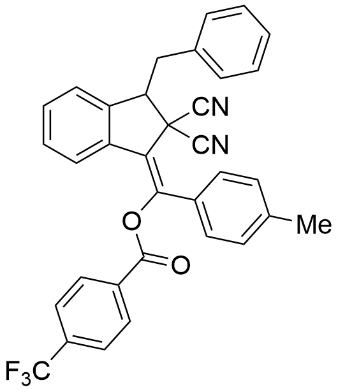

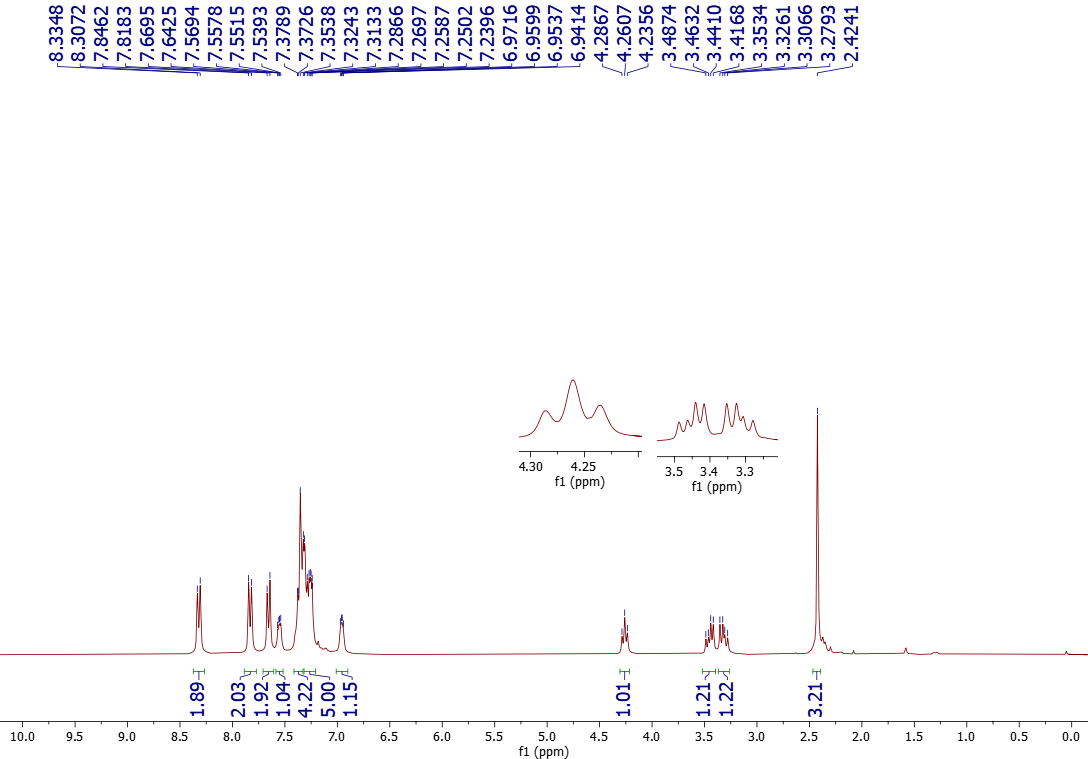


^13^C NMR (75 MHz, CDCl_3_) spectrum of **3l**


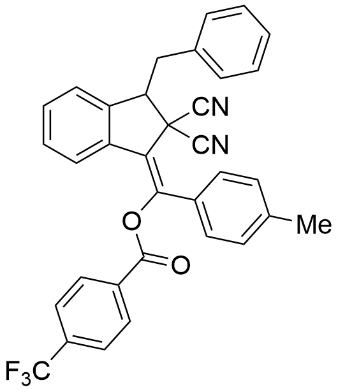

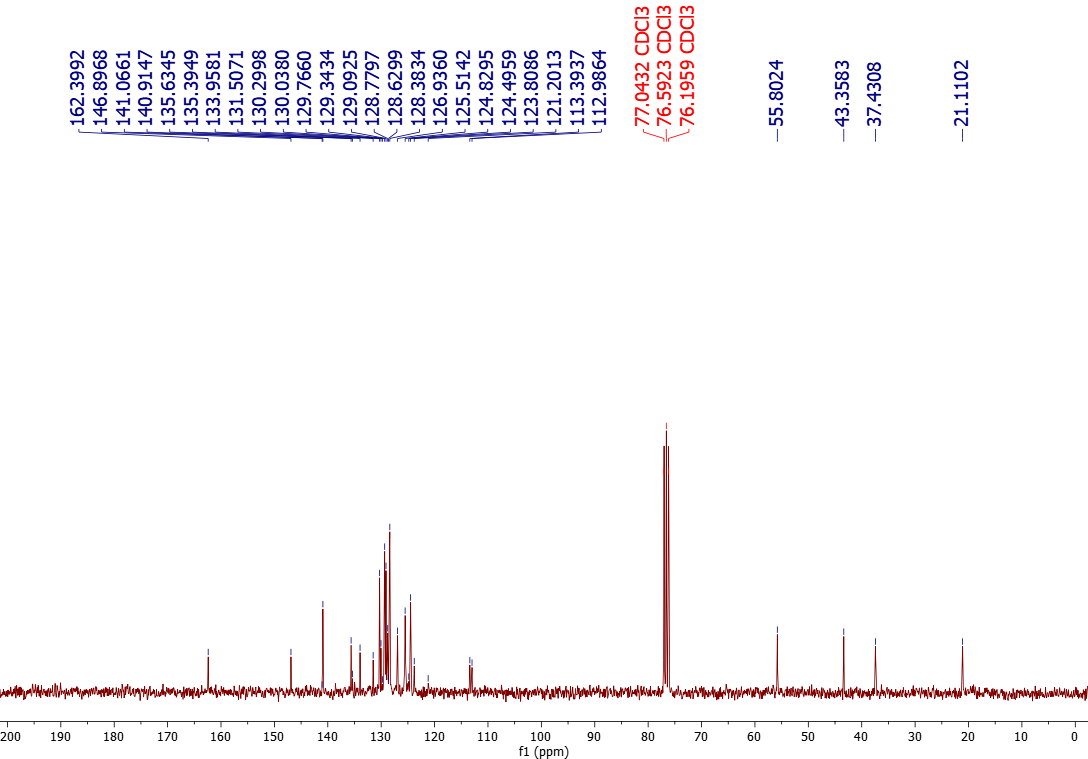


^19^F NMR (471 MHz, CDCl_3_) spectrum of **3l**


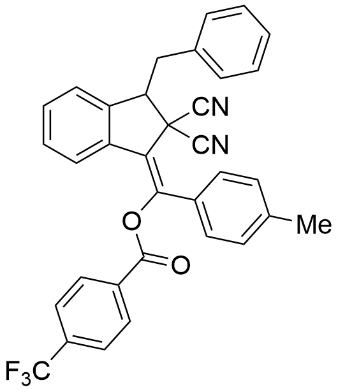
**
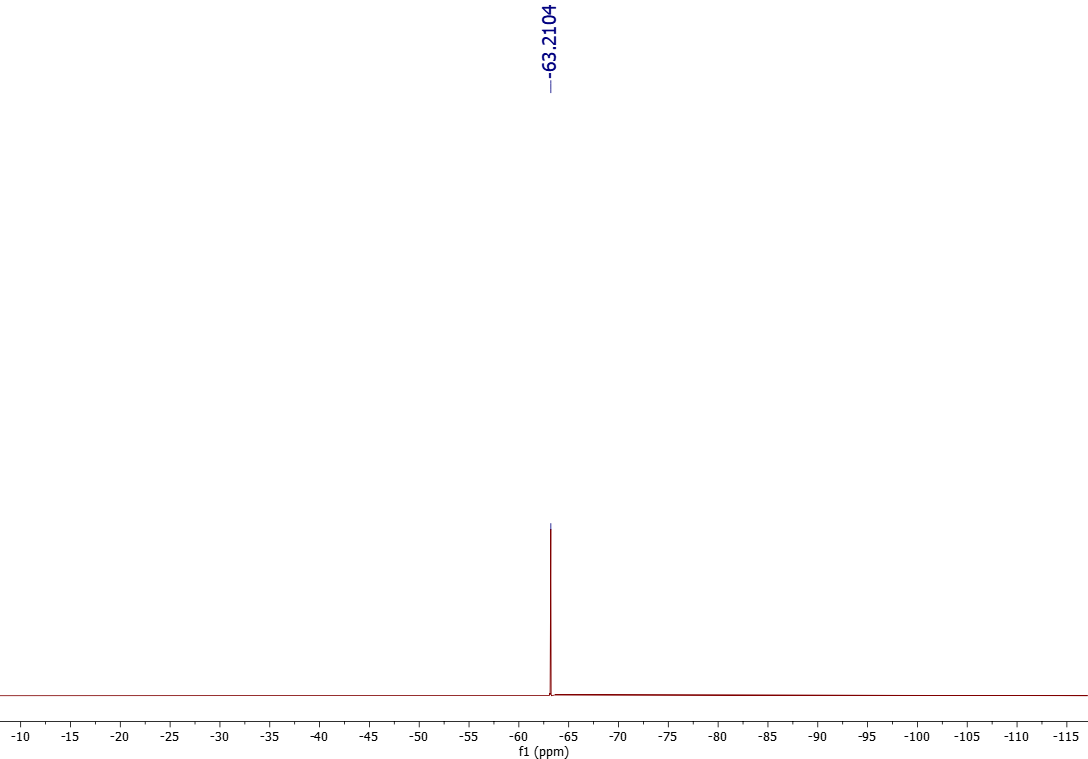
**

HRMS-ESI Compound **3l**


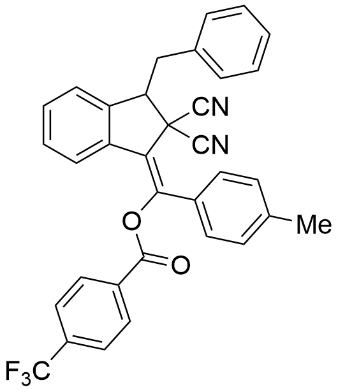

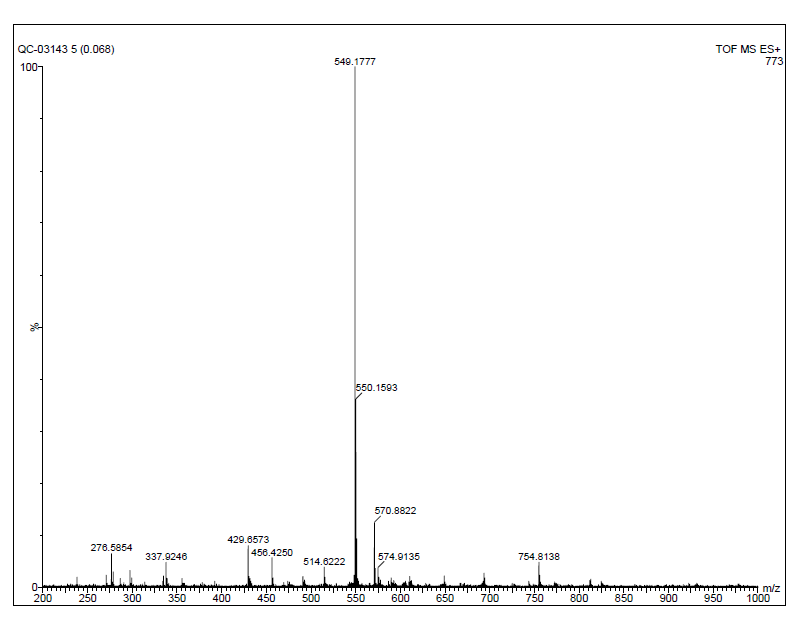


[M+H]^+^

^1^H NMR (400 MHz, CDCl_3_) spectrum of **3m**

**
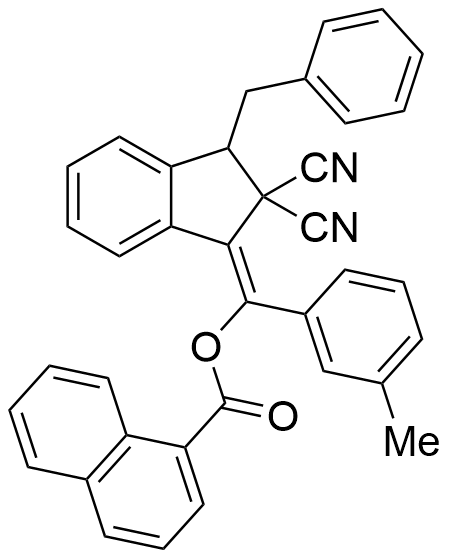

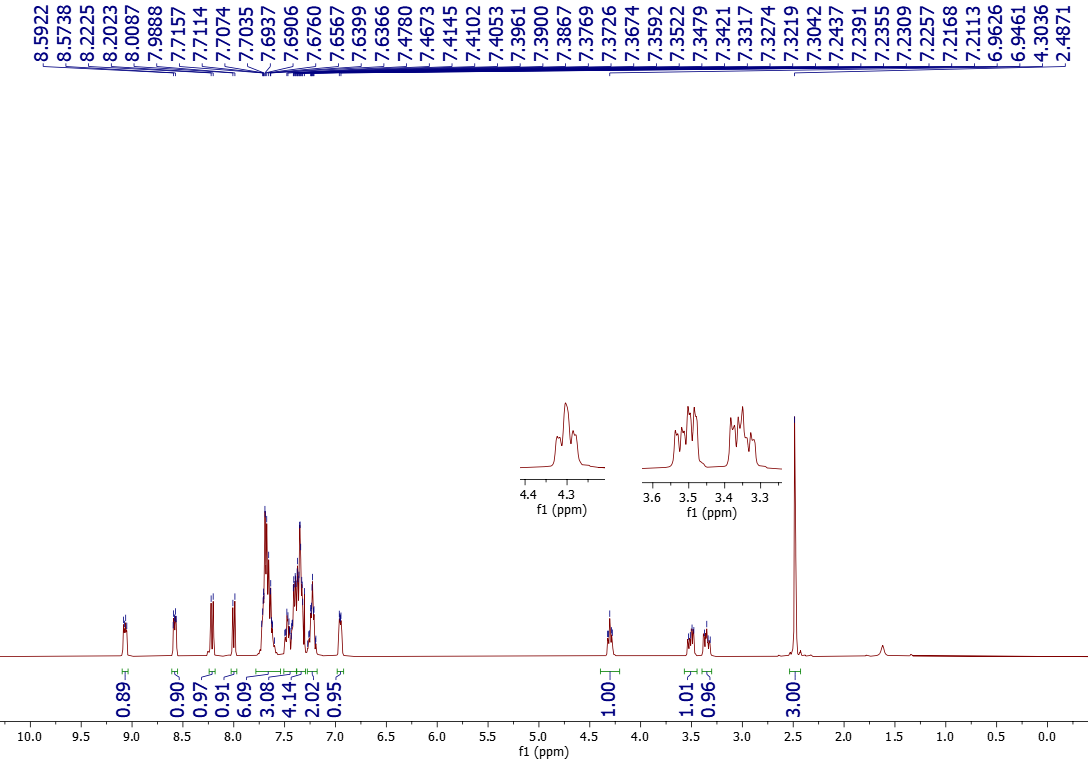
**

^13^C NMR (100 MHz, CDCl_3_) spectrum of **3m**

**
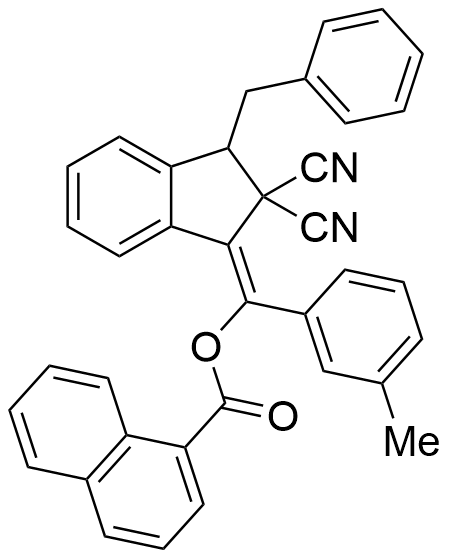

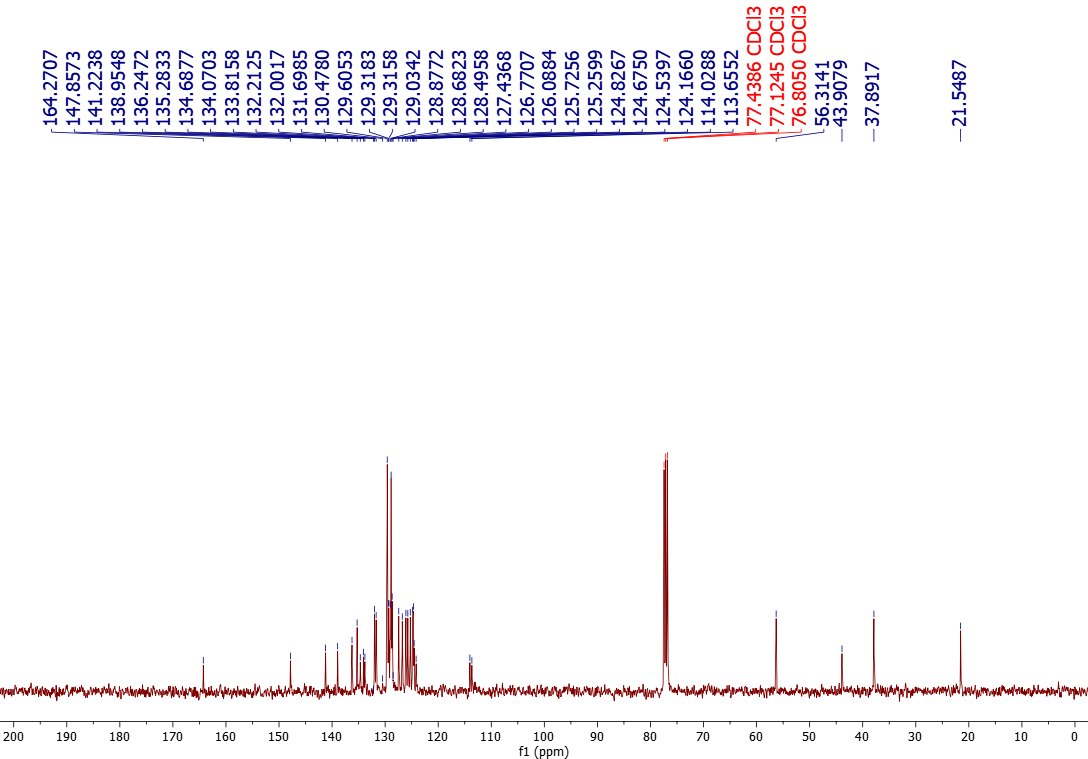
**

HRMS-ESI Compound **3m**

**
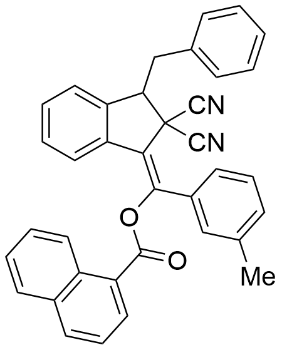
**
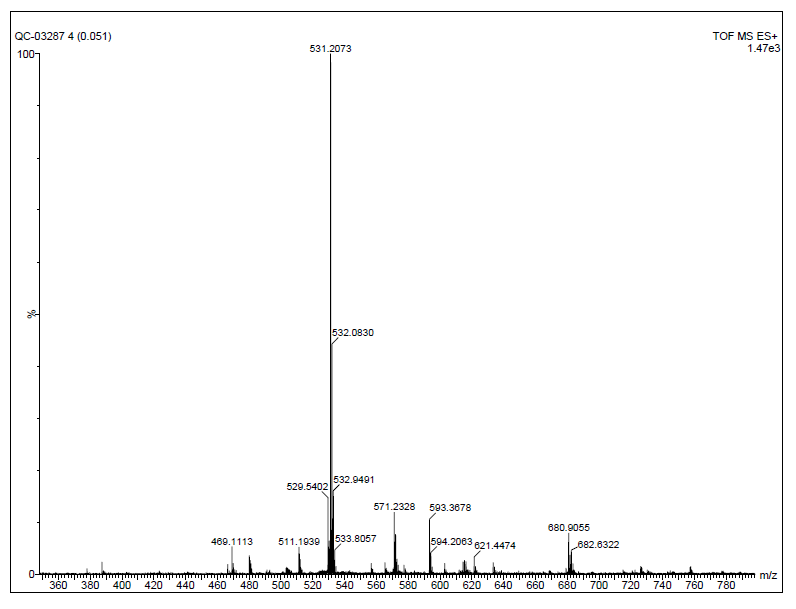


[M+H]^+^

^1^H NMR (400 MHz, CDCl_3_) spectrum of **3n**


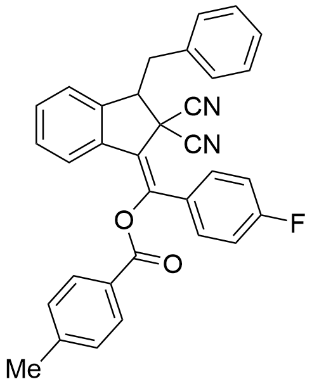

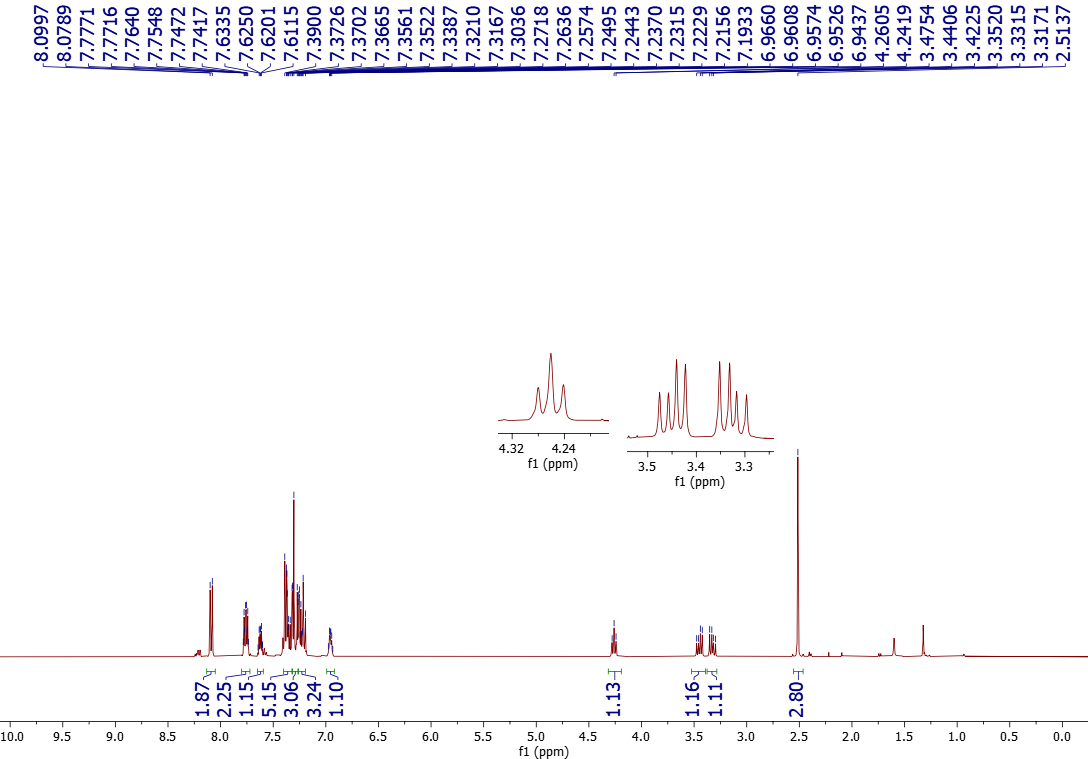


^13^C NMR (100 MHz, CDCl_3_) spectrum of **3n**


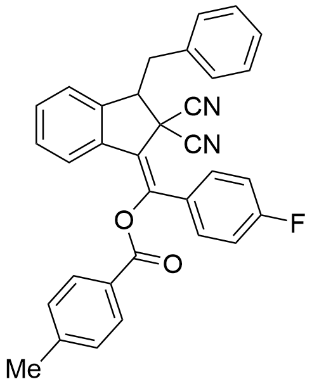

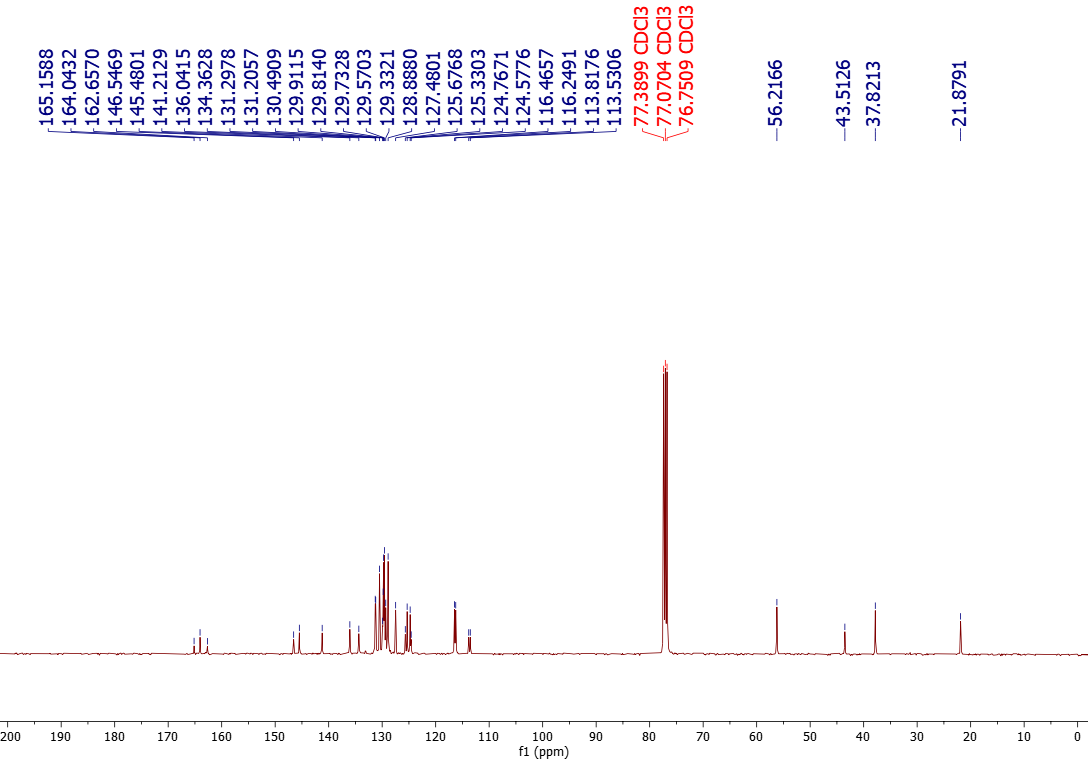


HRMS-ESI Compound **3n**


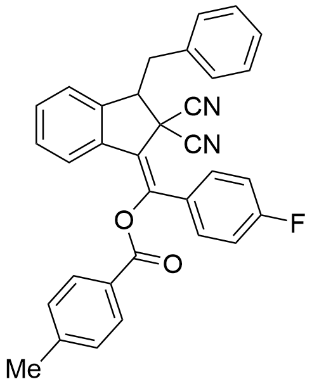

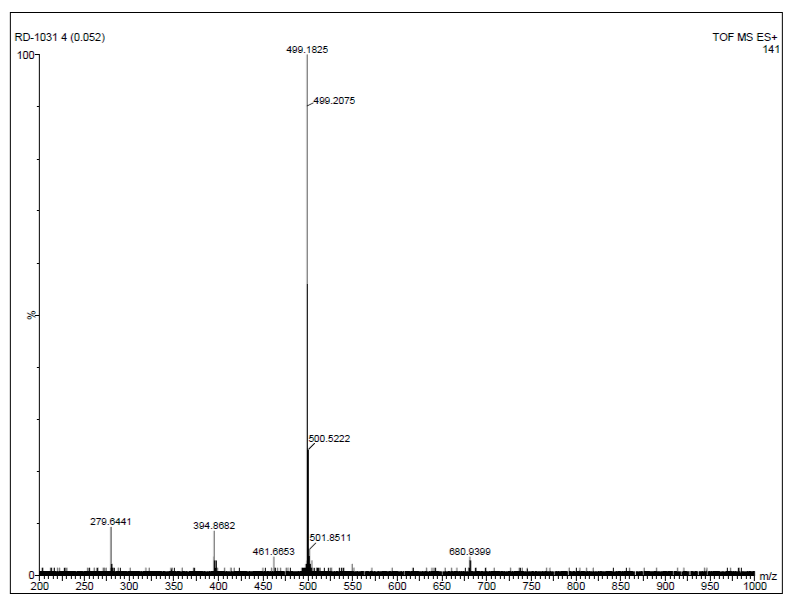


[M+H]^+^

^1^H NMR (400 MHz, CDCl_3_) spectrum of **3o**


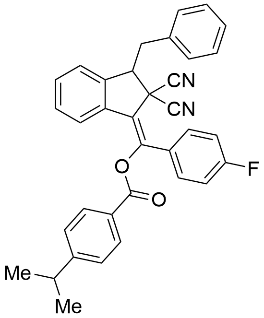
**
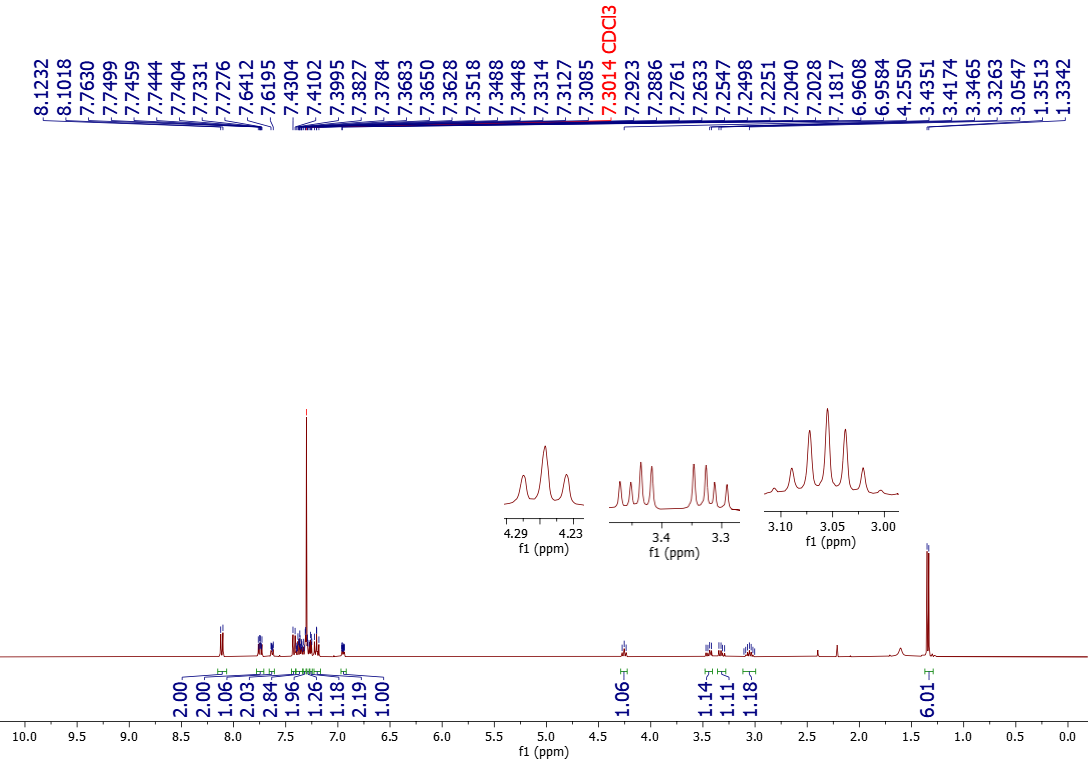
**

^13^C NMR (100 MHz, CDCl_3_) spectrum of **3o**


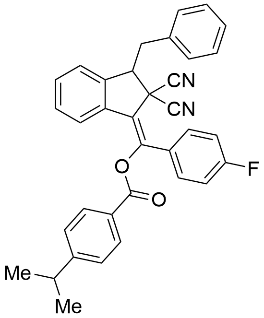

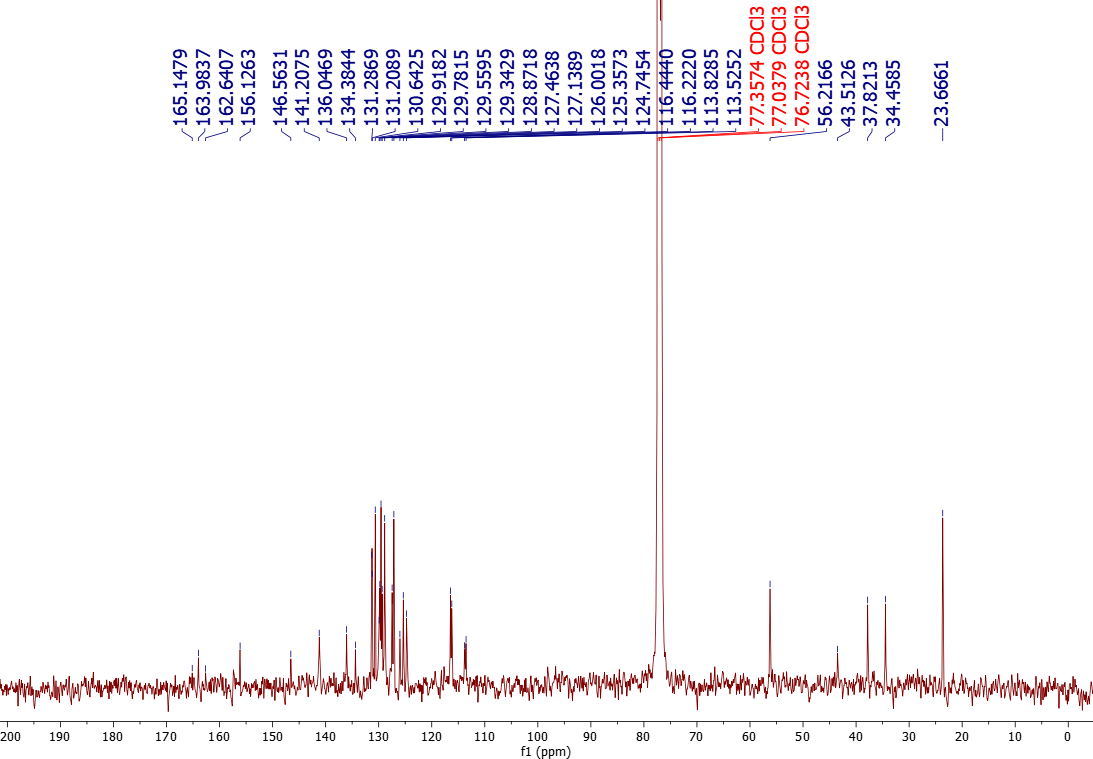


^19^F NMR (471 MHz, CDCl_3_) spectrum of **3o**


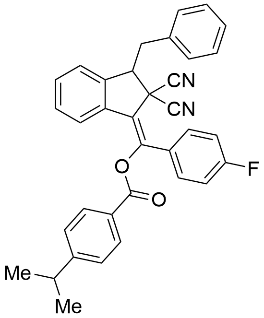

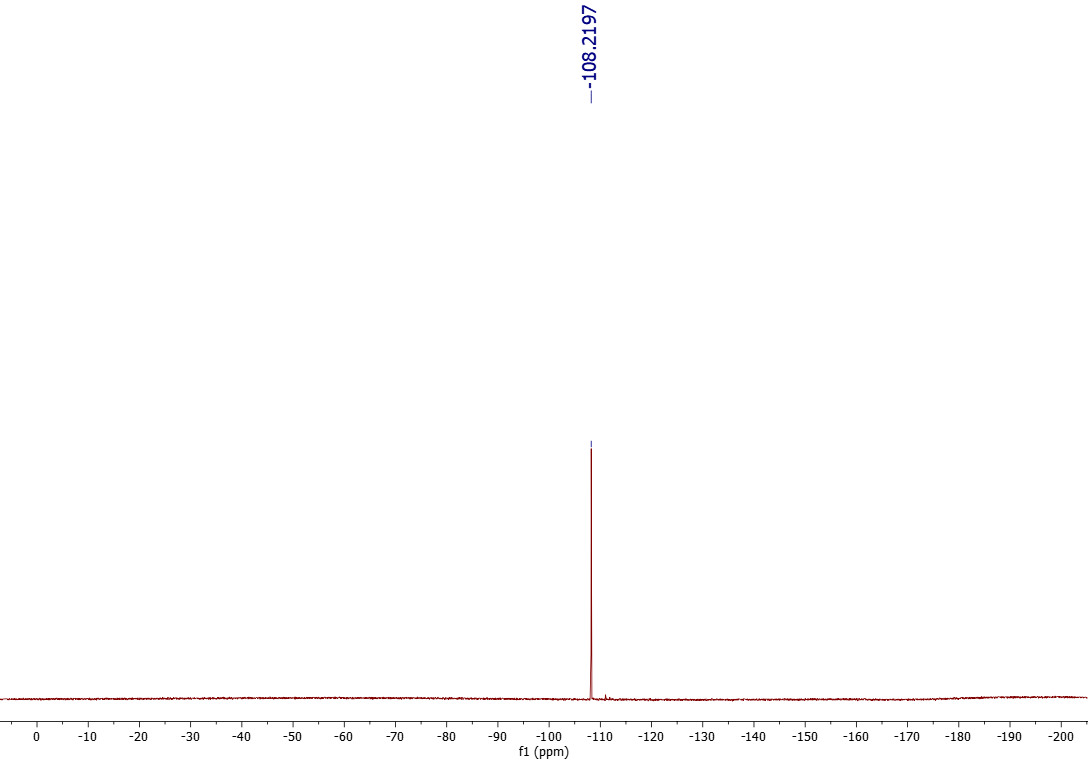


HRMS-ESI Compound **3o**


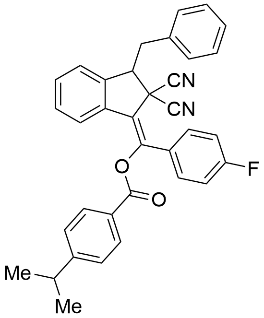

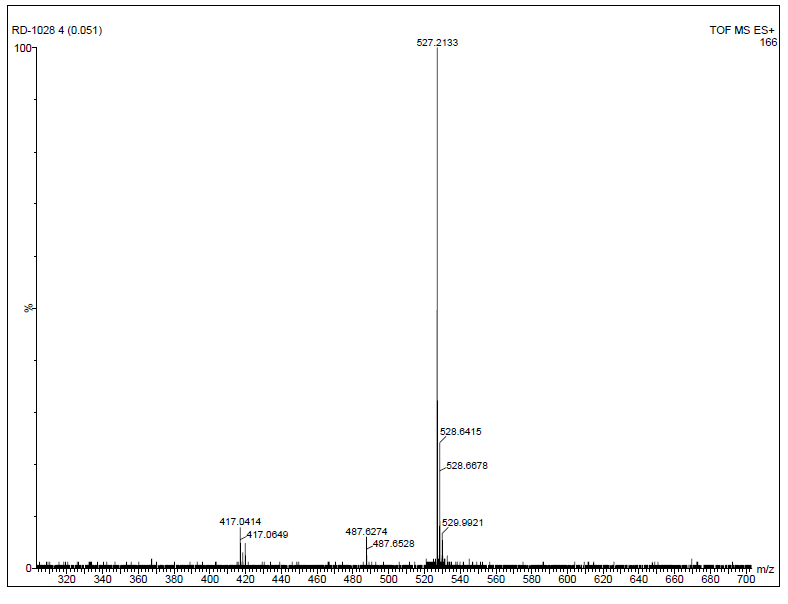


[M+H]^+^

^1^H NMR (500 MHz, CDCl_3_) spectrum of **3p**

**
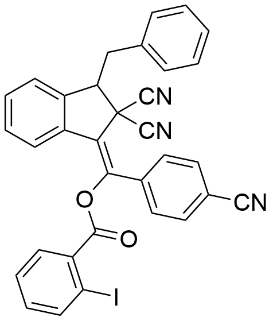
**
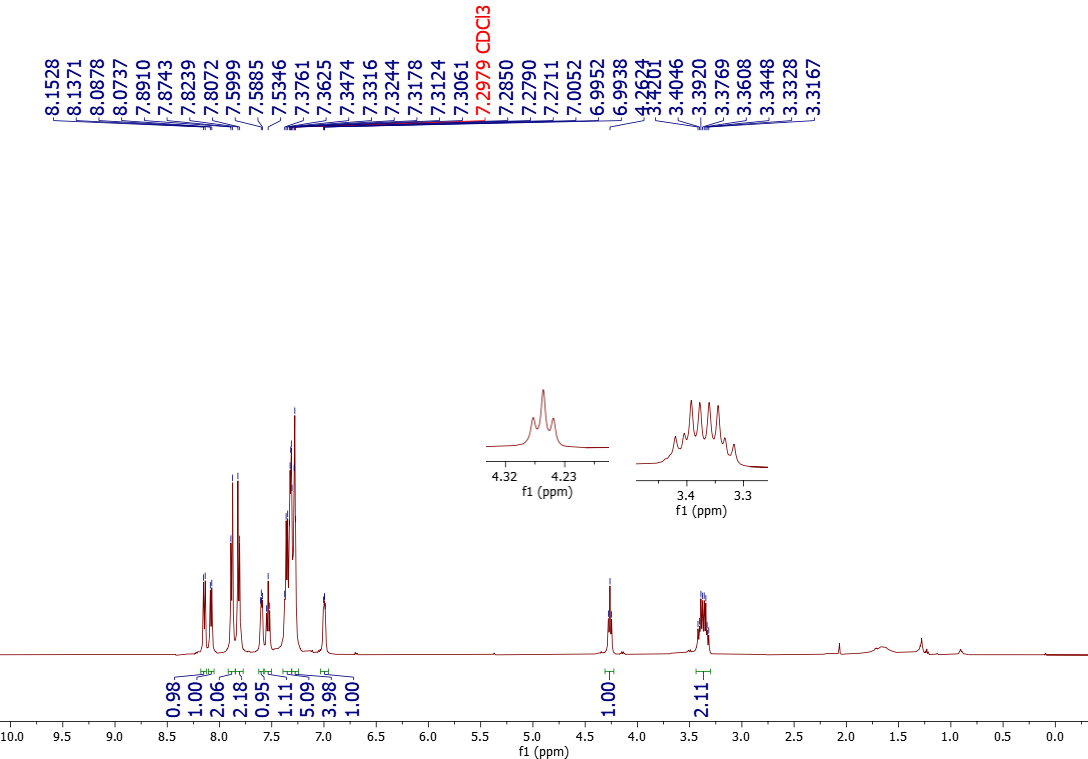


^13^C NMR (125 MHz, CDCl_3_) spectrum of **3p**

**
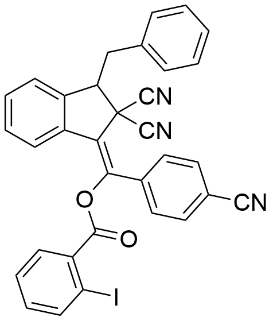
**
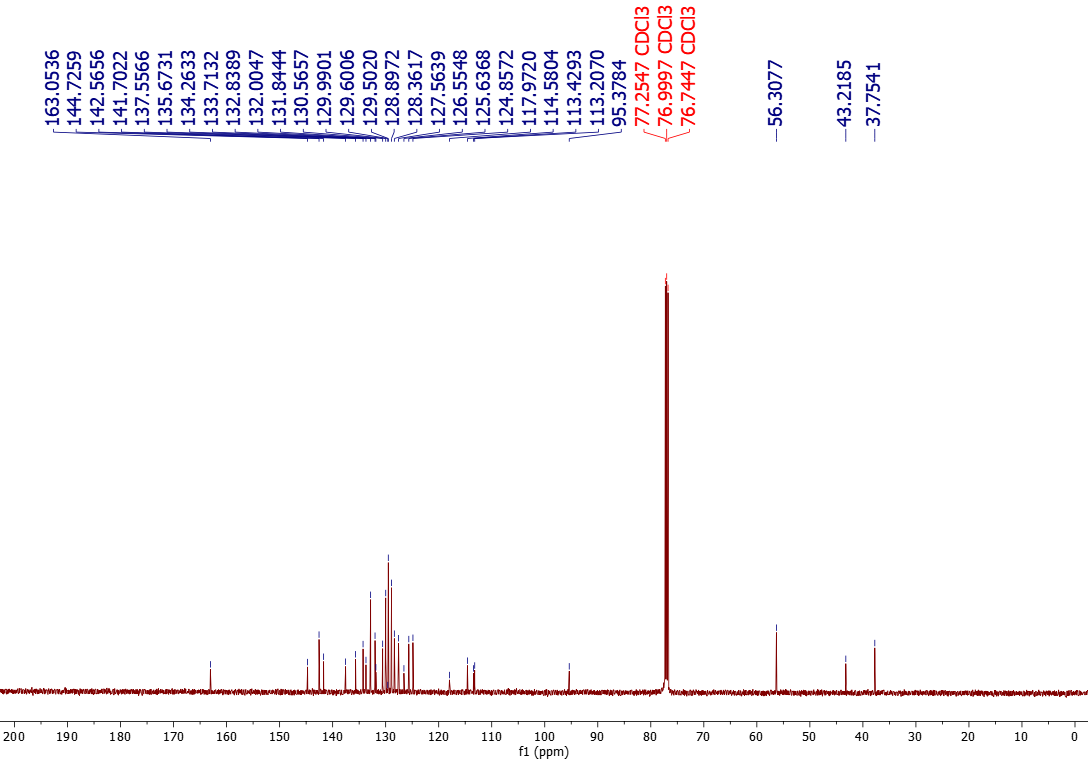


HRMS-ESI Compound **3p**

**
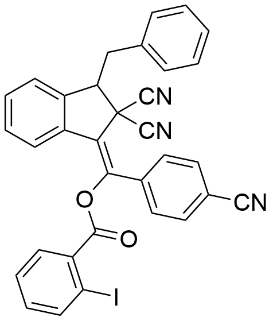
**
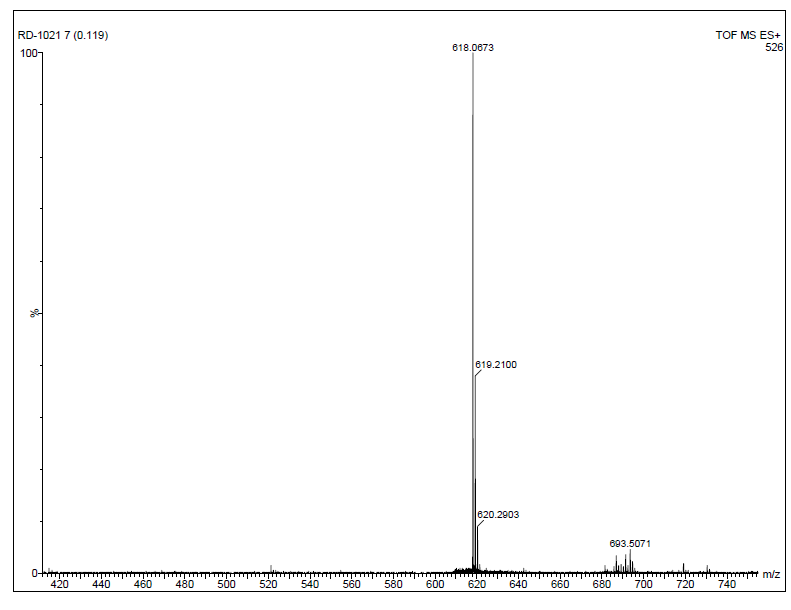


[M+H]^+^

^1^H NMR (400 MHz, CDCl_3_) spectrum of **3q**

^13^C NMR (100 MHz, CDCl_3_) spectrum of **3q**

HRMS-ESI Compound **3q**

[M+H]^+^

^1^H NMR (400 MHz, CDCl_3_) spectrum of **3r**

^13^C NMR (100 MHz, CDCl_3_) spectrum of **3r**

HRMS-ESI Compound **3r**

[M+H]^+^

^1^H NMR (400 MHz, CDCl_3_) spectrum of **3s**

^13^C NMR (100 MHz, CDCl_3_) spectrum of **3s**

HRMS-ESI Compound **3s**

[M+H]^+^

^1^H NMR (400 MHz, CDCl_3_) spectrum of **3t**

^13^C NMR (100 MHz, CDCl_3_) spectrum of **3t**

HRMS-ESI Compound **3t**

[M+H]^+^

^1^H NMR (400 MHz, CDCl_3_) spectrum of **3u**

^13^C NMR (125 MHz, CDCl_3_) spectrum of **3u**

^19^F NMR (471 MHz, CDCl_3_) spectrum of **3u**

HRMS-ESI Compound **3u**

[M+H]^+^

^1^H NMR (300 MHz, CDCl_3_) spectrum of **3v**

^13^C NMR (75 MHz, CDCl_3_) spectrum of **3v**

HRMS-ESI Compound **3v**

[M+H]^+^

^1^H NMR (300 MHz, CDCl_3_) spectrum of **3w**

^13^C NMR (75 MHz, CDCl_3_) spectrum of **3w**

HRMS-ESI Compound **3w**

[M+H]^+^

^1^H NMR (400 MHz, CDCl_3_) spectrum of **3x**

^13^C NMR (100 MHz, CDCl_3_) spectrum of **3x**

HRMS-ESI Compound **3x**

[M+H]^+^

^1^H NMR (500 MHz, CDCl_3_) spectrum of **3y**

^13^C NMR (125 MHz, CDCl_3_) spectrum of **3y**

HRMS-ESI Compound **3y**

[M+H]^+^

^1^H NMR (500 MHz, CDCl_3_) spectrum of **3z**

^13^C NMR (125 MHz, CDCl_3_) spectrum of **3z**

HRMS-ESI Compound **3z**

[M+H]^+^

^1^H NMR (500 MHz, CDCl_3_) spectrum of **3a'**

^13^C NMR (125 MHz, CDCl_3_) spectrum of **3a'**

HRMS-ESI Compound **3a'**

[M+H]^+^

^1^H NMR (500 MHz, CDCl_3_) spectrum of **3b'**

^13^C NMR (125 MHz, CDCl_3_) spectrum of **3b'**

HRMS-ESI Compound **3b'**

[M+H]^+^

^1^H NMR (400 MHz, CDCl_3_) spectrum of **3c'**

^13^C NMR (100 MHz, CDCl_3_) spectrum of **3c'**

HRMS-ESI Compound **3c'**

[M+H]^+^

^1^H NMR (500 MHz, CDCl_3_) spectrum of **3d'**

^13^C NMR (125 MHz, CDCl_3_) spectrum of **3d'**

HRMS-ESI Compound **3d'**

[M+H]^+^

^1^H NMR (500 MHz, CDCl_3_) spectrum of **3e'**

^13^C NMR (125 MHz, CDCl_3_) spectrum of **3e'**

HRMS-ESI Compound **3e'**

[M+H]^+^

**6. Crystal data of 3b**

Table S1: Crystal data and structure refinement for sba207(3b).

Identification code sba207

Empirical formula C_33_H_24_N_2_O_2_

Formula weight 480.54

Temperature 200(2) K

Wavelength 1.54178 Å

Crystal system triclinic

Space group P$\bar{1}$

Z 2

Unit cell dimensions a = 10.1171(3) Å α = 90.686(3) deg.

b = 10.6836(4) Å β = 98.623(3) deg.

c = 11.9744(4) Å γ = 94.796(3) deg.

Volume 1274.77(8) Å^3^

Density (calculated) 1.25 g/cm^3^

Absorption coefficient 0.62 mm^-1^

Crystal shape brick

Crystal size 0.144 x 0.105 x 0.051 mm^3^

Crystal colour colourless

Theta range for data collection 3.7 to 68.5 deg.

Index ranges -12≤h≤9, -12≤k≤12, -10≤l≤14

Reflections collected 16573

Independent reflections 4574 (R(int) = 0.0189)

Observed reflections 3652 (I > 2σ(I))

Absorption correction Semi-empirical from equivalents

Max. and min. transmission 0.96 and 0.86

Refinement method Full-matrix least-squares on F^2^

Data/restraints/parameters 4574 / 0 / 336

Goodness-of-fit on F^2^ 0.96

Final R indices (I>2sigma(I)) R1 = 0.037, wR2 = 0.114

Largest diff. peak and hole 0.14 and -0.20 eÅ^-3^

sba207 (3b): colourless crystal (brick), dimensions 0.144 x 0.105 x 0.051 mm^3^, crystal system triclinic, space group P$\bar{1}$, Z=2, a=10.1171(3) Å, b=10.6836(4) Å, c=11.9744(4) Å, alpha=90.686(3) deg, beta=98.623(3) deg, gamma=94.796(3) deg, V=1274.77(8) Å^3^, rho=1.252 g/cm^3^, T=200(2) K, Theta_max_= 68.536 deg, radiation CuKα, lambda=1.54178 Å, 0.5 deg omega-scans with CCD area detector, covering the asymmetric unit in reciprocal space with a mean redundancy of 3.52and a completeness of 97.2% to a resolution of 0.83 Å, 16573 reflections measured, 4574 unique (R(int)=0.0189), 3652 observed (I > 2σ(I)), intensities were corrected for Lorentz and polarization effects, an empirical scaling and absorption correction was applied using X-Area LANA 2.7.5.0 (STOE, 2022) based on the Laue symmetry of the reciprocal space, mu=0.62mm^-1^, T_min_=0.86, T_max_=0.96, structure solved with SHELXT-2014 (Sheldrick 2014)^[2]^ and refined against F^2^ with a Full-matrix least-squares algorithm using the SHELXL-2018/3 (Sheldrick, 2018) software^[3]^, 336 parameters refined, hydrogen atoms were treated using appropriate riding models, goodness of fit 0.96 for observed reflections, final residual values R1(F)=0.037, wR(F^2^)=0.114 for observed reflections, residual electron density -0.20 to 0.14 eÅ^-3^. CCDC **2490672** contains the supplementary crystallographic data for this paper. The data can be obtained free of charge from The Cambridge Crystallographic Data Centre via www.ccdc.cam.ac.uk/structures.

Lit. 2: (SHELXT - Integrated space-group and crystal structure determination)

Sheldrick G. M., Acta Cryst. A71 (2015) 3-8.

Lit. 3: (program SHELXL-2018/3 (Sheldrick, 2018) for structure refinement)

Sheldrick G. M., Acta Cryst. (2015). C71, 3-8

Lit. APEX, APEX2, SMART, SAINT, SAINT-Plus:

Bruker (2007). "Program name(s)". Bruker AXS Inc., Madison, Wisconsin, USA.

**7.Computational Data**

Gaussian 16 ^2^ was used to fully optimize all the structures reported in this study at the B3LYP level of theory.^3^ For all the calculations, solvent effects were considered using the SMD solvation model^4^ with toluene as the solvent. Grimme empirical dispersion was added with the GD3 term on all the calculations.^5^ For geometry optimizations, the def2-SVP basis set was used for all atoms, with the def2-ECP applied only for iodine. This basis set combination will be referred to as BS1. Frequency calculations were carried out at the same level of theory as those for structural optimization. Transition structures were located using the Berny algorithm. Intrinsic reaction coordinate (IRC) calculations were used to confirm the connectivity between transition structures and minima.^6^ To enhance the precision of the energies derived from the SMD/B3LYP-D3/def2-SVP calculations, we performed single-point energy calculations for all structures using the B3LYP functional method with the GD3 term and a larger basis set (BS2), with consideration of the SMD solvation model. This expanded basis set employed def2-SVP for all atoms, with the def2-ECP applied only to iodine. The overall solvation free energy values reported in this work were obtained by adding the free energy corrections from the frequency analysis to the single-point solvation free energies calculated using the SMD solvation model. Moreover, the overall free energy values were corrected by adding ∆G^1atm→1M^ = 1.89 kcal/mol to account for the free-energy change associated with compressing 1 mol of an ideal gas from 1 atm to the 1 M solution phase standard state.

**Figure S1.** Optimized structures of cis- and trans-Cu(II)(OH)I complexes reacting with one or two equivalents of 4-methylbenzoic acid. Relative free energies are given in kcal/mol. Selected bond distances (Å) are shown in blue.

**Table S2.** Calculated singlet–triplet energy gaps (ΔG_s-t_, kcal/mol) for alkenes with different substituents R¹ and R². The reduced gaps observed for alkenes bearing strongly π-accepting groups (CN, NO₂, CO₂Me) rationalize the enhanced reactivity of these enynes toward benzyl radical addition.

|  | |
| --- | --- |
| alkenes | ∆G(kcal.mol^-1^) |
| R^1^, R^2^=H, H | 77.8 |
| R^1^, R^2^=H, CN | 54.2 |
| R^1^, R^2^=CN, CN | 49.5 |
| R^1^, R^2^=NO_2_, NO_2_ | 49.8 |
| R^1^, R^2^=CN, NO_2_ | 52.6 |
| R^1^, R^2^=CN, CO_2_Me | 51.2 |

**Table S3.** Cartesian coordinates along with total potential energies (E), enthalpies (H), and Gibbs free energies (G) of all structures are reported in atomic units (a.u.)

1a

E (SMD/B3LYP-D3/def2-SVP) = -800.79677

H (SMD/B3LYP-D3/def2-SVP) = -800.556495

G (SMD/B3LYP-D3/def2-SVP) = -800.620674

E (SMD/B3LYP-D3/def2-TZVP//SMD/B3LYP-D3/def2-SVP) = -801.670182

C -0.98203100 -4.92092000 -0.33765300

C 2.05341500 -4.58860300 -0.39141000

C 1.31663900 -5.22488600 -1.43329000

C 2.03583400 -5.76298000 -2.52343500

C 1.39739300 -6.48003700 -3.53103800

C 0.01754500 -6.70966300 -3.46282800

C -0.70895400 -6.20379500 -2.39045700

C -0.09730000 -5.42364300 -1.38386700

H 3.11664100 -5.61366100 -2.55643300

H 1.98092800 -6.87839200 -4.36460800

H -0.48661700 -7.28844400 -4.23996600

H -1.78440800 -6.38975300 -2.33149800

C 2.74107900 -4.07129700 0.47247700

C 3.52494400 -3.43938900 1.48818300

C 3.30561800 -2.08446000 1.81984100

C 4.52814400 -4.16164800 2.16979800

C 4.07182600 -1.47608900 2.81402800

H 2.53703600 -1.52273800 1.28587600

C 5.28730100 -3.54244500 3.16254600

H 4.70108100 -5.20957000 1.91486900

C 5.06168400 -2.20018300 3.48807500

H 3.89540900 -0.42658000 3.06303800

H 6.06034000 -4.11085100 3.68604600

H 5.65881300 -1.71797300 4.26634800

C -0.99616100 -3.71585000 0.30784400

H -1.80105900 -5.59444600 -0.06456100

C -0.15109700 -2.59850600 0.00308600

C -1.98534600 -3.47112800 1.32275500

N 0.48307700 -1.65566500 -0.23239600

N -2.78296200 -3.27476200 2.14262100

2

E (SMD/B3LYP-D3/def2-SVP) = -459.835499

H (SMD/B3LYP-D3/def2-SVP) = -459.682643

G (SMD/B3LYP-D3/def2-SVP) = -459.727741

E (SMD/B3LYP-D3/def2-TZVP//SMD/B3LYP-D3/def2-SVP) = -460.350564

C -0.54170500 -0.92701800 -0.13826800

C 0.82707900 -0.86328900 0.17373100

C 1.47502800 0.36597900 0.24135100

C 0.78064400 1.56668200 0.00063200

C -0.58649800 1.49177300 -0.31012400

C -1.24465300 0.26287800 -0.38011400

H 1.36697100 -1.79376500 0.36067900

H 2.54078500 0.40266600 0.48505500

H -1.14537100 2.41211600 -0.50069300

H -2.30752800 0.22332500 -0.62285200

C 1.50053700 2.88795500 0.07621500

H 1.95964900 3.03187000 1.06886800

H 2.31869800 2.93399000 -0.66260000

H 0.82541800 3.73569700 -0.11243300

C -1.19151200 -2.26451600 -0.19922100

O -0.62540100 -3.31662800 0.00284800

O -2.50281100 -2.20379000 -0.50785000

H -2.83309400 -3.11905600 -0.52292100

toluene

E (SMD/B3LYP-D3/def2-SVP) = -271.389300

H (SMD/B3LYP-D3/def2-SVP) = -271.255453

G (SMD/B3LYP-D3/def2-SVP) = -271.290636

E (SMD/B3LYP-D3/def2-TZVP//SMD/B3LYP-D3/def2-SVP) = -271.684084

C -2.39828600 -0.49389000 -0.00373500

C -0.99972600 -0.50152900 0.00483100

C -0.30661500 0.71306400 -0.00400000

C -1.00767600 1.92277400 -0.02047700

C -2.41224200 1.94640700 -0.02696800

C -3.09464800 0.71831000 -0.02021100

H -2.95109900 -1.43742600 -0.00099400

H -0.45327600 -1.44831900 0.01475200

H 0.78694100 0.71948800 -0.00147200

H -0.45545300 2.86737800 -0.03002700

H -4.18887400 0.71312500 -0.02959700

C -3.16737000 3.25270200 -0.00866800

H -4.13758300 3.16807800 -0.52268000

H -3.37503300 3.57299300 1.02770400

H -2.59443500 4.06046200 -0.49000700

3b

E (SMD/B3LYP-D3/def2-SVP) = -1530.884119

H (SMD/B3LYP-D3/def2-SVP) = -1530.372332

G (SMD/B3LYP-D3/def2-SVP) = -1530.472205

E (SMD/B3LYP-D3/def2-TZVP//SMD/B3LYP-D3/def2-SVP) = -1532.545274

C 3.56963400 -1.67631200 2.35254900

C 1.80783500 -2.31657400 0.79110500

C 2.98524800 -1.86264600 0.03603300

C 3.17739100 -1.74500700 -1.34837600

C 4.42951900 -1.34519500 -1.82229500

C 5.47759300 -1.06393800 -0.93488200

C 5.28641200 -1.17526700 0.44771300

C 4.03995000 -1.57711600 0.92133400

H 2.37179000 -1.97652100 -2.04542300

H 4.59365100 -1.25869400 -2.89934900

H 6.45071800 -0.75418400 -1.32379900

H 6.09477000 -0.94144100 1.14327900

C 0.51260800 -2.28485000 0.40004300

C -0.66270000 -2.87137700 1.06418200

C -0.61044700 -4.15745600 1.63223500

C -1.87287500 -2.15260100 1.11023000

C -1.73185400 -4.69682000 2.26393500

H 0.30670700 -4.74613000 1.56386800

C -2.98985700 -2.69435800 1.74390700

H -1.92944400 -1.15840100 0.66218700

C -2.92168000 -3.96598600 2.32603500

H -1.67632400 -5.69616000 2.70230800

H -3.91927900 -2.12109000 1.78742200

H -3.79914500 -4.38872500 2.82195500

C 2.33560200 -2.67772100 2.20169900

H 4.32471200 -2.10538800 3.02633900

C 1.37889000 -2.51563800 3.30931200

C 2.83262200 -4.07038200 2.22009000

N 0.67614600 -2.36329700 4.21558400

N 3.21862700 -5.16192700 2.21631000

C 6.26307900 2.63863400 2.63483300

C 6.26137500 1.69888800 3.67169900

C 5.22671200 0.76400000 3.76776100

C 4.18116900 0.75012700 2.83060400

C 4.19217600 1.69883100 1.79626400

C 5.22324500 2.63794100 1.69980300

H 7.07055100 3.37180100 2.55947100

H 7.06697300 1.69712100 4.41099200

H 5.22899600 0.03418300 4.58322000

H 3.38552600 1.69157200 1.05839900

H 5.21550300 3.37028600 0.88801300

C 3.09857800 -0.29960900 2.89889900

H 2.76775500 -0.42927400 3.94045800

H 2.22629700 0.02383000 2.30939600

C 0.03878700 0.23243600 -2.23177600

C -0.44239100 -0.60422600 -3.25504600

C -0.73507100 -0.07339800 -4.50827400

C -0.55966200 1.29648800 -4.77903800

C -0.08028500 2.12199700 -3.74807800

C 0.21475500 1.60093400 -2.48980100

H -0.58840200 -1.66738300 -3.06031600

H -1.10975100 -0.73171600 -5.29723200

H 0.06353900 3.18927400 -3.93616500

H 0.58718500 2.24554600 -1.69136900

C -0.88525000 1.84801700 -6.14204500

H -1.94879300 1.68744300 -6.38745900

H -0.68031400 2.92651800 -6.20910900

H -0.29761500 1.33910600 -6.92460500

C 0.37595800 -0.27068700 -0.87847300

O 0.75456100 0.40513700 0.04549700

O 0.20052200 -1.63222700 -0.78745600

6

E (SMD/B3LYP-D3/def2-SVP) = -504.246122

H (SMD/B3LYP-D3/def2-SVP) = -503.979907

G (SMD/B3LYP-D3/def2-SVP) = -504.037917

E (SMD/B3LYP-D3/def2-TZVP//SMD/B3LYP-D3/def2-SVP) = -504.805988

C -4.02382900 0.64272100 -2.80194300

C -5.09815700 1.27969500 -3.70421700

H -5.96270500 1.59668000 -3.10192400

H -4.68602700 2.16611200 -4.20789000

H -5.43662800 0.56528500 -4.47143400

C -4.62168900 -0.59641000 -2.06562000

H -3.87673200 -1.04031500 -1.38898900

H -5.50843400 -0.30865600 -1.48278800

H -4.90993200 -1.34386900 -2.82102200

C -2.78432500 0.22744000 -3.61233400

H -2.35068800 1.10863600 -4.10824600

H -2.02157900 -0.20962800 -2.94955800

H -3.04230000 -0.51150100 -4.38773800

C -2.09093600 3.98534300 -4.63798600

C -2.15221600 3.87222600 -3.24023700

C -3.30892700 4.33764700 -2.58817900

C -4.36152600 4.90701800 -3.30849700

C -4.28463200 5.01687700 -4.70159300

C -3.14561600 4.55081500 -5.36438000

H -1.20363700 3.62421400 -5.16642900

H -3.38429000 4.24051000 -1.50259000

H -5.25173500 5.25907500 -2.78010600

H -5.10972100 5.45869100 -5.26646500

H -3.07492400 4.62704600 -6.45298400

C -1.02677900 3.26198400 -2.44374800

H -1.40894200 2.44388500 -1.81390200

H -0.23489000 2.86057900 -3.09421700

H -0.56547700 4.00783400 -1.77367500

O -3.70220300 1.49103100 -1.76910800

TS6-7

E (SMD/B3LYP-D3/def2-SVP) = -504.234911

H (SMD/B3LYP-D3/def2-SVP) = -503.97452

G (SMD/B3LYP-D3/def2-SVP) = -504.030336

E (SMD/B3LYP-D3/def2-TZVP//SMD/B3LYP-D3/def2-SVP) = -504.794968

C -3.73787900 0.38739600 -2.67066100

C -4.90011600 1.26471100 -3.16469800

H -5.54728400 1.54357400 -2.31841900

H -4.52262700 2.19029100 -3.62343500

H -5.51081000 0.73782700 -3.91585800

C -4.28628400 -0.90074100 -2.01790100

H -3.45955500 -1.52835600 -1.65200000

H -4.93637600 -0.65080600 -1.16564600

H -4.87218100 -1.47530200 -2.75328700

C -2.79150600 0.02507600 -3.82733900

H -2.40787400 0.93715900 -4.31008000

H -1.93393900 -0.55272700 -3.44882100

H -3.30782500 -0.57119900 -4.59673100

C -2.28990400 3.87973900 -4.68097000

C -2.45993500 3.95431800 -3.28258700

C -3.42965400 4.84654200 -2.77728400

C -4.19745500 5.63228300 -3.63586000

C -4.01649900 5.54471400 -5.02227500

C -3.05694200 4.66707900 -5.54071500

H -1.54292400 3.19513300 -5.09236100

H -3.58231500 4.91042700 -1.69623200

H -4.94387300 6.31705300 -3.22452900

H -4.61974000 6.15973200 -5.69505900

H -2.90666500 4.59684400 -6.62131300

C -1.70434800 3.07394400 -2.37489800

H -2.39245700 2.09246000 -2.05274800

H -0.79913000 2.62634700 -2.80701600

H -1.51220100 3.49646500 -1.37772900

O -3.03740900 1.01224900 -1.62565700

7

E (SMD/B3LYP-D3/def2-SVP) = -270.737693

H (SMD/B3LYP-D3/def2-SVP) = -270.616468

G (SMD/B3LYP-D3/def2-SVP) = -270.65268

E (SMD/B3LYP-D3/def2-TZVP//SMD/B3LYP-D3/def2-SVP) = -271.031577

C -2.40546800 -0.49402800 -0.00181200

C -1.00120500 -0.49919300 0.04596300

C -0.30183500 0.71866700 0.00122000

C -0.98793700 1.92255200 -0.08948800

C -2.41696700 1.95717200 -0.13943300

C -3.10328300 0.70312800 -0.09241900

H -2.95369800 -1.43956300 0.03266400

H -0.45662000 -1.44396900 0.11741600

H 0.79108100 0.71912300 0.03800800

H -0.43637400 2.86632700 -0.12391100

H -4.19635700 0.69885300 -0.12905300

C -3.11896300 3.17532200 -0.23178000

H -4.21123200 3.19432800 -0.26989600

H -2.58782100 4.12997500 -0.26799700

tBuO-OH

E (SMD/B3LYP-D3/def2-SVP) = -233.519719

H (SMD/B3LYP-D3/def2-SVP) = -233.377105

G (SMD/B3LYP-D3/def2-SVP) = -233.413449

E (SMD/B3LYP-D3/def2-TZVP//SMD/B3LYP-D3/def2-SVP) = -233.790037

C -2.61138700 -0.37530900 0.05116500

C -2.05543500 -1.79879400 -0.03442100

H -2.41337400 -2.30292700 -0.94534900

H -0.95471700 -1.78673500 -0.05342300

H -2.38306300 -2.38238300 0.83938300

C -4.14309800 -0.37893000 0.02028000

H -4.53469200 0.64807000 0.01969500

H -4.50556100 -0.88614100 -0.88767000

H -4.54628100 -0.90512100 0.89975200

C -2.07366500 0.35313400 1.28701900

H -0.97238600 0.36250600 1.27578500

H -2.42595800 1.39403400 1.30897200

H -2.40993500 -0.14821900 2.20814600

O -2.09022400 0.22384600 -1.14657000

O -2.54964200 1.60618200 -1.19762600

H -2.12763700 1.86453900 -2.03202000

4

E (SMD/B3LYP-D3/def2-SVP) = -2013.926628

H (SMD/B3LYP-D3/def2-SVP) = -2013.909207

G (SMD/B3LYP-D3/def2-SVP) = -2013.943855

E (SMD/B3LYP-D3/def2-TZVP//SMD/B3LYP-D3/def2-SVP) = -2014.280478

Cu 0.65399600 -0.24819900 0.05094200

O -0.60140100 1.03711700 0.12989500

H -1.50418600 0.79077600 -0.13306200

I 2.44765500 -1.89208500 -0.04953000

5

E (SMD/B3LYP-D3/def2-SVP) = -2933.702985

H (SMD/B3LYP-D3/def2-SVP) = -2933.374419

G (SMD/B3LYP-D3/def2-SVP) = -2933.461625

E (SMD/B3LYP-D3/def2-TZVP//SMD/B3LYP-D3/def2-SVP) = -2935.061559

Cu -0.00634000 -1.43882500 0.31151000

O -0.03765600 0.50256100 0.67619300

H 0.58414300 1.01016600 0.12986700

I 2.40844500 -1.32666100 -0.67736300

C -4.33077600 -0.85837200 0.41093100

C -5.27821200 0.10832100 0.03194000

C -6.63875000 -0.17760600 0.09350500

C -7.09931800 -1.42664700 0.55069100

C -6.14657100 -2.37964900 0.94306900

C -4.77996000 -2.10395000 0.87321300

H -4.92634700 1.08245200 -0.31271400

H -7.36429000 0.58105400 -0.21430700

H -6.47964400 -3.35322400 1.31289100

H -4.06099600 -2.85779700 1.19657600

C -8.57665300 -1.71487700 0.61476300

H -9.09339500 -0.98784600 1.26387500

H -9.03816200 -1.63436400 -0.38406700

H -8.78219400 -2.72329900 1.00325700

C -2.87613600 -0.51946800 0.34536600

O -2.03727100 -1.51107800 0.41305400

O -2.52138300 0.66523800 0.23453500

C -0.45124300 -5.72062800 0.26566100

C -1.42683500 -6.72281500 0.12143400

C -1.05481600 -8.06397000 0.14856300

C 0.28996100 -8.44188400 0.31783500

C 1.25637600 -7.42906200 0.45361800

C 0.89618500 -6.08458400 0.43078100

H -2.47291300 -6.44241600 -0.01178100

H -1.81956100 -8.83726500 0.03556100

H 2.30772800 -7.70217600 0.57698300

H 1.65016900 -5.30172900 0.53221000

C 0.67715400 -9.89524400 0.38210700

H 0.56290200 -10.27862800 1.41181300

H 0.03852200 -10.51471400 -0.26648600

H 1.72656000 -10.05126100 0.08981900

C -0.81650900 -4.29122700 0.24782800

O 0.04426300 -3.41745200 0.47263300

O -2.06538800 -4.01224100 -0.01208900

H -2.21398400 -3.00909000 0.08700500

H -0.99989800 0.77125300 0.45575900

H2O

E (SMD/B3LYP-D3/def2-SVP) = -76.362192

H (SMD/B3LYP-D3/def2-SVP) = -76.337291

G (SMD/B3LYP-D3/def2-SVP) = -76.359392

E (SMD/B3LYP-D3/def2-TZVP//SMD/B3LYP-D3/def2-SVP) = -76.467137

O -2.16553600 2.01122400 0.00000000

H -1.19933900 2.06767400 0.00000000

H -2.43484700 2.94084600 0.00000000

tBuOH

E (SMD/B3LYP-D3/def2-SVP) = -308.592184

H (SMD/B3LYP-D3/def2-SVP) = -308.445872

G (SMD/B3LYP-D3/def2-SVP) = -308.484581

E (SMD/B3LYP-D3/def2-TZVP//SMD/B3LYP-D3/def2-SVP) = -308.953682

C -3.72906500 0.38495500 -2.67767800

C -4.88136400 1.27377100 -3.16434300

H -5.52739300 1.55636800 -2.31838700

H -4.49259700 2.20051700 -3.62084100

H -5.49820700 0.76187800 -3.92060400

C -4.26580200 -0.88003400 -2.00837300

H -3.43289600 -1.49323000 -1.63013200

H -4.90818700 -0.61548800 -1.15387600

H -4.85395100 -1.48462600 -2.71600800

C -2.78949400 0.02965200 -3.83763400

H -2.37977700 0.94372400 -4.30129600

H -1.94430200 -0.57413500 -3.47165900

H -3.31154300 -0.53883200 -4.62424400

H -2.63565000 1.87904900 -2.04283100

O -2.99389200 1.06804300 -1.65537400

tBuO-O.

E (SMD/B3LYP-D3/def2-SVP) = -307.960023

H (SMD/B3LYP-D3/def2-SVP) = -307.824967

G (SMD/B3LYP-D3/def2-SVP) = -307.864382

E (SMD/B3LYP-D3/def2-TZVP//SMD/B3LYP-D3/def2-SVP) = -308.319264

C -2.61996400 -0.40617200 0.08306100

C -2.05432100 -1.81536600 -0.02669600

H -2.41192400 -2.31109700 -0.94223400

H -0.95386400 -1.79545200 -0.04847700

H -2.37320700 -2.41475400 0.83956900

C -4.14261400 -0.37865200 0.01854300

H -4.50482200 0.65903600 -0.00827200

H -4.50581300 -0.90150000 -0.87985400

H -4.56466800 -0.87590800 0.90524000

C -2.07246000 0.35341200 1.28586100

H -0.97134200 0.34897000 1.28339000

H -2.41983800 1.39635800 1.26948400

H -2.42010500 -0.11878200 2.21740900

O -2.09696600 0.25512900 -1.15375400

O -2.46187100 1.50111500 -1.27697100

TST-P

E (SMD/B3LYP-D3/def2-SVP) = -579.325329

H (SMD/B3LYP-D3/def2-SVP) = -579.060044

G (SMD/B3LYP-D3/def2-SVP) = -579.119037

E (SMD/B3LYP-D3/def2-TZVP//SMD/B3LYP-D3/def2-SVP) = -579.976081

C -2.74918300 -0.30713200 -0.24964700

C -2.23672200 -1.70214500 -0.60080900

H -3.04826700 -2.31969000 -1.01544200

H -1.42989000 -1.64443600 -1.34780000

H -1.84475100 -2.20026700 0.29908800

C -3.90479200 -0.36347200 0.74989800

H -4.31614000 0.64303300 0.91279200

H -4.71121800 -1.00954800 0.36938800

H -3.56174400 -0.76417900 1.71659900

C -1.62060600 0.59830800 0.24480100

H -0.81306300 0.65852400 -0.50128500

H -1.99564000 1.61282000 0.43984600

H -1.19754200 0.20193700 1.18083600

C -2.91652300 3.67952300 -4.52345800

C -2.54239600 3.96271200 -3.18766300

C -2.84704900 5.24451900 -2.67054300

C -3.48320300 6.20231100 -3.45756400

C -3.83963200 5.90529300 -4.77974900

C -3.55340200 4.63872200 -5.30737800

H -2.70043500 2.69055400 -4.93726100

H -2.57317700 5.48054600 -1.63830400

H -3.70431500 7.18855600 -3.04071500

H -4.33911300 6.65714100 -5.39618100

H -3.83082000 4.40128600 -6.33788600

C -1.91857700 2.94431700 -2.35424600

H -2.91427900 2.19190200 -1.85301400

H -1.39128900 2.13499500 -2.87733900

H -1.40174900 3.29600800 -1.45276900

O -3.24915500 0.17596600 -1.53209500

O -3.76603600 1.45687600 -1.43038300

TS1

E (SMD/B3LYP-D3/def2-SVP) = -1071.524834

H (SMD/B3LYP-D3/def2-SVP) = -1071.161865

G (SMD/B3LYP-D3/def2-SVP) = -1071.244284

E (SMD/B3LYP-D3/def2-TZVP//SMD/B3LYP-D3/def2-SVP) = -1072.688947

C -1.02975400 -4.74866100 0.03685200

C 1.84626500 -4.52026200 -1.03782000

C 0.82932800 -5.36483200 -1.63229300

C 1.18957000 -6.18095400 -2.72311100

C 0.30076000 -7.09839300 -3.27654500

C -0.98273900 -7.24458400 -2.73649000

C -1.35386800 -6.46646800 -1.64657200

C -0.48637600 -5.49428800 -1.09409600

H 2.20163500 -6.09531400 -3.11981700

H 0.61683600 -7.71629800 -4.12070300

H -1.68292200 -7.97099200 -3.15491600

H -2.34916300 -6.58900300 -1.21151400

C 2.50352900 -4.22809300 -0.01823800

C 3.36922800 -3.70692700 0.96444900

C 3.02372800 -2.53588900 1.68892400

C 4.61210900 -4.33580900 1.24023500

C 3.89335200 -2.02015800 2.64656300

H 2.07867700 -2.03368800 1.47434100

C 5.46880100 -3.80878800 2.20157100

H 4.89011600 -5.23037300 0.67938700

C 5.11642700 -2.65107200 2.90984300

H 3.61681000 -1.11426700 3.19219400

H 6.42394600 -4.30168300 2.40102900

H 5.79404400 -2.24122800 3.66296600

C -0.89706000 -3.43900400 0.40625300

H -1.71290300 -5.32563300 0.66889800

C -0.19661800 -2.42825600 -0.32944000

C -1.55930800 -2.97045500 1.59336500

N 0.32726400 -1.55911100 -0.89246200

N -2.08750900 -2.59039700 2.55458500

C 6.67795800 -4.17831100 -3.24480000

C 6.27736500 -3.27328900 -2.25229200

C 4.93231200 -2.94572600 -2.09874300

C 3.94002300 -3.52258400 -2.93298100

C 4.36783500 -4.42962200 -3.93682800

C 5.71469700 -4.74919700 -4.08931400

H 7.73430000 -4.43220500 -3.36484100

H 7.02241700 -2.82044600 -1.59251000

H 4.62716100 -2.24088700 -1.32195100

H 3.62463500 -4.87139600 -4.60681500

H 6.02190500 -5.44598200 -4.87411000

C 2.54795700 -3.22563700 -2.72385900

H 2.28050900 -2.31705700 -2.18393900

H 1.82439400 -3.55255000 -3.47431800

TS2

E (SMD/B3LYP-D3/def2-SVP) = -1071.528855

H (SMD/B3LYP-D3/def2-SVP) = -1071.165944

G (SMD/B3LYP-D3/def2-SVP) = -1071.246674

E (SMD/B3LYP-D3/def2-TZVP//SMD/B3LYP-D3/def2-SVP) = -1072.692703

C -1.25678500 -4.71748800 -0.35747500

C 1.84227300 -4.62455900 -0.51293200

C 1.02626400 -5.41479100 -1.33381300

C 1.68608000 -6.27656400 -2.25856100

C 0.98503800 -7.16853800 -3.05274700

C -0.41355100 -7.26823700 -2.93634600

C -1.08105200 -6.45820300 -2.03000900

C -0.40963200 -5.49015200 -1.24033300

H 2.77457100 -6.22121100 -2.32108000

H 1.52450400 -7.80710100 -3.75679300

H -0.97013700 -7.98401800 -3.54518800

H -2.16609000 -6.54621100 -1.93095500

C 2.70331300 -3.89032700 0.00533700

C 3.55581700 -3.47069100 1.09469800

C 3.22559300 -2.34252900 1.87235300

C 4.74950100 -4.16759100 1.36343900

C 4.06537200 -1.93815100 2.91009200

H 2.31320800 -1.78663900 1.64668400

C 5.58431200 -3.75499200 2.40442500

H 5.01583900 -5.03008500 0.74961400

C 5.24582400 -2.64213500 3.18015900

H 3.79857000 -1.06508400 3.51119600

H 6.50738000 -4.30452800 2.60612400

H 5.90204300 -2.31970300 3.99268500

C -1.15970300 -3.44899800 0.16598800

H -2.18228500 -5.23129100 -0.07740700

C -0.20269900 -2.44317000 -0.17851700

C -2.18024100 -3.00161800 1.07353200

N 0.50215100 -1.55894400 -0.44390600

N -3.00265800 -2.64246100 1.81083700

C 7.27494400 -3.81962000 -2.52953500

C 7.07720500 -2.87371600 -1.51413800

C 5.79604600 -2.41442800 -1.21971200

C 4.66854000 -2.88716900 -1.94206000

C 4.89123000 -3.84526500 -2.96761400

C 6.17380300 -4.30126300 -3.25465800

H 8.28177000 -4.17867400 -2.75764400

H 7.93170000 -2.49509800 -0.94695900

H 5.64568400 -1.68220200 -0.42292300

H 4.03507200 -4.22071400 -3.53507100

H 6.32465500 -5.03544300 -4.05081500

C 3.34340800 -2.45960400 -1.60692700

H 3.19984000 -1.58830000 -0.96647600

H 2.52641200 -2.67175700 -2.29809800

TS3

E (SMD/B3LYP-D3/def2-SVP) = -1071.54541

H (SMD/B3LYP-D3/def2-SVP) = -1071.181661

G (SMD/B3LYP-D3/def2-SVP) = -1071.262763

E (SMD/B3LYP-D3/def2-TZVP//SMD/B3LYP-D3/def2-SVP) = -1072.710015

C -0.14008500 -2.91290100 -2.26352500

C 1.17467000 -5.04014100 -0.85063600

C 0.74039200 -5.24053300 -2.19273800

C 0.94591400 -6.49721000 -2.80067300

C 0.51051100 -6.74270300 -4.09912800

C -0.14394900 -5.73348600 -4.81688900

C -0.35150000 -4.48459000 -4.23577500

C 0.08755700 -4.20181500 -2.92779900

H 1.44989200 -7.27934100 -2.22971500

H 0.67570200 -7.72304400 -4.55264500

H -0.49683200 -5.92045500 -5.83376600

H -0.87616400 -3.72168000 -4.80972400

C 1.51154800 -4.87434100 0.30971400

C 1.85140000 -4.66243100 1.67809900

C 1.75060500 -3.37230800 2.24446900

C 2.26059700 -5.73873700 2.49516200

C 2.04814500 -3.17066300 3.59174700

H 1.43499200 -2.53481600 1.61853100

C 2.55590700 -5.52638400 3.84135000

H 2.33712300 -6.73868400 2.06288600

C 2.45004500 -4.24480000 4.39443000

H 1.96627800 -2.16790500 4.01873000

H 2.87039700 -6.36715700 4.46498900

H 2.68195200 -4.08284300 5.45015600

C -0.23578100 -1.63864300 -2.85952100

H 0.19318200 -2.87847000 -1.22592600

C -0.43190000 -1.37802800 -4.24351500

C -0.22572200 -0.49979700 -2.00105200

N -0.59667300 -1.15244700 -5.37412800

N -0.22861400 0.40332100 -1.26650300

C -1.80386100 -6.21535300 1.50731400

C -2.05722200 -6.59229100 0.18107000

C -2.22128500 -5.62274200 -0.80296000

C -2.14121800 -4.24292300 -0.47896500

C -1.88704700 -3.88229900 0.87155500

C -1.72288800 -4.85656900 1.84826800

H -1.66471600 -6.97919300 2.27636400

H -2.11845200 -7.65090700 -0.08343900

H -2.40550000 -5.91579200 -1.83934100

H -1.81023900 -2.82342300 1.13351500

H -1.51627000 -4.56445800 2.88048000

C -2.24268000 -3.23949000 -1.49327600

H -2.63088300 -3.50917800 -2.47746100

H -2.38391400 -2.20088700 -1.18677100

TS4

E (SMD/B3LYP-D3/def2-SVP) = -1071.525149

H (SMD/B3LYP-D3/def2-SVP) = -1071.162512

G (SMD/B3LYP-D3/def2-SVP) = -1071.243558

E (SMD/B3LYP-D3/def2-TZVP//SMD/B3LYP-D3/def2-SVP) = -1072.689108

C -0.27143300 -3.34844600 -1.67166200

C 1.77686600 -4.81236700 -0.07438400

C 0.89120600 -5.49204500 -0.95602500

C 1.00586400 -6.89043200 -1.10838200

C 0.27415900 -7.57212500 -2.07665400

C -0.56874800 -6.86235200 -2.94483600

C -0.70204000 -5.48577900 -2.80841800

C -0.03150900 -4.77229800 -1.78691600

H 1.70518600 -7.42937600 -0.46591400

H 0.37857000 -8.65550600 -2.17372100

H -1.12662100 -7.38805900 -3.72345900

H -1.37581600 -4.93843500 -3.47007100

C 2.56182000 -4.20990600 0.63957400

C 3.42981700 -3.45753600 1.48387100

C 3.35885200 -2.04589100 1.49625500

C 4.36465000 -4.10333400 2.32272000

C 4.19992700 -1.30809500 2.32802600

H 2.63925400 -1.53630000 0.85173000

C 5.20152400 -3.35515500 3.14978100

H 4.42230500 -5.19394200 2.31843900

C 5.12280900 -1.95761400 3.15615100

H 4.13355600 -0.21714900 2.33036100

H 5.92019000 -3.86574500 3.79603500

H 5.77988400 -1.37490000 3.80687800

C -0.51391700 -2.59504500 -0.48715400

H -0.42215800 -2.79423600 -2.60334800

C -0.13994800 -1.20027300 -0.49716900

C -0.50192400 -3.20422600 0.82198100

N 0.16125200 -0.07942700 -0.51775000

N -0.60392900 -3.67602800 1.87691000

C -4.48446200 -5.88701000 -1.54314200

C -4.25881700 -4.89925700 -2.50986500

C -3.68466500 -3.68371700 -2.14425100

C -3.33894100 -3.42294400 -0.79673600

C -3.58285100 -4.42920900 0.16947100

C -4.14489000 -5.64690600 -0.20456200

H -4.92868900 -6.84317900 -1.83123200

H -4.52753100 -5.08359600 -3.55324100

H -3.50325100 -2.91430900 -2.89976200

H -3.30933800 -4.24744500 1.21123000

H -4.32115900 -6.41738400 0.55011100

C -2.68288000 -2.19095400 -0.43939500

H -2.67230000 -1.38507000 -1.17778300

H -2.70206900 -1.86515800 0.60337800

TS3R1=R2=NO2

E (SMD/B3LYP-D3/def2-SVP) = -1295.890298

H (SMD/B3LYP-D3/def2-SVP) = -1295.516858

G (SMD/B3LYP-D3/def2-SVP) = -1295.602615

E (SMD/B3LYP-D3/def2-TZVP//SMD/B3LYP-D3/def2-SVP) = -1297.328458

C -0.29575400 -2.94954000 -2.11578500

C 1.13129000 -5.06257700 -0.86342100

C 0.63849700 -5.24782000 -2.18672600

C 0.85049800 -6.47505200 -2.84799700

C 0.35344600 -6.68889800 -4.13008800

C -0.37295600 -5.67950200 -4.77352100

C -0.59114600 -4.45863300 -4.13771400

C -0.08447500 -4.20640200 -2.84894800

H 1.40887800 -7.25908500 -2.33310100

H 0.52517200 -7.64635000 -4.62783400

H -0.77799200 -5.84478400 -5.77453800

H -1.18925500 -3.70758800 -4.65138900

C 1.50284200 -4.86664500 0.28145300

C 1.87136200 -4.58861800 1.63000000

C 1.71950000 -3.28081800 2.14342200

C 2.35688800 -5.60768900 2.47732200

C 2.04333000 -3.00766300 3.47183300

H 1.34454300 -2.48810800 1.49196700

C 2.67804800 -5.32284600 3.80416500

H 2.47268300 -6.62005700 2.08443200

C 2.52180900 -4.02530900 4.30580800

H 1.92191700 -1.99293600 3.85920000

H 3.05253600 -6.11878400 4.45296100

H 2.77402800 -3.80684400 5.34661100

H 0.10692400 -2.93456900 -1.10255800

C -1.82995600 -6.49345400 1.44946400

C -2.15813200 -6.74799100 0.10983500

C -2.34220200 -5.69291800 -0.77721200

C -2.20571600 -4.34922200 -0.33893300

C -1.87248200 -4.11288000 1.02203900

C -1.69117200 -5.17247900 1.90182900

H -1.67764300 -7.32519600 2.14188000

H -2.26294700 -7.77779500 -0.24051100

H -2.58530700 -5.88872400 -1.82430400

H -1.74556900 -3.08344700 1.36649100

H -1.42459700 -4.97649600 2.94296200

C -2.33119300 -3.25808700 -1.25336200

H -2.77975800 -3.41563100 -2.23581200

H -2.40000900 -2.24822200 -0.84402100

C -0.39280400 -1.66662000 -2.62708300

N -0.41158800 -0.52432800 -1.77649000

O -0.24069500 -0.69992400 -0.56685300

O -0.61270700 0.57052200 -2.30139400

N -0.79178600 -1.35170900 -4.00281200

O -1.96311100 -1.56331500 -4.28949200

O 0.05840300 -0.93354000 -4.76022600

TS3R1=R2=CN

E (SMD/B3LYP-D3/def2-SVP) = -1071.54541

H (SMD/B3LYP-D3/def2-SVP) = -1071.181661

G (SMD/B3LYP-D3/def2-SVP) = -1071.262763

E (SMD/B3LYP-D3/def2-TZVP//SMD/B3LYP-D3/def2-SVP) = -1072.710015

C -0.14008500 -2.91290100 -2.26352500

C 1.17467000 -5.04014100 -0.85063600

C 0.74039200 -5.24053300 -2.19273800

C 0.94591400 -6.49721000 -2.80067300

C 0.51051100 -6.74270300 -4.09912800

C -0.14394900 -5.73348600 -4.81688900

C -0.35150000 -4.48459000 -4.23577500

C 0.08755700 -4.20181500 -2.92779900

H 1.44989200 -7.27934100 -2.22971500

H 0.67570200 -7.72304400 -4.55264500

H -0.49683200 -5.92045500 -5.83376600

H -0.87616400 -3.72168000 -4.80972400

C 1.51154800 -4.87434100 0.30971400

C 1.85140000 -4.66243100 1.67809900

C 1.75060500 -3.37230800 2.24446900

C 2.26059700 -5.73873700 2.49516200

C 2.04814500 -3.17066300 3.59174700

H 1.43499200 -2.53481600 1.61853100

C 2.55590700 -5.52638400 3.84135000

H 2.33712300 -6.73868400 2.06288600

C 2.45004500 -4.24480000 4.39443000

H 1.96627800 -2.16790500 4.01873000

H 2.87039700 -6.36715700 4.46498900

H 2.68195200 -4.08284300 5.45015600

C -0.23578100 -1.63864300 -2.85952100

H 0.19318200 -2.87847000 -1.22592600

C -0.43190000 -1.37802800 -4.24351500

C -0.22572200 -0.49979700 -2.00105200

N -0.59667300 -1.15244700 -5.37412800

N -0.22861400 0.40332100 -1.26650300

C -1.80386100 -6.21535300 1.50731400

C -2.05722200 -6.59229100 0.18107000

C -2.22128500 -5.62274200 -0.80296000

C -2.14121800 -4.24292300 -0.47896500

C -1.88704700 -3.88229900 0.87155500

C -1.72288800 -4.85656900 1.84826800

H -1.66471600 -6.97919300 2.27636400

H -2.11845200 -7.65090700 -0.08343900

H -2.40550000 -5.91579200 -1.83934100

H -1.81023900 -2.82342300 1.13351500

H -1.51627000 -4.56445800 2.88048000

C -2.24268000 -3.23949000 -1.49327600

H -2.63088300 -3.50917800 -2.47746100

H -2.38391400 -2.20088700 -1.18677100

TS3R1=CN,R2=CO2Me

E (SMD/B3LYP-D3/def2-SVP) = -1246.392552

H (SMD/B3LYP-D3/def2-SVP) = -1245.952363

G (SMD/B3LYP-D3/def2-SVP) = -1246.042651

E (SMD/B3LYP-D3/def2-TZVP//SMD/B3LYP-D3/def2-SVP) = -1247.754565

C -0.46595700 -3.01987400 -2.10960300

C 1.11213700 -5.06402200 -0.93495000

C 0.62667600 -5.24867700 -2.26162400

C 0.91149600 -6.43685800 -2.96486900

C 0.42759100 -6.63338000 -4.25535500

C -0.35147600 -5.64296700 -4.86692200

C -0.63937600 -4.45965200 -4.18850300

C -0.16001200 -4.22901100 -2.88575000

H 1.51620100 -7.20428700 -2.47765100

H 0.65474900 -7.56110000 -4.78637600

H -0.73831500 -5.79329000 -5.87765200

H -1.25557600 -3.70646300 -4.67936500

C 1.46209700 -4.82756900 0.20898800

C 1.77713500 -4.46624100 1.55141300

C 1.41472400 -3.18393700 2.02477400

C 2.40999700 -5.37029800 2.43037700

C 1.67985400 -2.82597700 3.34606600

H 0.91967400 -2.48130600 1.34968500

C 2.66995300 -5.00047600 3.74999400

H 2.68761400 -6.36263000 2.06844300

C 2.30589700 -3.73047700 4.21252100

H 1.39434000 -1.83309100 3.70308600

H 3.15906100 -5.70854900 4.42397000

H 2.51047000 -3.44553900 5.24777900

H -0.10548200 -3.04074800 -1.08213300

C -1.66494200 -6.65938500 1.49787800

C -1.95598500 -6.97865500 0.16408600

C -2.24226200 -5.97030800 -0.75096600

C -2.24934600 -4.60976000 -0.34812700

C -1.95082200 -4.30703200 1.00668600

C -1.66630100 -5.31983300 1.91470100

H -1.43144300 -7.45310800 2.21197800

H -1.95086300 -8.02208100 -0.16119900

H -2.45551400 -6.21780600 -1.79371900

H -1.92750400 -3.26106300 1.32213800

H -1.42539300 -5.06970000 2.95062400

C -2.47824900 -3.55721100 -1.29234000

H -2.90439500 -3.79757400 -2.26849100

H -2.67868300 -2.55670900 -0.90654400

C -0.66527100 -1.70798600 -2.56484300

O -0.58420900 -0.85944700 -0.33292000

C -0.91477200 -1.34440400 -3.91299000

N -1.12752800 -1.06236600 -5.02349100

C -0.68133600 -0.64632400 -1.52827600

O -0.82146500 0.57711300 -2.05212300

C -0.84411000 1.69021500 -1.13781900

H 0.09324800 1.69324400 -0.55707100

H -1.67054200 1.54679300 -0.42188600

C -1.01113400 2.95197000 -1.95407900

H -1.95117400 2.92757700 -2.52719200

H -1.03225100 3.82835300 -1.28729700

H -0.17739600 3.07399600 -2.66302000

TS3R1=NO2,R2=H

E (SMD/B3LYP-D3/def2-SVP) = -1091.555779

H (SMD/B3LYP-D3/def2-SVP) = -1091.187738

G (SMD/B3LYP-D3/def2-SVP) = -1091.265536

E (SMD/B3LYP-D3/def2-TZVP//SMD/B3LYP-D3/def2-SVP) = -1092.753385

C -0.09804700 -2.93064400 -2.15711800

C 1.19238700 -5.13449500 -0.82617100

C 0.76526200 -5.28223000 -2.17764400

C 0.96044600 -6.50872700 -2.84873100

C 0.53904900 -6.67742600 -4.16424000

C -0.08863600 -5.62062000 -4.83730800

C -0.28710800 -4.40316900 -4.18898400

C 0.13398600 -4.20221700 -2.86235500

H 1.44735200 -7.32743100 -2.31518300

H 0.69560000 -7.63466600 -4.66749400

H -0.42762200 -5.74903400 -5.86813400

H -0.79499300 -3.59377200 -4.71856700

C 1.51904800 -4.97280400 0.33738100

C 1.83981000 -4.73944200 1.70692300

C 1.72708500 -3.43778200 2.24484000

C 2.23727200 -5.79779000 2.55200800

C 2.00110400 -3.20880800 3.59276400

H 1.41844500 -2.61445700 1.59693100

C 2.50897600 -5.55778200 3.89873700

H 2.32231800 -6.80622500 2.14148700

C 2.39098200 -4.26566000 4.42404600

H 1.90906600 -2.19786700 3.99795600

H 2.81397700 -6.38521600 4.54462400

H 2.60371700 -4.08208900 5.48032700

H 0.21495300 -2.88957600 -1.11351900

C -1.84558100 -6.21443600 1.54243300

C -2.12490700 -6.56799800 0.21536900

C -2.24763300 -5.58303900 -0.76042600

C -2.09880600 -4.21302900 -0.42681600

C -1.81620400 -3.87529500 0.92219300

C -1.69482500 -4.86423600 1.89144100

H -1.73900100 -6.99009100 2.30496800

H -2.23926300 -7.62033400 -0.05701600

H -2.45218000 -5.85936100 -1.79778000

H -1.67938400 -2.82380100 1.18762100

H -1.46486200 -4.58966600 2.92358400

C -2.15804600 -3.19166000 -1.43384400

H -2.58103000 -3.43842600 -2.40991500

H -2.25688400 -2.15552700 -1.10535300

C -0.14614700 -1.70567900 -2.80433400

H -0.18589200 -1.54013500 -3.87890800

N -0.21702700 -0.50753200 -2.04838600

O -0.19669700 0.55629100 -2.67574000

O -0.30197600 -0.57289800 -0.81356400

TS3R1=CN,R2=H

E (SMD/B3LYP-D3/def2-SVP) = -979.371838

H (SMD/B3LYP-D3/def2-SVP) = -979.009141

G (SMD/B3LYP-D3/def2-SVP) = -979.085802

E (SMD/B3LYP-D3/def2-TZVP//SMD/B3LYP-D3/def2-SVP) = -980.431112

C -0.27167200 -2.91239900 -2.28795000

C 1.12224000 -5.01514800 -0.86046300

C 0.64792500 -5.24049400 -2.18652000

C 0.85275300 -6.50875100 -2.77280100

C 0.37562400 -6.79308500 -4.04808900

C -0.32139500 -5.81033600 -4.76149700

C -0.52730300 -4.55138900 -4.19947400

C -0.04530900 -4.22921300 -2.91734300

H 1.38821600 -7.26940100 -2.20114900

H 0.54037400 -7.78110900 -4.48491100

H -0.70908700 -6.02491800 -5.76033500

H -1.08300100 -3.80818500 -4.77082100

C 1.49572800 -4.85506200 0.28934100

C 1.87757500 -4.66297700 1.64999800

C 1.74818500 -3.39432800 2.25724000

C 2.36069900 -5.74192500 2.42229300

C 2.08965000 -3.21565400 3.59746500

H 1.37425800 -2.55532500 1.66668400

C 2.69986900 -5.55332100 3.76175300

H 2.45993800 -6.72636400 1.96006000

C 2.56525900 -4.29253700 4.35472500

H 1.98400000 -2.22879900 4.05552500

H 3.07123200 -6.39714600 4.34902400

H 2.83135600 -4.14916100 5.40507200

H 0.10401200 -2.83773600 -1.26633900

C -1.82669800 -6.21200100 1.53629200

C -2.09813800 -6.58568900 0.21361500

C -2.28000900 -5.61455700 -0.76741900

C -2.19936600 -4.23576700 -0.44661600

C -1.92905300 -3.87869300 0.89968000

C -1.74688700 -4.85318800 1.87478000

H -1.67367700 -6.97607100 2.30251700

H -2.15948600 -7.64388400 -0.05341600

H -2.47699500 -5.90893600 -1.80108300

H -1.85443300 -2.82010600 1.16451400

H -1.52792900 -4.55940500 2.90426000

C -2.31247600 -3.22949300 -1.46668600

H -2.74377000 -3.50279600 -2.43205200

H -2.47519500 -2.19566600 -1.15448200

C -0.38197800 -1.67624300 -2.93476800

H -0.34694200 -0.77471900 -2.31506000

C -0.62843100 -1.44208800 -4.30265000

N -0.83445600 -1.21335100 -5.42952000

TS3R1=R2=H

E (SMD/B3LYP-D3/def2-SVP) = -887.18627

H (SMD/B3LYP-D3/def2-SVP) = -886.825112

G (SMD/B3LYP-D3/def2-SVP) = -886.897329

E (SMD/B3LYP-D3/def2-TZVP//SMD/B3LYP-D3/def2-SVP) = -888.140085

C -0.19776700 -2.86284300 -2.24789600

C 1.19156100 -5.00018600 -0.80451200

C 0.76537500 -5.19826400 -2.15171500

C 1.00887300 -6.44797000 -2.76606900

C 0.58698000 -6.70138900 -4.06668300

C -0.09166900 -5.70417300 -4.77951100

C -0.33427200 -4.46635200 -4.18655600

C 0.08757700 -4.17719600 -2.87676600

H 1.53121200 -7.21859100 -2.19527300

H 0.77993400 -7.67475400 -4.52447700

H -0.43624500 -5.89475900 -5.79920300

H -0.87987500 -3.70559200 -4.74967600

C 1.53107100 -4.86155500 0.35843700

C 1.87708500 -4.69003100 1.73140700

C 1.68944200 -3.44211200 2.36605000

C 2.38630100 -5.76805200 2.48811300

C 2.00023900 -3.28303900 3.71622100

H 1.29364400 -2.60450300 1.78814400

C 2.69425300 -5.59931500 3.83783100

H 2.53048500 -6.73691100 2.00513800

C 2.50237600 -4.35902200 4.45755300

H 1.84916100 -2.31193200 4.19496200

H 3.08613500 -6.44291700 4.41208700

H 2.74416100 -4.23097600 5.51578200

H 0.14711400 -2.77134900 -1.21480100

C -1.81971800 -6.15169700 1.52589700

C -2.04426400 -6.54323400 0.20016300

C -2.19895200 -5.58595600 -0.80056300

C -2.13652700 -4.20327100 -0.50033400

C -1.91659500 -3.82824200 0.84841900

C -1.76156900 -4.78774500 1.84465900

H -1.68683700 -6.90373900 2.30771600

H -2.08979300 -7.60536500 -0.05541700

H -2.35780100 -5.89819500 -1.83577300

H -1.85776800 -2.76550400 1.10140800

H -1.58016300 -4.47605400 2.87644800

C -2.20336400 -3.20600600 -1.54462000

H -2.62802900 -3.49912400 -2.50791900

H -2.42071400 -2.17794400 -1.24355300

C -0.25968500 -1.68215000 -2.98341800

H -0.35958100 -1.67088800 -4.07162400

H -0.30263500 -0.71729800 -2.47111300

8

E (SMD/B3LYP-D3/def2-SVP) = -1071.576558

H (SMD/B3LYP-D3/def2-SVP) = -1071.210041

G (SMD/B3LYP-D3/def2-SVP) = -1071.291449

E (SMD/B3LYP-D3/def2-TZVP//SMD/B3LYP-D3/def2-SVP) = -1072.737597

C -0.23887800 -4.54558800 1.11650600

C 0.25324600 -2.96455500 -1.31825000

C 0.51087000 -4.36765500 -1.32803200

C 0.95782300 -4.98574400 -2.51694400

C 1.18744000 -6.35805900 -2.56675400

C 0.97217500 -7.14385000 -1.42926600

C 0.53083400 -6.54578600 -0.24766100

C 0.29568900 -5.16629900 -0.17377800

H 1.11740500 -4.36716800 -3.40232800

H 1.53452200 -6.81718700 -3.49562200

H 1.14820000 -8.22171400 -1.46077400

H 0.36020700 -7.16903800 0.63380400

C 0.00457500 -1.77053000 -1.31438700

C -0.31917400 -0.38110600 -1.29606400

C -0.64671300 0.26058400 -0.08097900

C -0.33005800 0.37221600 -2.49038900

C -0.97748900 1.61501700 -0.06629400

H -0.63648300 -0.31437700 0.84735000

C -0.66171000 1.72645900 -2.46503200

H -0.07855900 -0.11806200 -3.43328500

C -0.98669400 2.35231100 -1.25602600

H -1.22827900 2.10022100 0.88049300

H -0.66698600 2.29899800 -3.39608900

H -1.24556300 3.41405600 -1.24068700

C 0.67626600 -4.89659800 2.27823100

H -0.18389500 -3.45292300 1.00286000

C 0.38852100 -5.96004200 3.15418700

C 1.87863300 -4.18412900 2.46222100

N 0.11393600 -6.84846500 3.86136700

N 2.86107000 -3.56871300 2.59760900

C -4.21671200 -3.26783100 -1.73265400

C -3.91340900 -2.51029100 -0.59664600

C -3.12955800 -3.05981900 0.42062400

C -2.63601600 -4.37029800 0.32216400

C -2.95153900 -5.12254300 -0.81968200

C -3.73632100 -4.57655900 -1.83961900

H -4.82678300 -2.83895100 -2.53191000

H -4.28269000 -1.48559500 -0.50481200

H -2.89011100 -2.45952700 1.30346500

H -2.56897100 -6.14213000 -0.91412700

H -3.97045200 -5.17578900 -2.72352400

C -1.71903000 -4.92158100 1.38945700

H -2.00231300 -4.51936300 2.37500300

H -1.80981200 -6.01740300 1.44941200

TS8-9

E (SMD/B3LYP-D3/def2-SVP) = -1071.561458

H (SMD/B3LYP-D3/def2-SVP) = -1071.196509

G (SMD/B3LYP-D3/def2-SVP) = -1071.275582

E (SMD/B3LYP-D3/def2-TZVP//SMD/B3LYP-D3/def2-SVP) = -1072.722034

C -1.40785200 -3.90887600 0.08607800

C 0.44000700 -2.31571400 -1.16279700

C 0.21386700 -3.60842500 -1.76762300

C 0.88125900 -4.00325800 -2.94021700

C 0.64065800 -5.26627100 -3.48051200

C -0.26237900 -6.13216300 -2.85486100

C -0.93248200 -5.73993700 -1.69069000

C -0.70286400 -4.47773500 -1.13761900

H 1.59009700 -3.31919000 -3.41127400

H 1.16426200 -5.57817200 -4.38749100

H -0.44774600 -7.12450600 -3.27347300

H -1.63848700 -6.42418100 -1.22222600

C 0.87521200 -1.14522800 -1.15959100

C 1.27382700 0.18377900 -0.91944000

C 0.35571100 1.25299700 -1.09054600

C 2.59520900 0.47177100 -0.48827400

C 0.75351600 2.56148400 -0.83659700

H -0.66500000 1.03296500 -1.40860900

C 2.97756500 1.78623900 -0.24083400

H 3.30101300 -0.34965600 -0.34893100

C 2.06192100 2.83348200 -0.41398400

H 0.03886800 3.37832300 -0.96332800

H 3.99569900 1.99992700 0.09368300

H 2.36777300 3.86378700 -0.21557900

C -0.48531000 -2.85546900 0.71526600

H -2.28500000 -3.34927700 -0.27965600

C 0.65999600 -3.31582300 1.43787100

C -1.07851400 -1.65011100 1.19491300

N 1.60087100 -3.71300100 1.99526400

N -1.56515500 -0.64580900 1.52429600

C -5.45304900 -7.19529500 0.14206900

C -5.47174300 -5.79706700 0.09774500

C -4.32364300 -5.06795200 0.41762300

C -3.13326900 -5.71917200 0.78163600

C -3.13061900 -7.12213300 0.83050800

C -4.27864800 -7.85570500 0.51379000

H -6.35087000 -7.76617400 -0.10859200

H -6.38734900 -5.27004000 -0.18371000

H -4.35861300 -3.97492100 0.39025800

H -2.21481100 -7.64694100 1.11742100

H -4.25327600 -8.94785700 0.55729400

C -1.89400400 -4.92681600 1.14926800

H -2.11491100 -4.35598500 2.06706400

H -1.06595900 -5.60773400 1.39888200

TS8-9'

E (SMD/B3LYP-D3/def2-SVP) = -1071.543622

H (SMD/B3LYP-D3/def2-SVP) = -1071.178656

G (SMD/B3LYP-D3/def2-SVP) = -1071.256199

E (SMD/B3LYP-D3/def2-TZVP//SMD/B3LYP-D3/def2-SVP) = -1072.703311

C -1.05551800 -3.88449900 -0.05569600

C 0.52385800 -2.53787300 -1.93765100

C 0.27188100 -3.91682300 -2.21929300

C 0.78380700 -4.55980600 -3.35831800

C 0.57800000 -5.92669700 -3.54266400

C -0.13020800 -6.65151800 -2.58244800

C -0.64408900 -6.01467300 -1.44608100

C -0.46969000 -4.64302000 -1.24333500

H 1.34981400 -3.97666100 -4.08764100

H 0.97698200 -6.42514300 -4.42922400

H -0.28938900 -7.72483700 -2.71177300

H -1.19695900 -6.61005900 -0.72250500

C 0.56057600 -1.68979600 -0.99586400

C 0.84578700 -0.35131700 -0.52294900

C -0.13856600 0.65356800 -0.57770600

C 2.10547300 -0.06193100 0.03823300

C 0.14250400 1.93292800 -0.09690200

H -1.11974800 0.42262000 -0.99739700

C 2.37569100 1.21859200 0.52040000

H 2.86163400 -0.84805600 0.09449500

C 1.39708000 2.21691300 0.45215800

H -0.62431100 2.70995200 -0.14456400

H 3.35467200 1.43861800 0.95320700

H 1.61203800 3.21873100 0.83271700

C -0.02649200 -2.92527400 0.59251400

H -1.82274400 -3.20551900 -0.46554100

C 1.22811300 -3.47598100 1.01981400

C -0.59085500 -1.96480000 1.49197200

N 2.25916700 -3.91964600 1.32306800

N -1.08435400 -1.15574900 2.16600300

C -5.50113400 -6.64244000 0.02438900

C -5.29990300 -5.28419100 -0.24139700

C -4.08103200 -4.67747000 0.07371200

C -3.03634700 -5.41509300 0.65426100

C -3.25523600 -6.77581400 0.92316500

C -4.47427000 -7.38609700 0.61270500

H -6.45416200 -7.11719300 -0.22280500

H -6.09860600 -4.69049000 -0.69402000

H -3.95038300 -3.61108700 -0.12943000

H -2.45774900 -7.36681600 1.38334300

H -4.62017700 -8.44748400 0.83058700

C -1.72242800 -4.75748000 1.03605500

H -1.91905700 -4.10642600 1.90383000

H -1.00360200 -5.51594500 1.38304900

9'

E (SMD/B3LYP-D3/def2-SVP) = -1071.56871

H (SMD/B3LYP-D3/def2-SVP) = -1071.201379

G (SMD/B3LYP-D3/def2-SVP) = -1071.27712

E (SMD/B3LYP-D3/def2-TZVP//SMD/B3LYP-D3/def2-SVP) = -1072.725112

C -1.17556500 -4.06851100 0.07479300

C 0.39628100 -2.40756700 -1.48145200

C 0.13894400 -3.69546000 -2.04935200

C 0.62407400 -4.09792400 -3.30481200

C 0.38967100 -5.39544900 -3.76060100

C -0.32310100 -6.29059600 -2.96002900

C -0.81321700 -5.89317600 -1.70842800

C -0.60101700 -4.59757000 -1.23426900

H 1.19222300 -3.38683400 -3.90834500

H 0.76829000 -5.70885900 -4.73640100

H -0.50509200 -7.31100600 -3.30594300

H -1.37641700 -6.61103600 -1.11532100

C 0.32058800 -1.96398200 -0.22797500

C 0.73910500 -0.61588300 0.21138200

C 0.61626000 0.46796400 -0.68267000

C 1.29666800 -0.37687200 1.48207900

C 1.03867600 1.74520400 -0.31769800

H 0.17023900 0.29924900 -1.66578200

C 1.71739500 0.90461500 1.84429700

H 1.42917800 -1.19284800 2.19485900

C 1.58976200 1.97028300 0.94914100

H 0.92884400 2.57288900 -1.02318200

H 2.15075200 1.06723300 2.83423400

H 1.91512800 2.97297600 1.23762400

C -0.18864500 -3.04876700 0.78804600

H -2.03003800 -3.42704700 -0.20341400

C 0.97011300 -3.76657200 1.36278800

C -0.94681700 -2.40398600 1.87774800

N 1.88069700 -4.34877300 1.77782500

N -1.58772300 -1.90758100 2.70378400

C -5.52354800 -6.94739900 0.21777900

C -5.38459800 -5.55573300 0.23696400

C -4.14069400 -4.97604300 0.50004800

C -3.00976800 -5.77312400 0.74176000

C -3.16573100 -7.16838200 0.73058700

C -4.40935000 -7.75249000 0.47045500

H -6.49629900 -7.40118000 0.01140300

H -6.25130800 -4.91588400 0.05096300

H -4.05546300 -3.88617300 0.52856700

H -2.30046300 -7.80758200 0.92949900

H -4.50621200 -8.84143400 0.46511900

C -1.66568300 -5.14661200 1.06605000

H -1.75610000 -4.68480600 2.06273500

H -0.89662700 -5.92761000 1.16374100

10

E (SMD/B3LYP-D3/def2-SVP) = -4005.323674

H (SMD/B3LYP-D3/def2-SVP) = -4004.626554

G (SMD/B3LYP-D3/def2-SVP) = -4004.763253

E (SMD/B3LYP-D3/def2-TZVP//SMD/B3LYP-D3/def2-SVP) = -4007.827849

Cu 0.91790200 -0.22106700 0.16935000

O 0.43989200 -2.04390900 0.96806400

H -0.50973000 -2.23685500 0.99968200

I 0.61959700 1.75185800 -1.39926300

C 3.54412100 -2.63098600 -2.30157500

C 3.76448300 -3.97204300 -2.65785600

C 4.76255200 -4.30248600 -3.57036600

C 5.56378900 -3.30810900 -4.16242300

C 5.32836500 -1.97001600 -3.80884000

C 4.33313700 -1.63212600 -2.89009600

H 3.14044500 -4.74437900 -2.20419800

H 4.92812400 -5.35126400 -3.83446600

H 5.93218700 -1.17932600 -4.26275700

H 4.15459400 -0.58626000 -2.63396900

C 6.64334100 -3.68631800 -5.14357600

H 7.42018100 -4.30098600 -4.65707500

H 6.23437000 -4.28766100 -5.97278800

H 7.13495300 -2.80155000 -5.57466900

C 2.46571600 -2.29079300 -1.32168800

O 2.45356700 -1.08363500 -0.86774500

O 1.63478900 -3.16010200 -0.98713700

C 4.48285400 1.72764000 2.14290700

C 5.82209500 2.02662300 1.84782400

C 6.55654100 2.85398500 2.69673900

C 5.97981500 3.40168200 3.85573100

C 4.63559900 3.09724600 4.13738300

C 3.89483700 2.27128100 3.29642400

H 6.28116800 1.60883800 0.95042100

H 7.59886700 3.08181900 2.45668100

H 4.16484600 3.51792300 5.03066200

H 2.85162200 2.03969500 3.51473600

C 6.78509000 4.26652000 4.79025100

H 7.20563900 3.66140000 5.61321900

H 7.62935200 4.74948200 4.27501100

H 6.16552300 5.05180300 5.25106300

C 3.66228200 0.85379600 1.26557000

O 2.49542600 0.57280700 1.54472900

O 4.27324200 0.41205600 0.18694100

H 3.64928400 -0.19710100 -0.32100000

H 0.84425700 -2.64489300 0.24639700

C -4.38954800 0.13103800 0.26440400

C -1.92212800 0.09179100 0.47872400

C -2.33318800 -0.68860700 -0.70179000

C -1.54621700 -1.39319100 -1.62534800

C -2.17104000 -2.08909000 -2.66194900

C -3.56541000 -2.08332100 -2.78486600

C -4.35763900 -1.38958000 -1.86214600

C -3.73870100 -0.70300200 -0.81816300

H -0.46204300 -1.43649900 -1.54049800

H -1.55908900 -2.64770500 -3.37393400

H -4.04233400 -2.63034100 -3.60193300

H -5.44305800 -1.38726100 -1.96163600

C -0.73804300 0.56897200 0.88611400

C -0.35646600 1.23368500 2.12314200

C -0.11819000 2.62267600 2.18610100

C -0.22883400 0.45557800 3.29616100

C 0.18295600 3.22329300 3.40362400

H -0.18208300 3.21542800 1.27287500

C 0.07519200 1.06581400 4.51145500

H -0.36828600 -0.62492700 3.23835900

C 0.27576800 2.44915500 4.56881800

H 0.35021800 4.30187900 3.44839900

H 0.16005800 0.45838400 5.41549200

H 0.51619700 2.92657200 5.52223900

C -3.22452300 0.27339900 1.32773400

H -4.53966900 1.14851900 -0.13333200

C -3.25470900 -0.83165100 2.30822100

C -3.34056800 1.55835300 2.03165400

N -3.23528400 -1.73100900 3.03722200

N -3.49182400 2.58130400 2.55127400

C -9.16468900 0.19622300 -1.69722200

C -8.42541700 1.30034300 -1.25849100

C -7.31602900 1.11782400 -0.42948800

C -6.92085100 -0.16907900 -0.02745500

C -7.67474000 -1.26753600 -0.46782600

C -8.78781000 -1.08854600 -1.29613100

H -10.03294600 0.33823400 -2.34585900

H -8.71667600 2.30992200 -1.56031700

H -6.75200900 1.98981900 -0.08497800

H -7.38369400 -2.27615600 -0.16108400

H -9.36150300 -1.95770600 -1.62878600

C -5.71559600 -0.36102900 0.87052500

H -5.88371000 0.19786600 1.80732300

H -5.61857200 -1.42087400 1.14930500

TS10-3b

E (SMD/B3LYP-D3/def2-SVP) = -4005.302939

H (SMD/B3LYP-D3/def2-SVP) = -4004.605908

G (SMD/B3LYP-D3/def2-SVP) = -4004.746096

E (SMD/B3LYP-D3/def2-TZVP//SMD/B3LYP-D3/def2-SVP) = -4007.813297

C 3.82649300 -1.60898600 2.45510200

C 1.81537600 -0.98133000 1.16754500

C 2.99246500 -0.40413300 0.54114200

C 3.07358500 0.46689200 -0.55574100

C 4.33326700 0.86021800 -1.00381000

C 5.49603500 0.39777400 -0.36857300

C 5.41703800 -0.44643100 0.74533300

C 4.15988000 -0.83698400 1.20074200

H 2.17034900 0.83186000 -1.03895300

H 4.41415500 1.53666700 -1.85785200

H 6.47548100 0.71266000 -0.73716000

H 6.32121400 -0.77502400 1.26034500

C 0.50958500 -0.70795000 1.04373300

C -0.69437700 -1.41135200 1.40771700

C -1.01900000 -2.63282100 0.76228500

C -1.50777700 -0.94896300 2.47189800

C -2.10300100 -3.38145100 1.20075200

H -0.40064800 -2.96918600 -0.06941800

C -2.59321300 -1.70914300 2.89872100

H -1.27036100 -0.00595000 2.96462700

C -2.89016800 -2.92229400 2.26847300

H -2.34061500 -4.32914900 0.71228200

H -3.20829600 -1.35438900 3.72823600

H -3.74373700 -3.51524000 2.60703500

C 2.34128800 -2.07624700 2.15916700

H 4.47130400 -2.48857900 2.59585500

C 1.52914000 -2.24284400 3.37313900

C 2.33964000 -3.36006200 1.42817300

N 0.89669800 -2.37729500 4.33255300

N 2.32807400 -4.35316700 0.83283300

C 7.83275900 0.98325400 4.26057000

C 7.58653200 -0.34757400 4.61686900

C 6.30693800 -0.88801900 4.46182300

C 5.25667300 -0.11144500 3.94609500

C 5.51434600 1.22209900 3.59303200

C 6.79210700 1.76679700 3.75215900

H 8.83244300 1.40841800 4.38357300

H 8.39301900 -0.96499400 5.02170600

H 6.11935500 -1.92800600 4.74674600

H 4.70532800 1.83234600 3.18245100

H 6.97473600 2.80806600 3.47355900

C 3.89110600 -0.71648900 3.72511300

H 3.61107600 -1.33036500 4.59500000

H 3.13841400 0.08204600 3.62652300

Cu -0.07512200 1.25106500 0.84675600

O -1.70902000 2.11731400 1.92536300

H -1.51876500 3.02673000 2.19890100

I 1.65082300 2.67197200 2.15755800

C -3.94709600 0.19921800 -1.16547500

C -4.24061500 -1.04956200 -0.59811300

C -4.44190700 -2.15991500 -1.41601700

C -4.37984100 -2.05014900 -2.81577700

C -4.11695700 -0.78681300 -3.37424400

C -3.89503700 0.32253800 -2.56388500

H -4.28187700 -1.14011800 0.48831000

H -4.64465900 -3.13313400 -0.96141200

H -4.06792100 -0.67693100 -4.46040500

H -3.68535000 1.29287900 -3.01490900

C -4.56421100 -3.24822700 -3.70934100

H -3.62716200 -3.47920500 -4.24503100

H -4.85942400 -4.14380800 -3.14288400

H -5.33240100 -3.05892500 -4.47723100

C -3.69270000 1.36131600 -0.27516000

O -2.95586000 2.36151200 -0.77099500

O -4.11307300 1.42495300 0.87025900

C -0.60906900 0.07103000 -3.18057600

C -0.56654700 -1.14671800 -3.87642200

C -0.88358200 -1.19085400 -5.23188000

C -1.22995500 -0.02296800 -5.93581700

C -1.24971500 1.19335700 -5.23275700

C -0.94658400 1.24302800 -3.87200400

H -0.29718700 -2.05589500 -3.33642600

H -0.86208500 -2.14877200 -5.75940900

H -1.50915000 2.11548200 -5.76013100

H -0.96939300 2.19575100 -3.33972800

C -1.53940000 -0.07827200 -7.40928200

H -2.11537000 -0.98110200 -7.66743100

H -2.10948700 0.80246100 -7.74143200

H -0.60762200 -0.11081100 -8.00122400

C -0.30476600 0.09827600 -1.71690100

O -0.69139300 1.12557900 -1.04666500

O 0.31228200 -0.85319600 -1.20540100

H -2.14233500 1.97891100 -1.19683500

H -2.65478500 2.07168800 1.64910300

9

E (SMD/B3LYP-D3/def2-SVP) = -1071.58439

H (SMD/B3LYP-D3/def2-SVP) = -1071.218033

G (SMD/B3LYP-D3/def2-SVP) = -1071.297343

E (SMD/B3LYP-D3/def2-TZVP//SMD/B3LYP-D3/def2-SVP) = -1072.742414

C -1.60319700 -3.87959800 0.05331400

C 0.15841800 -2.31903100 -0.76411800

C 0.06907000 -3.49648100 -1.64769500

C 0.79839900 -3.75132800 -2.81418500

C 0.56428300 -4.94154700 -3.50597100

C -0.38799200 -5.85788700 -3.03752600

C -1.12277000 -5.59742400 -1.87423600

C -0.88790400 -4.41149700 -1.17508700

H 1.54153700 -3.03092500 -3.16434500

H 1.12974400 -5.16184500 -4.41492900

H -0.56232600 -6.78679300 -3.58644400

H -1.87106200 -6.31025800 -1.52738300

C 0.77580200 -1.17546200 -0.92613000

C 1.27962300 0.09969200 -0.73163800

C 0.54220000 1.25617600 -1.14315400

C 2.56584200 0.29451400 -0.13219600

C 1.06727800 2.52635400 -0.95293200

H -0.44161700 1.12177400 -1.59722900

C 3.06897200 1.57539400 0.04646200

H 3.13742500 -0.57913800 0.18810900

C 2.32950400 2.69808600 -0.36083700

H 0.48897100 3.40007100 -1.26443900

H 4.04898200 1.70862500 0.51200700

H 2.73406000 3.70260300 -0.21548900

C -0.65192100 -2.70673300 0.53258000

H -2.52852600 -3.38471200 -0.28604300

C 0.31562600 -3.17125200 1.54466500

C -1.42194000 -1.58094600 1.07580400

N 1.09124500 -3.55144700 2.31564500

N -2.05529500 -0.69883700 1.47683000

C -5.25826500 -7.52041500 0.21183100

C -5.46031900 -6.13691500 0.26902000

C -4.39898700 -5.28484400 0.58372500

C -3.11603500 -5.79621800 0.84198200

C -2.92835200 -7.18585200 0.78935000

C -3.98911000 -8.04262700 0.47676400

H -6.08827500 -8.18783400 -0.03407200

H -6.45121200 -5.71932100 0.07157500

H -4.57213000 -4.20550900 0.63465200

H -1.93692500 -7.60113500 0.99073600

H -3.82128400 -9.12233400 0.44021200

C -1.96706300 -4.86727500 1.17625000

H -2.23983600 -4.27283300 2.06527800

H -1.07709000 -5.45282600 1.45040200

---------------------------------------------------------------

11

E (SMD/B3LYP-D3/def2-SVP) = -2473.822514

H (SMD/B3LYP-D3/def2-SVP) = -2473.649433

G (SMD/B3LYP-D3/def2-SVP) = -2473.712619

E (SMD/B3LYP-D3/def2-TZVP//SMD/B3LYP-D3/def2-SVP) = -2474.677639

Cu -0.24515300 -1.05975100 0.12474400

O -0.23160100 0.90903900 0.22219800

H 0.16161100 1.36950500 -0.53623200

I 1.71278600 -2.48536200 -0.31316500

C -4.43583200 -0.74846600 0.39305600

C -5.45823000 0.21525800 0.38269200

C -6.79243900 -0.17759600 0.44419400

C -7.14709800 -1.53763500 0.51831900

C -6.11702600 -2.49209100 0.53089700

C -4.77743600 -2.10677000 0.46836200

H -5.18712000 1.27107500 0.32584600

H -7.57993500 0.58174700 0.43540000

H -6.36932600 -3.55445100 0.59069200

H -3.98363600 -2.85554200 0.47817800

C -8.59704300 -1.94420600 0.56908500

H -9.13676100 -1.39878700 1.36085300

H -9.10407800 -1.70919300 -0.38286100

H -8.71495000 -3.02212200 0.75472800

C -3.00816800 -0.31432400 0.31964500

O -2.12802500 -1.25740500 0.33059200

O -2.72995300 0.90226800 0.25075700

H -1.25743200 1.09327900 0.22462900

11a

E (SMD/B3LYP-D3/def2-SVP) = -2473.800857

H (SMD/B3LYP-D3/def2-SVP) = -2473.627463

G (SMD/B3LYP-D3/def2-SVP) = -2473.690397

E (SMD/B3LYP-D3/def2-TZVP//SMD/B3LYP-D3/def2-SVP) = -2474.662404

Cu -0.00634000 -1.43882500 0.31151000

O -0.03765600 0.50256100 0.67619300

H 0.58414300 1.01016600 0.12986700

I 0.03506129 -4.00543266 -0.17061884

C 4.32151163 -1.73933550 -0.45807240

C 5.42093006 -2.61582705 -0.45934207

C 6.71598115 -2.10638283 -0.42557658

C 6.95070359 -0.71944311 -0.39092434

C 5.84201288 0.14598518 -0.39741682

C 4.54228940 -0.35165281 -0.42832803

H 5.25190730 -3.69346149 -0.48734838

H 7.56556484 -2.79471066 -0.42695289

H 6.00372814 1.22704812 -0.38002911

H 3.68480360 0.32382745 -0.43761115

C 8.35406716 -0.17948949 -0.31794921

H 8.69880856 -0.14468157 0.73098979

H 9.06305243 -0.81525206 -0.87059072

H 8.41881562 0.84387562 -0.71739637

C 2.93764426 -2.25024601 -0.48646274

O 1.97350385 -1.46558072 -0.38806491

O 2.79596145 -3.54163183 -0.61813440

H 1.80893662 -3.78189196 -0.53895506

12

E (SMD/B3LYP-D3/def2-SVP) = -2933.702985

H (SMD/B3LYP-D3/def2-SVP) = -2933.374419

G (SMD/B3LYP-D3/def2-SVP) = -2933.461625

E (SMD/B3LYP-D3/def2-TZVP//SMD/B3LYP-D3/def2-SVP) = -2935.061559

Cu -0.00634000 -1.43882500 0.31151000

O -0.03765600 0.50256100 0.67619300

H 0.58414300 1.01016600 0.12986700

I 2.40844500 -1.32666100 -0.67736300

C -4.33077600 -0.85837200 0.41093100

C -5.27821200 0.10832100 0.03194000

C -6.63875000 -0.17760600 0.09350500

C -7.09931800 -1.42664700 0.55069100

C -6.14657100 -2.37964900 0.94306900

C -4.77996000 -2.10395000 0.87321300

H -4.92634700 1.08245200 -0.31271400

H -7.36429000 0.58105400 -0.21430700

H -6.47964400 -3.35322400 1.31289100

H -4.06099600 -2.85779700 1.19657600

C -8.57665300 -1.71487700 0.61476300

H -9.09339500 -0.98784600 1.26387500

H -9.03816200 -1.63436400 -0.38406700

H -8.78219400 -2.72329900 1.00325700

C -2.87613600 -0.51946800 0.34536600

O -2.03727100 -1.51107800 0.41305400

O -2.52138300 0.66523800 0.23453500

C -0.45124300 -5.72062800 0.26566100

C -1.42683500 -6.72281500 0.12143400

C -1.05481600 -8.06397000 0.14856300

C 0.28996100 -8.44188400 0.31783500

C 1.25637600 -7.42906200 0.45361800

C 0.89618500 -6.08458400 0.43078100

H -2.47291300 -6.44241600 -0.01178100

H -1.81956100 -8.83726500 0.03556100

H 2.30772800 -7.70217600 0.57698300

H 1.65016900 -5.30172900 0.53221000

C 0.67715400 -9.89524400 0.38210700

H 0.56290200 -10.27862800 1.41181300

H 0.03852200 -10.51471400 -0.26648600

H 1.72656000 -10.05126100 0.08981900

C -0.81650900 -4.29122700 0.24782800

O 0.04426300 -3.41745200 0.47263300

O -2.06538800 -4.01224100 -0.01208900

H -2.21398400 -3.00909000 0.08700500

H -0.99989800 0.77125300 0.45575900

12a

E (SMD/B3LYP-D3/def2-SVP) = -2933.693061

H (SMD/B3LYP-D3/def2-SVP) = -2933.365286

G (SMD/B3LYP-D3/def2-SVP) = -2933.454435

E (SMD/B3LYP-D3/def2-TZVP//SMD/B3LYP-D3/def2-SVP) = -2935.054625

Cu 0.16401800 -1.19935100 0.45948500

O 0.01461800 0.64101400 1.14485900

H 0.48892300 1.30262700 0.61677800

I 2.50149900 -0.78595900 -0.68368000

C -4.10377000 -1.00766500 0.47804200

C -5.16107900 -0.09507700 0.62508800

C -6.47820500 -0.51038400 0.44457000

C -6.78074700 -1.84378400 0.11172300

C -5.71636500 -2.74838000 -0.03214500

C -4.39403300 -2.33912500 0.14776000

H -4.92945000 0.94033400 0.88169900

H -7.29298100 0.21038300 0.56211200

H -5.92799000 -3.78988800 -0.29064500

H -3.57081300 -3.04620400 0.03148100

C -8.21189200 -2.27164600 -0.08965100

H -8.82640900 -2.03473700 0.79510700

H -8.66605100 -1.74093700 -0.94408100

H -8.29450600 -3.35178700 -0.28197300

C -2.68776600 -0.55177200 0.66486000

O -1.78518000 -1.45233200 0.54009600

O -2.46246400 0.65340400 0.92627300

H -1.01630800 0.81896100 1.08610800

C 0.06686300 -5.48925300 0.03535500

C 0.54897200 -6.59925900 -0.68166500

C 0.01791900 -7.86253900 -0.44149600

C -1.00196100 -8.05503700 0.50989900

C -1.47790000 -6.93592200 1.21524100

C -0.95217800 -5.66692700 0.98649300

H 1.33908800 -6.46462400 -1.42216100

H 0.39819600 -8.72178800 -1.00076200

H -2.27372100 -7.06368400 1.95364900

H -1.32608300 -4.79861000 1.53229400

C -1.54779500 -9.43361300 0.76956700

H -1.77480200 -9.95765700 -0.17294700

H -0.80517400 -10.04895100 1.30699500

H -2.46288700 -9.40531800 1.37913100

C 0.60006800 -4.13343600 -0.18978200

O 0.25846300 -3.18752700 0.53394400

O 1.44084900 -4.00573500 -1.19050100

H 1.80094800 -3.08058800 -1.21284000

13

E (SMD/B3LYP-D3/def2-SVP) = -2857.300248

H (SMD/B3LYP-D3/def2-SVP) = -2856.999856

G (SMD/B3LYP-D3/def2-SVP) = -2857.08257

E (SMD/B3LYP-D3/def2-TZVP//SMD/B3LYP-D3/def2-SVP) = -2858.568204

Cu -0.29146200 -1.54808700 0.30557800

I 1.72385200 0.00442700 0.09604300

C -4.13287400 -1.03492200 0.52533500

C -4.65083300 0.27161200 0.50509000

C -6.02408700 0.47764000 0.59792500

C -6.91477100 -0.60591600 0.71353200

C -6.38415800 -1.90780600 0.72861300

C -5.01055200 -2.12475200 0.63728600

H -3.96320200 1.11464500 0.41495200

H -6.41878000 1.49728100 0.58007800

H -7.06077800 -2.76256700 0.81251000

H -4.60739400 -3.13916300 0.64958400

C -8.39568600 -0.36715300 0.84638400

H -8.65221100 -0.10080900 1.88716500

H -8.72830600 0.46776200 0.20968400

H -8.98055100 -1.26062900 0.58099400

C -2.67641200 -1.24986700 0.43057800

O -2.17132200 -2.42904200 0.46474200

O -1.87001100 -0.27999500 0.31700000

C 1.19581600 -5.59829300 0.41122900

C 0.73511900 -6.92532900 0.47104200

C 1.64466300 -7.97898000 0.43875300

C 3.02827500 -7.74212500 0.34830700

C 3.47554500 -6.40943500 0.28783900

C 2.57639400 -5.34793900 0.31922300

H -0.33551300 -7.12419400 0.53865300

H 1.27742900 -9.00783800 0.48195300

H 4.54684300 -6.20497300 0.21218600

H 2.92764300 -4.31556900 0.26904800

C 4.00865100 -8.88430100 0.34648900

H 4.33235700 -9.11466900 1.37723700

H 3.56476800 -9.80183700 -0.06888400

H 4.91368900 -8.64170700 -0.23127600

C 0.25874000 -4.45944700 0.43266800

O 0.67938500 -3.28957500 0.37770600

O -1.01296200 -4.77025600 0.51067700

H -1.58249700 -3.95517200 0.51333600

13a

E (SMD/B3LYP-D3/def2-SVP) = -2857.298198

H (SMD/B3LYP-D3/def2-SVP) = -2856.997367

G (SMD/B3LYP-D3/def2-SVP) = -2857.082185

E (SMD/B3LYP-D3/def2-TZVP//SMD/B3LYP-D3/def2-SVP) = -2858.568321

Cu -0.59593200 -2.13930300 0.37370100

I 1.72919500 -1.02180700 0.14143600

C -4.16322200 -0.70619200 0.61866100

C -4.35234500 0.68507900 0.60499500

C -5.63760200 1.21463800 0.69767500

C -6.76186700 0.37627200 0.80664200

C -6.55832100 -1.01530900 0.81591400

C -5.27679300 -1.55432600 0.72482400

H -3.48261500 1.33945000 0.51942600

H -5.77612700 2.29929600 0.68428500

H -7.42041400 -1.68353400 0.89471400

H -5.12220000 -2.63506600 0.73182700

C -8.14573200 0.95617100 0.94190300

H -8.38249900 1.14950900 2.00345600

H -8.23851800 1.91531400 0.40930100

H -8.91492300 0.26954300 0.55626200

C -2.80353700 -1.27695400 0.52079400

O -2.60121500 -2.53141100 0.54668100

O -1.77929500 -0.52181800 0.40866600

C 0.70325600 -6.27721500 0.20374400

C 1.80478200 -7.14035100 0.07085800

C 1.60483000 -8.51721400 0.01214600

C 0.31251600 -9.06782600 0.08040600

C -0.78074200 -8.19232100 0.21806800

C -0.59386600 -6.81515700 0.27889200

H 2.81334900 -6.72785800 0.01503000

H 2.46708600 -9.18164600 -0.08897900

H -1.79274200 -8.60168000 0.27792500

H -1.44411000 -6.13861400 0.38440400

C 0.09811000 -10.55400200 -0.02409500

H -0.10692400 -10.83922000 -1.07143500

H 0.98482900 -11.11874500 0.30126600

H -0.76493400 -10.88238900 0.57542300

C 0.87605900 -4.81309900 0.25715000

O -0.10431300 -4.06159900 0.38365600

O 2.10892200 -4.38147100 0.15935700

H 2.13251500 -3.38480400 0.17819800

12b

E (SMD/B3LYP-D3/def2-SVP) = -2933.690254

H (SMD/B3LYP-D3/def2-SVP) = -2933.362341

G (SMD/B3LYP-D3/def2-SVP) = -2933.450613

E (SMD/B3LYP-D3/def2-TZVP//SMD/B3LYP-D3/def2-SVP) = -2935.051926

Cu -0.12307000 -1.92944100 0.21157400

O 0.14781500 0.07690700 0.13839200

H 0.68541500 0.33679000 -0.62483900

I -0.42818600 -4.55182800 0.24475500

C -4.22653600 -0.85645900 0.37650100

C -5.09420200 0.23532900 0.20805600

C -6.47031800 0.06577300 0.33737700

C -7.02183200 -1.19405300 0.63604200

C -6.14560100 -2.27871400 0.80107600

C -4.76481900 -2.11636900 0.67425500

H -4.66866800 1.21339600 -0.02409500

H -7.13579000 0.92434200 0.20561600

H -6.55213400 -3.26720600 1.03307200

H -4.08883800 -2.96343200 0.80324200

C -8.51449900 -1.35870700 0.76584100

H -8.92698000 -0.66608600 1.51877400

H -9.02160800 -1.13127200 -0.18764500

H -8.79086200 -2.38262400 1.05847400

C -2.74671900 -0.66041400 0.23413600

O -2.02770900 -1.69948500 0.47179500

O -2.30762900 0.46404200 -0.09160700

C 4.22185700 -2.04700900 -0.27021400

C 5.36663200 -2.84143300 -0.45496000

C 6.61724900 -2.24065000 -0.57359800

C 6.76248800 -0.84334500 -0.50518900

C 5.60864700 -0.06022000 -0.31629900

C 4.35353300 -0.64909800 -0.20023300

H 5.26919100 -3.92676700 -0.51059600

H 7.50149100 -2.86577500 -0.72371100

H 5.70126100 1.02759600 -0.25917100

H 3.46154700 -0.03895000 -0.04629200

C 8.12148600 -0.20301800 -0.59828800

H 8.56522900 -0.09607500 0.40764600

H 8.81679300 -0.80935200 -1.19854400

H 8.06712000 0.80451700 -1.03821300

C 2.87956500 -2.64945100 -0.14791400

O 1.86703800 -1.93294600 -0.08346900

O 2.84420900 -3.95638800 -0.11326600

H 1.90046100 -4.27519100 -0.02468600

H -0.81617800 0.42130200 -0.00127800

**8. References**

1. (a) L. Zou, L. Wang, L. Sun, X. Xie, P. Li, *Chem. Commun.* **2020,** *56*, 7933-7936. (b) X. Xie, L. Wang, Q. Zhou, Y. Ma, Z.-M. Wang, P. Li, *Chin. Chem. Lett.* **2022,** *33*, 5069-5073.

2- Gaussian 16, Revision C.01, M. J. Frisch, G. W. Trucks, H. B. Schlegel, G. E. Scuseria, M. A. Robb, J. R. Cheeseman, G. Scalmani, V. Barone, G. A. Petersson, H. Nakatsuji, X. Li, M. Caricato, A. V. Marenich, J. Bloino, B. G. Janesko, R. Gomperts, B. Mennucci, H. P. Hratchian, J. V. Ortiz, A. F. Izmaylov, J. L. Sonnenberg, D. Williams-Young, F. Ding, F. Lipparini, F. Egidi, J. Goings, B. Peng, A. Petrone, T. Henderson, D. Ranasinghe, V. G. Zakrzewski, J. Gao, N. Rega, G. Zheng, W. Liang, M. Hada, M. Ehara, K. Toyota, R. Fukuda, J. Hasegawa, M. Ishida, T. Nakajima, Y. Honda, O. Kitao, H. Nakai, T. Vreven, K. Throssell, J. A. Montgomery, Jr., J. E. Peralta, F. Ogliaro, M. J. Bearpark, J. J. Heyd, E. N. Brothers, K. N. Kudin, V. N. Staroverov, T. A. Keith, R. Kobayashi, J. Normand, K. Raghavachari, A. P. Rendell, J. C. Burant, S. S. Iyengar, J. Tomasi, M. Cossi, J. M. Millam, M. Klene, C. Adamo, R. Cammi, J. W. Ochterski, R. L. Martin, K. Morokuma, O. Farkas, J. B. Foresman, and D. J. Fox, Gaussian, Inc., Wallingford CT, **2016.**

3- a) A. D. Becke, *J. Chem. Phys.* **1993**, *98*, 5648-5652; b) A. D. Becke, *J. Chem. Phys.* **1992**, *96*, 2155−2160; c) C. Lee, W. Yang, R. G. Parr, *Phys. Rev. B*. **1988**, *37*, 785-789.

4- A. V. Marenich, C. J. Cramer, D. G. Truhlar, *J. Phys. Chem. B.* **2009**, *113*, 6378-6396.

5- S. Grimme, *J. Comput. Chem.* **2006,** *27*, 1787-1799.

6- K. Fukui, *Acc. Chem. Res.* **1981,** *14*, 363-368.
